# Comprehensive evaluation of time-varied outcomes for invasive and conservative strategies in patients with NSTE-ACS: a meta-analysis of randomized controlled trials

**DOI:** 10.3389/fcvm.2023.1197451

**Published:** 2023-09-08

**Authors:** Yi-Jing Zhao, Yangyang Sun, Fan Wang, Yuan-Yuan Cai, Raphael N. Alolga, Lian-Wen Qi, Pingxi Xiao

**Affiliations:** ^1^ State Key Laboratory of Natural Medicines, School of Traditional Chinese Pharmacy, China Pharmaceutical University, Nanjing, China; ^2^ The Clinical Metabolomics Center, China Pharmaceutical University, Nanjing, China; ^3^ Department of Pharmacy, Children’s Hospital, Zhejiang University School of Medicine, National Clinical Research Center for Child Health, Hangzhou, China; ^4^ School of International Pharmaceutical Business, China Pharmaceutical University, Nanjing, China; ^5^ College of Traditional Chinese Medicine and Food Engineering, Shanxi University of Chinese Medicine, Taiyuan, China; ^6^ Department of Cardiology, The Fourth Affiliated Hospital of Nanjing Medical University, Nanjing, China

**Keywords:** time-varied outcomes, meta-analysis, randomized controlled trials, non-ST-elevation acute coronary syndrome, invasive strategy, conservative strategy

## Abstract

**Background:**

Results from randomized controlled trials (RCTs) and meta-analyses comparing invasive and conservative strategies in patients with non-ST-elevation acute coronary syndrome (NSTE-ACS) are highly debatable. We systematically evaluate the efficacy of invasive and conservative strategies in NSTE-ACS based on time-varied outcomes.

**Methods:**

The RCTs for the invasive versus conservative strategies were identified by searching PubMed, Cochrane Central Register of Controlled Trials, Embase, and ClinicalTrials.gov. Trial data for studies with a minimum follow-up time of 30 days were included. We categorized the follow-up time into six varied periods, namely, ≤6 months, 1 year, 2 years, 3 years, 5 years, and ≥10 years. The time-varied outcomes were major adverse cardiovascular event (MACE), death, myocardial infarction (MI), rehospitalization, cardiovascular death, bleeding, in-hospital death, and in-hospital bleeding. Risk ratios (RRs) and 95% confidence intervals (Cis) were calculated. The random effects model was used.

**Results:**

This meta-analysis included 30 articles of 17 RCTs involving 12,331 participants. We found that the invasive strategy did not provide appreciable benefits for NSTE-ACS in terms of MACE, death, and cardiovascular death at all time points compared with the conservative strategy. Although the risk of MI was reduced within 6 months (RR 0.80, 95% CI 0.68–0.94) for the invasive strategy, no significant differences were observed in other periods. The invasive strategy reduced the rehospitalization rate within 6 months (RR 0.69, 95% CI 0.52–0.90), 1 year (RR 0.73, 95% CI 0.63–0.86), and 2 years (RR 0.77, 95% CI 0.60–1.00). Of note, an increased risk of bleeding (RR 1.80, 95% CI 1.28–2.54) and in-hospital bleeding (RR 2.17, 95% CI 1.52–3.10) was observed for the invasive strategy within 6 months. In subgroups stratified by high-risk features, the invasive strategy decreased MACE for patients aged ≥65 years within 6 months (RR 0.68, 95% CI 0.58–0.78) and 1 year (RR 0.75, 95% CI 0.62–0.91) and showed benefits for men within 6 months (RR 0.71, 95% CI 0.55–0.92). In other subgroups stratified according to diabetes, ST-segment deviation, and troponin levels, no significant differences were observed between the two strategies.

**Conclusions:**

An invasive strategy is superior to a conservative strategy in reducing early events for MI and rehospitalizations, but the invasive strategy did not improve the prognosis in long-term outcomes for patients with NSTE-ACS.

**Systematic Review Registration:**

https://www.crd.york.ac.uk/prospero/display_record.php?ID=CRD42021289579, identifier PROSPERO 2021 CRD42021289579.

## Graphical Abstract

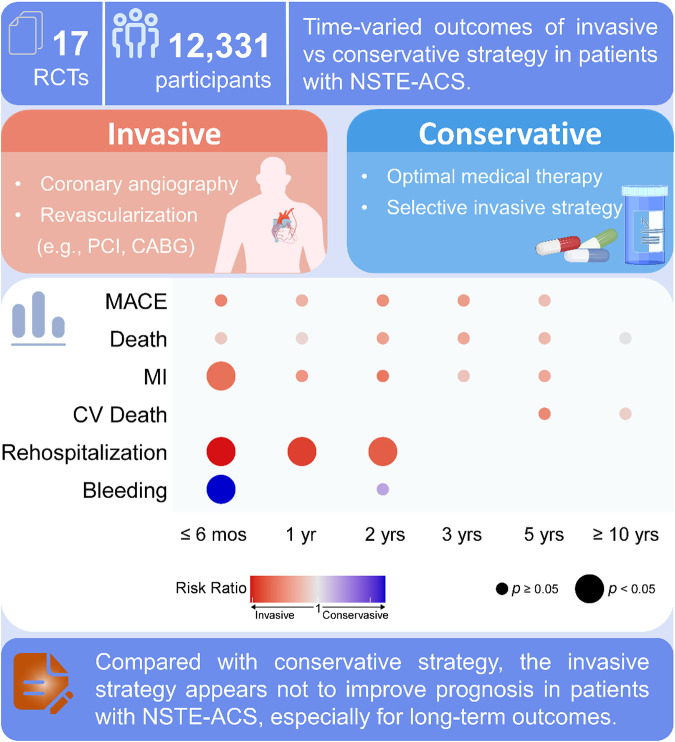

## 1. Introduction

Non-ST-elevation acute coronary syndrome (NSTE-ACS) constitutes almost three-fourths of all ACS, and its prevalence has gradually increased over the past decade (1, 2). The initial therapeutic options consist of either a conservative or an invasive strategy with coronary angiography followed by revascularization (3). Uncertainty remains as to which strategy provides better outcomes for these patients. Seven trials with different follow-up times (4–19) indicated that an invasive treatment strategy was preferable to a conservative strategy. The invasive strategy may be beneficial for those patients who are at high risk for recurrent events (20). With advances in interventional therapy, most patients are treated aggressively. However, several randomized controlled trials (RCTs) failed to show appreciable benefits in reducing major cardiac events with the invasive strategy compared with the conservative management (21–32). The invasive strategy also carries a certain risk for procedure-related myocardial infarction (MI) and a high risk of bleeding (20). In addition, the extensive use of iodinated contrast media in invasive procedures was linked to acute kidney injury and subsequent all-cause deaths (33).

Previous meta-analyses have compared the effects of the two strategies on patients with NSTE-ACS, but their conclusions are controversial (34–38). These inconsistencies can be attributable to the small sample sizes, varied follow-up times, and endpoints. In addition, temporal assessment of short- to long-term major adverse cardiovascular events (MACE) shows a progressive decline in clinical benefits between invasive and conservative treatment strategies (39). A comprehensive evaluation of the time-varied outcomes of these two strategies can offer the needed guidance in the clinical treatment of NSTE-ACS. Therefore, a systematic meta-analysis that provides an update of the benefits of invasive therapy for NSTE-ACS is urgently needed.

In this regard, we performed a comprehensive meta-analysis of the time-varied outcomes of the invasive and conservative strategies based on RCTs. We included 17 RCTs involving 12,331 participants. The follow-up time was categorized into six different periods, namely, ≤6 months, 1 year, 2 years, 3 years, 5 years, and ≥10 years. The time-varied outcomes included MACE, death, MI, rehospitalization, cardiovascular (CV) death, bleeding, in-hospital death, and in-hospital bleeding.

## 2. Methods

### 2.1. Search strategy

We identified RCTs of potential interest by searching PubMed, Cochrane Central Register of Controlled Trials (CENTRAL), Embase, and ClinicalTrials.gov (up to 30 October 2021), without language restrictions. The keywords used for the search included (“acute coronary syndrome” OR “non-ST-segment elevation acute coronary syndrome” OR “non-ST-elevation myocardial infarction” OR “unstable angina” OR “NSTE-ACS” OR “NSTEMI” OR “UA”) AND (“Management” OR “treatment” OR “therapy” OR “intervention” OR “delayed” OR “selective”) AND (“clinical trial” OR “randomized controlled trial”). This meta-analysis was performed in accordance with the Preferred Reporting Items for Systematic Reviews and Meta-Analyses (PRISMA) (40, 41) guidelines (Supplementary Table S1). This study was registered with PROSPERO (CRD42021289579).

### 2.2. Selection criteria

We included RCTs that compared invasive and conservative treatment strategies with a minimum follow-up time of 30 days for patients presenting with NSTE-ACS. An invasive strategy is an “early invasive” or “routine invasive” approach that triages patients to undergo an early coronary angiography, eventually followed by revascularization, without first undergoing a preliminary non-invasive stress testing or experiencing treatment failure with optimal medical care. A conservative strategy (also referred to as a selective or non-invasive strategy) consisted of optimal medical therapy and subsequent invasive evaluation only for those patients with residual symptoms or objective evidence of myocardial ischemia. The exclusion criteria included (1) non-randomized studies, (2) studies including patients with stable angina pectoris or ST-segment elevation MI, and (3) studies requiring coronary angiography for all patients in the conservative group.

### 2.3. Data extraction

Two investigators (YZ and PX) independently screened the titles and abstracts for eligibility and the full text and supplementary material to confirm the inclusion criteria and performed data extraction. Any disparities between the two investigators were discussed with a third investigator (LQ) until a consensus was reached. According to the Cochrane Collaboration risk-of-bias tool, two independent investigators (YZ and FW) assessed the risk of bias in the included trials. A consensus after discussion resolved the discrepancies.

We categorized the follow-up time into six periods (i.e., ≤6 months, 1 year, 2 years, 3 years, 5 years, and ≥10 years). The overall outcomes were MACE, death, MI, CV death, rehospitalization, bleeding, in-hospital death, and in-hospital bleeding. MACE was the trial-defined primary endpoint in the respective trials. Moreover, we evaluated the treatment effect in five subgroups stratified by high-risk features (i.e., age, gender, diabetes, ST-segment deviation, and elevated troponins). A novel universal definition of MI was proposed in 2007 and is now widely used worldwide (42). The recruited studies were also stratified by the year 2007 to detect the possible impact of the enrollment year on the two strategies for patients with NSTE-ACS. A pooled analysis was not performed for RCT studies less than three. Outcome data were independently extracted from each published study by two investigators (YZ and PX) and verified by the principal investigators in all included trials.

### 2.4. Statistical analysis

Risk ratios (RRs) and 95% confidence intervals (CIs) were calculated using a random effects model with R version 3.6.3. Cochran's *Q* test and Higgins’ *I*2 statistics were used to estimate study heterogeneity. Heterogeneity was considered significant if the *I*^2^ value was more than 50%. To explore whether or not a single study significantly affected the robustness of our findings, we performed a sensitivity analysis by sequentially removing each study from the pooled effect estimates. Meta-regression analyses were performed using the empirical Bayes (Paule–Mandel) method to evaluate the relation of covariates (e.g., proportion of patients with diabetes, hypertension, and hyperlipidemia) on the overall outcomes when the heterogeneity was more than 50%. The risk of bias was assessed using the Cochrane Collaboration risk-of-bias tool. The publication bias was assessed using the Harbord and Egger tests and funnel plots.

## 3. Results

### 3.1. Characteristics of the included studies

**Figure 1:**
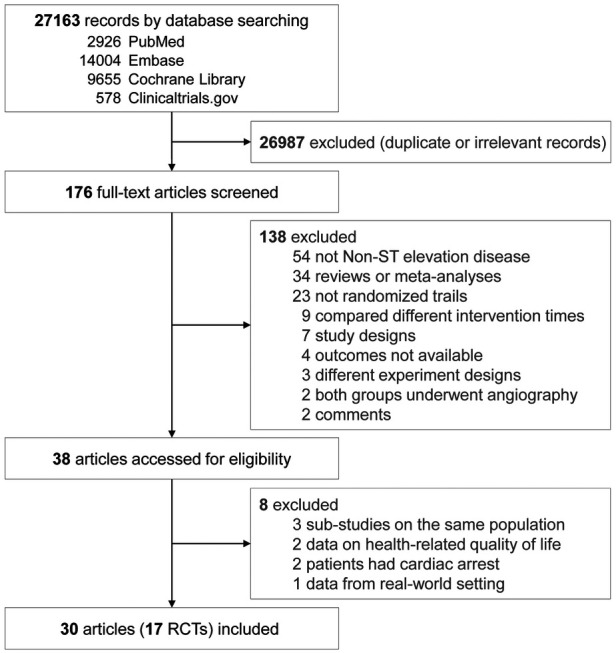
Study selection.

Our search retrieved 27,163 items, of which 26,987 duplicate or irrelevant records were excluded. After screening full texts, 38 articles remained and were then evaluated in detail. From these, three articles (43–45) belonging to sub-studies of three trials (11, 16, 29), two articles (46, 47) comparing health-related quality of life, two articles (48, 49) reporting patients with out-of-hospital cardiac arrest, and one article (50) analyzing real-world outcomes were excluded. Eventually, this meta-analysis included 30 articles from 17 RCTs involving 12,331 participants (Figure 1). Some articles reported data from different follow-up time points from the FRISC-II (14–19), ICTUS (25–28), RITA 3 (6–9), and TIMI IIIB (29, 30) trials.

Four RCTs (4, 21, 23, 24) focused on elderly participants aged 70, 75, 80 years, or older only at the baseline; one (51) included both participants with and without diabetes mellitus; and one (5) involved only women. Four trials (12, 18, 28, 29) reported age-stratified outcomes; six (9, 11, 17, 24, 28, 29) reported gender-stratified outcomes; three (11, 18, 28) reported diabetes-stratified outcomes; four (11, 18, 27, 28) reported outcomes with or without ST-segment deviation; and four (11, 18, 24, 28) reported outcomes of troponin levels above or within the normal range. Most studies were at low risk of bias (Supplementary Figure S1).

**Figure 2:**
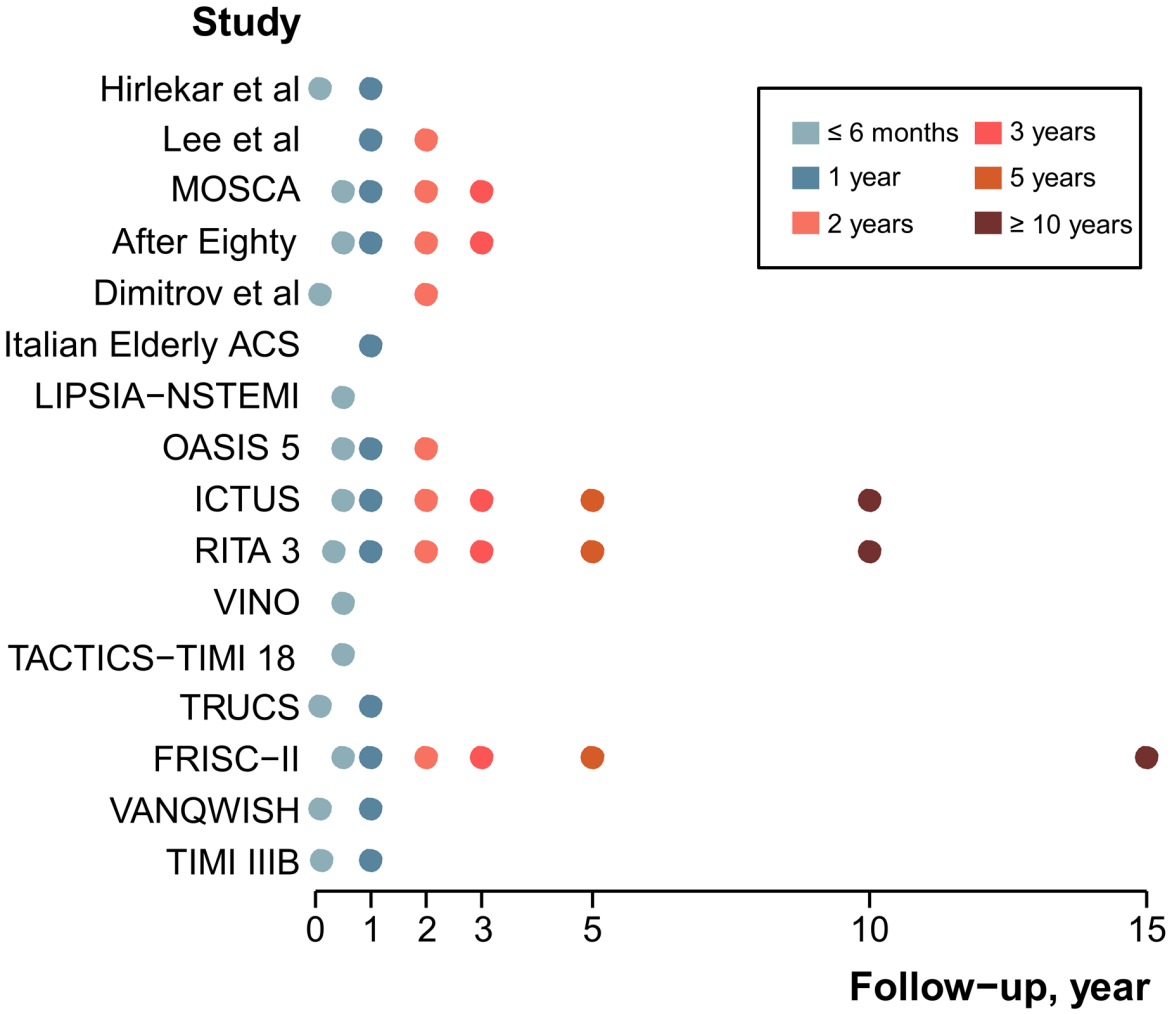
Distribution of the six specific time points in 17 RCTs.

The Appendix in the Supplementary material details the main features of the 17 RCTs (Supplementary Table S2) and the outcome definitions (Supplementary Table S3). Table 1 summarizes the baseline characteristics of the participants. The mean or median age of the RCTs ranged from 56 to 85 years. The proportion of males was mostly between 49% and 73%. The diabetes rates typically ranged from 12% to 46%. The proportion of previous MI incidence was generally less than 50%. Approximately 9%–25% of patients had undergone percutaneous coronary intervention (PCI), and 4%–18% underwent coronary artery bypass graft (CABG). The follow-up time points of the 17 RCTs ranged from 1 month to 15 years (Figure 2).

**Table 1:** Baseline characteristics of participants included in the analysis.

	Hirlekar et al. (21) (*N* = 186)	Lee et al. (22) (*N* = 60)	MOSCA (*N* = 106)	After Eighty (*N* = 457)	Dimitrov et al. (51) (DM) (*N* = 52)	Dimitrov et al. (51) (non-DM) (*N* = 126)	Italian Elderly ACS (*N* = 313)	LIPSIA-NSTEMI (*N* = 400)	OASIS 5 (*N* = 184)	ICTUS (*N* = 1,200)	Eisenberg et al. (*N* = 88)	RITA 3 (*N* = 1,810)	VINO (*N* = 131)	TACTICS-TIMI 18 (*N* = 2,220)	TRUCS (*N* = 148)	FRISC-II (*N* = 2,457)	VANQWISH (*N* = 920)	TIMI IIIB (*N* = 1,473)
Patients	93/93	31/29	52/54	229/228	22/30	54/72	154/159	200/200	92/92	604/596	42/46	895/915	64/67	1,114/1,106	76/72	1,222/1,235	462/458	740/733
Age (years)	84/84	69/73	81/83	85/85	63/65	61/62	82/82	70/70	68/68	62/62	57/56	63/62	65/66	62/62	62/63	66/65	62/61	59/59
Male, *n* (%)	47 (51)/55 (59)	24 (77)/19 (66)	29 (56)/27 (50)	125 (55)/100 (44)	12 (55)/25 (83)	43 (80)/45 (63)	76 (49)/81 (51)	139 (70)/128 (64)	0/0	446 (74)/434 (73)	32 (76)/40 (87)	545 (61)/583 (64)	41 (64)/39 (58)	709 (64)/744 (67)	57 (75)/51 (71)	874 (72)/834 (68)	448 (97)/448 (98)	491 (66)/485 (66)
Cardiovascular risk factors, *n* (%)																		
Diabetes	16 (17)/20 (22)	10 (32)/11 (38)	24 (46)/25 (46)	45 (20)/32 (14)	22 (100)/30 (100)	0/0	55 (36)/59 (37)	85 (43)/64 (33)	19 (21)/27 (29)	86 (14)/80 (13)	9 (21)/14 (30)	130 (15)/114 (12)	19 (30)/29 (43)	313 (28)/300 (27)	22 (29)/20 (28)	155 (13)/144 (12)	115 (25)/125 (27)	NA
Hypertension	55 (59)/59 (63)	23 (74)/19 (66)	49 (94)/45 (83)	131 (57)/139 (61)	NA	NA	136 (88)/123 (77)	161 (82)/165 (85)	57 (62)/63 (67)	226 (37)/240 (40)	23 (55)/16 (35)	315 (35)/317 (35)	38 (59)/29 (43)	NA	41 (54)/38 (53)	366 (30)/377 (31)	262 (57)/236 (52)	292 (39)/327 (45)
Dyslipidemia	21 (23)/16 (17)	NA	39 (75)/34 (63)	NA	21 (96)/26 (87)	51 (94)/46 (64)	65 (42)/72 (45)	82 (42)/67 (35)	NA	211 (35)/206 (35)	13 (31)/13 (28)	NA	NA	NA	40 (53)/40 (56)	683 (56)/685 (55)	80 (17)/77 (17)	NA
Smokers	35 (38)/42 (45)	27 (87)/18 (62)	4 (8)/2 (4)	112 (49)/109 (48)	12 (55)/9 (30)	27 (50)/31 (43)	NA	49 (25)/51 (26)	9 (10)/24 (26)	244 (40)/248 (42)	12 (29)/17 (37)	272 (30)/314 (34)	NA	NA	38 (50)/30 (42)	362 (30)/383 (31)	189 (41)/210 (46)	281 (38)/264 (36)
Clinical history, *n* (%)																		
Previous MI	29 (31)/35 (38)	23 (74)/18 (62)	24 (46)/23 (43)	107 (47)/90 (39)	9 (41)/11 (37)	26 (48)/31 (43)	43 (28)/54 (34)	46 (24)/39 (20)	22 (24)/18 (20)	153 (25)/125 (21)	8 (19)/9 (20)	267 (30)/234 (26)	14 (22)/20 (30)	437 (39)/429 (39)	NA	278 (23)/268 (22)	199 (43)/197 (43)	295 (40)/273 (37)
Previous PCI	15 (16)/16 (17)	NA	12 (23)/9 (17)	55 (24)/46 (20)	8 (36)/5 (17)	15 (28)/13 (18)	16 (10)/31 (19)	31 (16)/32 (17)	7 (8)/11 (12)	77 (13)/63 (11)	5 (12)/4 (9)	NA	NA	NA	12 (16)/10 (14)	NA	40 (9)/44 (10)	NA
Previous CABG	19 (20)/14 (15)	31 (100)/29 (100)	10 (19)/4 (7)	44 (19)/32 (14)	NA	NA	17 (11)/12 (8)	15 (8)/16 (8)	5 (5)/3 (3)	62 (10)/43 (7)	0 (0)/7 (15)	NA	NA	NA	10 (13)/8 (11)	NA	88 (19)/68 (15)	NA
During index hospitalization, *n* (%)																		
CAG	89 (96)/4 (4)	31 (100)/0 (0)	52 (100)/11 (20)	220 (96)/NA	NA	NA	136 (88)/46 (29)	198 (99)/170 (85)	88 (96)/37 (40)	593 (98)/314 (53)	35 (83)/15 (33)	857 (96)/142 (16)	64 (100)/NA	1,085 (97)/561 (51)	76 (100)/38 (53)	1,173 (96)/123 (10)	435 (94)/110 (24)	724 (98)/420 (57)
PCI	57 (61)/3 (3)	10 (32)/0 (0)	28 (54)/4 (7)	107 (47)/NA	NA	NA	76 (49)/35 (22)	141 (71)/114 (57)	42 (46)/22 (24)	361 (60)/169 (28)	6 (14)/6 (13)	291 (33)/62 (7)	NA	459 (41)/262 (24)	40 (53)/23 (32)	NA	NA	NA
CABG	1 (1)/1 (1)	NA	2 (4)/1 (2)	6 (3)/NA	NA	NA	9 (6)/1 (1)	25 (13)/25 (13)	11 (12)/6 (7)	97 (16)/68 (11)	4 (10)/4 (9)	106 (12)/33 (4)	NA	220 (20)/142 (13)	19 (25)/4 (6)	NA	NA	NA
ST depression	35 (38)/39 (42)	14 (45)/14 (48)	21 (40)/26 (48)	43 (19)/40 (18)	NA	NA	NA	128 (64)/124 (62)	44 (48)/42 (46)	NA	NA	326 (36)/334 (37)	30 (47)/31 (46)	NA	NA	542 (44)/572 (46)	NA	258 (35)/222 (30)

CAG, coronary angiography; NA, not available.

In each column, data are reported as invasive group/conservative group, respectively.

### 3.2. Overall outcomes

**Figure 3:**
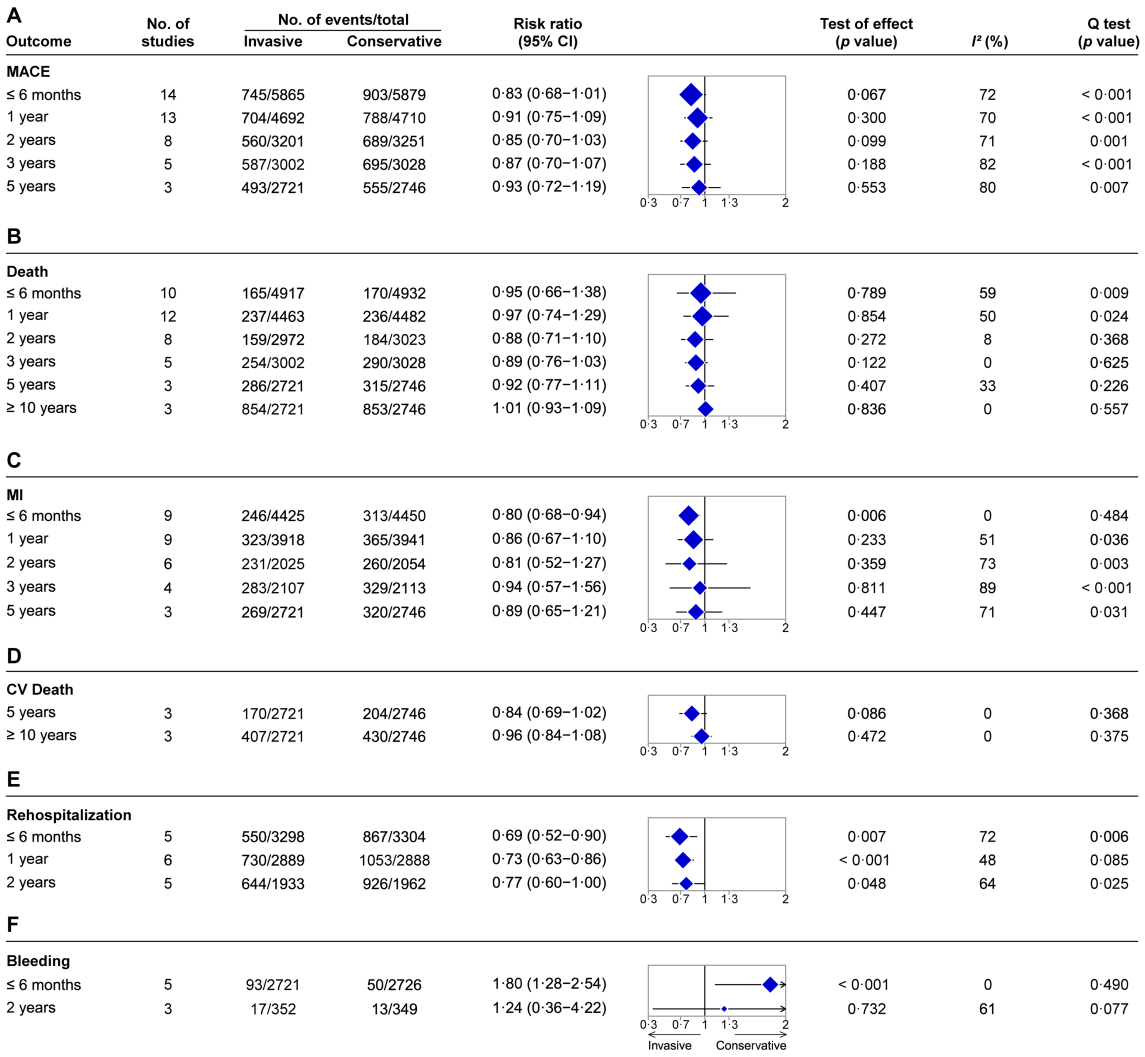
Summary of pooled estimates of invasive vs. conservative strategy on time-varied outcomes for NSTE-ACS. (**A**) MACE. (**B**) Death. (**C**) MI. (**D**) CV death. (**E**) Rehospitalization. (**F**) Bleeding. The blue diamond represents the pooled risk ratio. The horizontal bar represents 95% confidence intervals. The size of the diamond is proportional to the total number of participants included in the analyses of different follow-up time points for each outcome.

The time-varied outcomes included MACE, death, MI, CV death, rehospitalization, bleeding, in-hospital death, and in-hospital bleeding. For the MACE outcome, there were no significant differences between the two strategies at all follow-up times (Figure 3A and Supplementary Figure S2): ≤6 months (14 RCTs, *N* = 11,744): for the invasive strategy versus the conservative strategy, RR = 0.83, 95% CI = 0.68–1.01, *p *= 0.067; 1 year (13 RCTs, *N* = 9,402): RR = 0.91, 95% CI = 0.75–1.09, *p *= 0.300; 2 years (8 RCTs, *N* = 6,452): RR = 0.85, 95% CI = 0.70–1.03, *p *= 0.099; 3 years (5 RCTs, *N* = 6,030): RR = 0.87, 95% CI = 0.70–1.07, *p *= 0.188; and 5 years (3 RCTs, *N* = 5,467): RR = 0.93, 95% CI = 0.72–1.19, *p *= 0.553.

For the death outcome, no significant differences were observed between the invasive and conservative strategies at all follow-up times (Figure 3B and Supplementary Figure S3): ≤6 months (10 RCTs, *N* = 9,849): RR = 0.95, 95% CI = 0.66–1.38, *p *= 0.789; 1 year (12 RCTs, *N* = 8,945): RR = 0.97, 95% CI = 0.74–1.29, *p *= 0.854; 2 years (eight RCTs, *N* = 5,995): RR = 0.88, 95% CI = 0.71–1.10, *p *= 0.272; 3 years (five RCTs, *N* = 6,030): RR = 0.89, 95% CI = 0.76–1.03, *p *= 0.122; 5 years (three RCTs, *N* = 5,467): RR = 0.92, 95% CI = 0.77–1.11, *p *= 0.407; and ≥10 years (three RCTs, *N* = 5,467): RR = 1.01, 95% CI = 0.93–1.09, *p *= 0.836. In addition, there was also no significant difference in terms of in-hospital deaths between the two strategies (RR = 1.35, 95% CI = 0.52–3.54, *p *= 0.540, five RCTs, *N* = 841; Supplementary Figure S4).

For the MI outcome, the invasive strategy did not improve the performance of follow-up time at 1 year, 2 years, 3 years, and 5 years (Figure 3C and Supplementary Figure S5): 1 year (nine RCTs, *N* = 7,859): RR = 0.86, 95% CI = 0.67–1.10, *p *= 0.223; 2 years (six RCTs, *N* = 4,079): RR = 0.81, 95% CI = 0.52–1.27, *p *= 0.359; 3 years (four RCTs, *N* = 4,220): RR = 0.94, 95% CI = 0.57–1.56, *p *= 0.811; and 5 years (three RCTs, *N* = 5,467): RR = 0.89, 95% CI = 0.65–1.21, *p *= 0.447. However, for the follow-up time of ≤6 months, the invasive strategy proved superior to the conservative strategy in the reduction of MI events (RR = 0.80, 95% CI = 0.68–0.94, *p *= 0.006, nine RCTs, *N* = 8,875; Figure 3C and Supplementary Figure S5).

For the outcome of CV death, the invasive strategy did not modify the prognosis in follow-up times (Figure 3D and Supplementary Figure S6): 5 years (three RCTs, *N* = 5,467): RR = 0.84, 95% CI = 0.69–1.02, *p *= 0.086, and ≥10 years (three RCTs, *N* = 5,467): RR = 0.96, 95% CI = 0.84–1.08, *p *= 0.472. The results for CV deaths for follow-up times at ≤6 months, 1 year, 2 years, and 3 years were not provided because of data insufficiency or unavailability.

For rehospitalization, the invasive strategy reduced rates in follow-up times compared with the conservative strategy (Figure 3E and Supplementary Figure S7): ≤6 months (five RCTs, *N* = 6,602): RR = 0.69, 95% CI = 0.52–0.90, *p *= 0.007; 1 year (six RCTs, *N* = 5,777): RR = 0.73, 95% CI = 0.63–0.86, *p *< 0.001; and 2 years (five RCTs, *N* = 3,895): RR = 0.77, 95% CI = 0.60–1.00, *p *= 0.048. The rehospitalization rate for other follow-up times was not provided because of data unavailability.

However, an increased risk of bleeding complications was observed for the invasive strategy compared with the conservative strategy during the follow-up time of ≤6 months (RR = 1.80, 95% CI = 1.28–2.54, *p *< 0.001, five RCTs, *N* = 5,447; Figure 3F and Supplementary Figure S8). Similarly, the risk of in-hospital bleeding was also increased for the invasive strategy compared with the conservative strategy (RR = 2.17, 95% CI = 1.52–3.10, *p *< 0.001, three RCTs, *N* = 3,323; Supplementary Figure S9). No significant difference in bleeding for the 2-year follow-up time was observed for the two strategies (RR = 1.24, 95% CI = 0.36–4.22, *p *= 0.732, three RCTs, *N* = 701; Figure 3F and Supplementary Figure S8).

Considering the possible hazards of performing late follow-ups at 3, 5, and 10 years, we conducted a meta-analysis for MACE, death, MI, rehospitalization, and bleeding focused on 30 days, 6 months, 1 year, and 2 years. Compared with the conservative strategy, the invasive strategy improved the prognosis for MI at 30 days (RR = 0.67, 95% CI = 0.46–0.96, *p *= 0.03, six RCTs, *N* = 4,545; Supplementary Figure S10C) and rehospitalization at 1 year (RR = 0.73, 95% CI = 0.63–0.86, *p *< 0.001, six RCTs, *N* = 5,777; Supplementary Figure S10D) and 2 years (RR = 0.77, 95% CI = 0.60–1.00, *p *= 0.05, five RCTs, *N* = 3,895; Supplementary Figure S10D). The risk of bleeding was increased for the invasive strategy at 6 months (RR = 1.84, 95% CI = 1.18–2.87, *p *= 0.007, four RCTs, *N* = 5,261; Supplementary Figure S10E). No significant differences for MACE and death were observed for the two strategies.

### 3.3. Subgroup analyses according to high-risk features present

**Figure 4:**
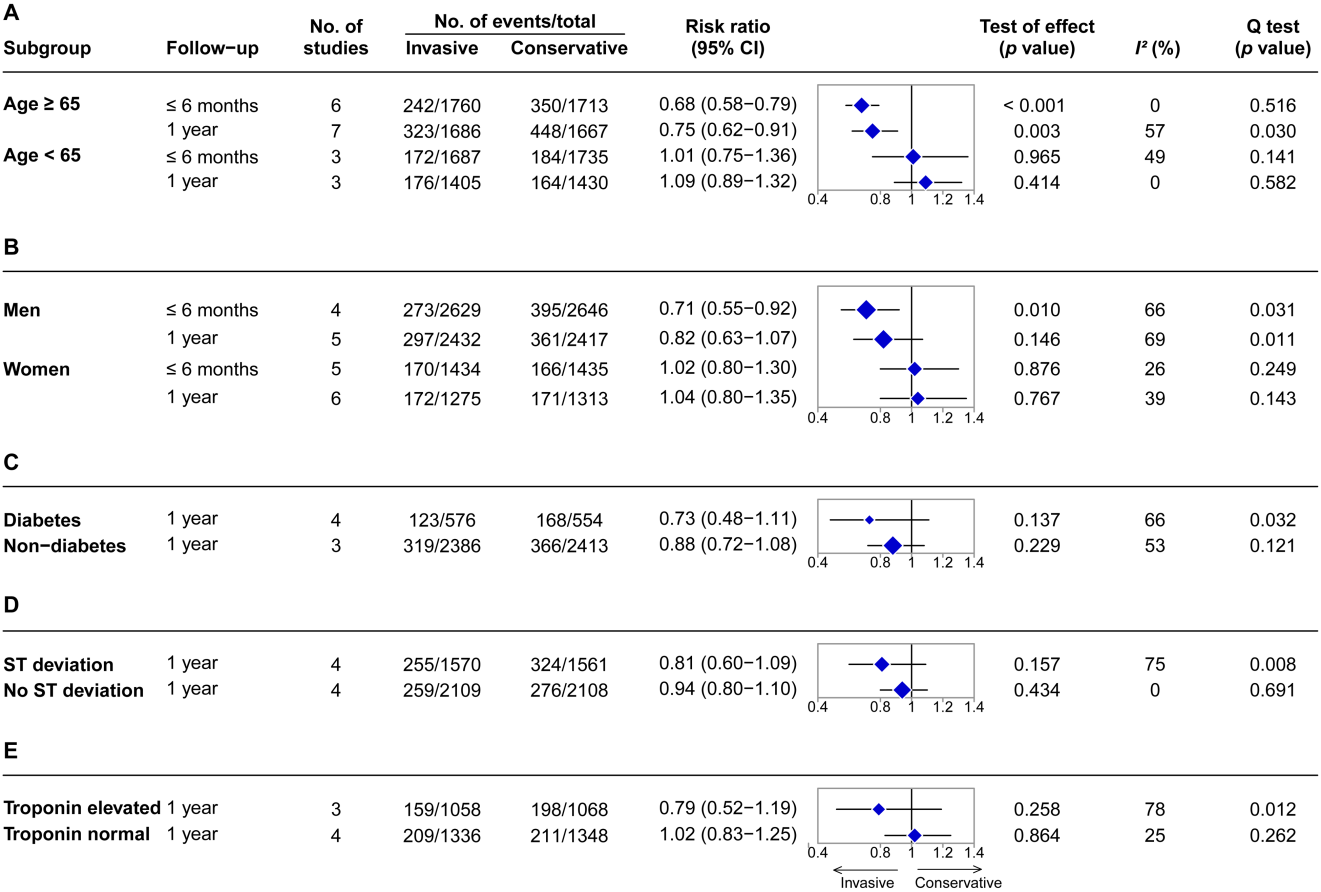
Summary effects of invasive versus conservative strategy on major adverse cardiovascular events for NSTE-ACS in subgroups stratified by high-risk features. (**A**) Stratified by age. (**B**) Stratified by gender. (**C**) Stratified by diabetes. (**D**) Stratified by ST-segment status. (**E**) Stratified by troponin level. The blue diamond represents the pooled risk ratio. The horizontal bar represents 95% confidence intervals. The size of the diamond is proportional to the total number of participants included in the analyses.

Age is a high-risk factor in adverse outcomes of NSTE-ACS. For the subgroup of patients aged ≥65 years (Figure 4A and Supplementary Figure S11A), the invasive strategy decreased MACE risk during the follow-up time of ≤6 months (RR = 0.68, 95% CI = 0.58–0.78, *p *< 0.001, six RCTs, *N* = 3,473) and 1 year (RR = 0.75, 95% CI = 0.62–0.91, *p *= 0.003, seven RCTs, *N* = 3,353). For patients aged <65 years (Figure 4A and Supplementary Figure S11B), there were no significant differences between the two strategies in MACE for ≤6 months (RR = 1.01, 95% CI = 0.75–1.36, *p *= 0.965, three RCTs, *N* = 3,422) and 1 year (RR = 1.09, 95% CI = 0.89–1.32; *p *= 0.414, three RCTs, *N* = 2,835) of follow-up.

Sub-analysis in men (Figure 4B and Supplementary Figure S12A) demonstrated a bit of benefit of the invasive strategy in MACE for the follow-up time of ≤6 months (RR = 0.71, 95% CI = 0.55–0.92, *p *= 0.010, four RCTs, *N* = 5,275), but not at 1 year (RR = 0.82, 95% CI = 0.63–1.07, *p *= 0.146, five RCTs, *N* = 4,849). There were no significant differences between the two strategies for women (Figure 4B and Supplementary Figure S12B) with respect to MACE for ≤6 months (RR = 1.02, 95% CI = 0.80–1.30, *p *= 0.876, five RCTs, *N* = 2,869) and 1 year (RR = 1.04, 95% CI = 0.80–1.35, *p *= 0.767, six RCTs, *N* = 2,588) of follow-up.

When stratified by diabetes status, no significant difference in MACE was observed between the two strategies for the subgroup of patients with diabetes at the 1-year follow-up (RR = 0.73, 95% CI = 0.48–1.11, *p *= 0.137, four RCTs, *N* = 1,130; Figure 4C and Supplementary Figure S13A). For patients without diabetes, there was also no significant difference in MACE between the two strategies at 1 year (RR = 0.88, 95% CI = 0.72–1.08, *p *= 0.229, three RCTs, *N* = 4,799; Figure 4C and Supplementary Figure S13B).

ST-segment deviation from baseline on the admission electrocardiogram, suggestive of myocardial ischemia, is a high-risk predictor of possible adverse events. In the subgroup analysis of patients with ST-segment deviation, the invasive strategy did not show benefits in MACE over the conservative strategy at 1 year (RR = 0.81, 95% CI = 0.60–1.09, *p *= 0.157, four RCTs, *N* = 3,131; Figure 4D and Supplementary Figure S14A). Similar results were also observed in the group with no ST-segment deviation (RR = 0.94, 95% CI = 0.80–1.10, *p *= 0.434, four RCTs, *N* = 4,217; Figure 4D and Supplementary Figure S14B).

Cardiac troponins are specific and sensitive biomarkers of myocardial injury. When stratified according to troponin levels, no significant differences in MACE between the two strategies were observed in both the subgroup of patients with elevated troponin levels (RR = 0.79, 95% CI = 0.52–1.19, *p *= 0.258, three RCTs, *N* = 2,126; Figure 4E and Supplementary Figure S15A) and the subgroup of patients with normal troponin levels (RR = 1.02, 95% CI = 0.83–1.25, *p *= 0.864, four RCTs, *N* = 2,684; Figure 4E and Supplementary Figure S15B) at 1 year.

### 3.4. Sensitivity analysis

The sensitivity analysis with the “leave-one-out approach” showed that ICTUS (25–28) and FRISC-II (19) significantly affected the RRs of MI and rehospitalization. OASIS 5 (5), ICTUS (25–28), and VANQWISH (32) significantly affected MACE. Excluding FRISC-II (19) and OASIS 5 (5) may affect the outcome of bleeding. No study was found to affect the outcomes of all-cause and CV deaths (Supplementary Figures S16–S23).

The meta-regression results showed a significant correlation between the percentage of previous PCI and MACE within 6 months and 1 year. There was a significant interaction between the percentage of in-hospital coronary angiography and the outcomes of MACE at 2 years, death at 1 year, and MI at 2 years. The proportion of males was a significant covariate in the outcome of bleeding at 2 years. We found no significant correlation between the covariates and rehospitalization (Supplementary Table S4). Publication bias was not detected for all outcomes except in-hospital bleeding (Supplementary Figures S24–S31).

## 4. Discussion

This meta-analysis comprehensively evaluates the time-varied outcomes of the invasive and conservative treatment strategies for patients with NSTE-ACS. Our findings show that the invasive strategy did not provide appreciable benefits for NSTE-ACS in terms of MACE, death, and CV deaths at all follow-up times compared with the conservative strategy. Although the risk of MI was reduced within 6 months for the invasive strategy, no significant differences were found in the other follow-up times between the two strategies. The invasive strategy reduced the rehospitalization rate but increased the risk of in-hospital bleeding and bleeding within 6 months compared with the conservative strategy. It should be noted that the included RCTs in assessing bleeding were very few or old. New studies are needed to determine the results. In the subgroup analyses, the invasive strategy decreased the MACE risk for patients aged ≥65 years, but not for those aged <65 years, and showed benefit for men, but not for women. In other subgroups stratified according to diabetes, ST-segment deviation, and baseline troponin levels, no significant differences were observed between the two strategies.

### 4.1. Previous meta-analyses

To date, 14 meta-analyses have sought to compare the invasive and conservative strategies associated with CV benefits for patients with NSTE-ACS (20, 34–38, 52–59). Among these, six studies (20, 34–38) reported the overall outcomes, five (52–55, 58) aimed at older patients, two (56, 59) assessed gender differences, and one (57) focused on diabetic patients. Supplementary Table S5 provides an overview of the previous meta-analyses. However, the conclusions from the previous studies, although highly debatable, largely support the use of the invasive treatment strategy. For example, Fox et al. (36) found that an invasive strategy could reduce the long-term rates of CV deaths or MI based on the collaborative analysis of FRISC-II, ICTUS, and RITA 3 and a 5-year follow-up time. In contrast, the findings of Fanning et al. (20) supported a conservative strategy. The differences in sample sizes, follow-up times, and endpoints might account for these contentious findings. Therefore, our work comprised a large sample size, multiple follow-up time points, and comprehensive outcomes to evaluate the time-varied outcomes of the two strategies systematically. First, the sample size constitutes a key confounding factor. Previous meta-analyses generally employed less than 10 RCTs and 10,000 participants (Supplementary Table S5). Our work is the largest study to have recruited 17 RCTs and 12,331 participants between 1989 and 2017. Second, the follow-up time is another important determinant of the validity or findings of any clinic-oriented study. Most previous meta-analyses focused on a specific time point (36, 56–59) or covered a long time window (34, 37, 52–54). Hoenig et al. (35) and Fanning et al. (20) divided the follow-up times into early (≤4 months), intermediate (6–12 months), and late (2–5 years) terms. This work comprehensively evaluated six follow-up times (≤6 months, 1 year, 2 years, 3 years, 5 years, and ≥10 years), which allowed us to compare the outcomes between the two strategies systematically. Third, other factors, such as endpoints in clinical trials, need to be considered. Elgendy et al. (34) only considered death, while Ma et al. (54) used death and in-hospital bleeding. In this work, a total of eight time-varied outcomes including MACE, death, MI, rehospitalization, CV death, bleeding, in-hospital death, and in-hospital bleeding were covered. Our findings strongly suggest that no significant differences between the two strategies were observed in almost all of the overall outcomes and subgroup analyses.

### 4.2. Subgroups by high-risk features

Older adults have a higher incidence, prevalence, and adverse outcomes of NSTE-ACS (60, 61). Patients older than 65 years were well represented in our meta-analysis (35.0%, 4,315 of 12,331 patients). Our results suggest that, for patients older than 65 years, the invasive approach was superior to the conservative strategy in reducing MACE for the follow-up times of ≤6 months and 1 year. Our findings support the ACC/AHA guidelines that recommend using only the invasive strategy in older patients with NSTE-ACS (62). Long-term outcomes were not evaluated because of current data insufficiency. For the gender-based subgroup analysis, our findings and other reports (56) suggest that the invasive strategy offers no benefit to women in reducing MACE. Conversely, there was evidence of short-term benefits to men at the follow-up time of ≤6 months. As reported in two previous meta-analyses, the benefits of the invasive strategy to men were also observed (56, 59). Diabetes is linked to higher prevalence and adverse outcomes of NSTE-ACS (63–65). This work indicates that patients with diabetes cannot benefit from the invasive strategy in reducing MACE rates. Our findings provide new evidence supporting the updated ESC and ACC/AHA guidelines (3, 62). Troponin assays are the preferred test in evaluating for NSTE-ACS (66, 67). Most patients with elevated troponin levels are considered high risks and recommended an invasive approach (3, 68). Surprisingly, we found no significant difference in MACE between the two strategies for patients with elevated troponins at the 1-year follow-up. This result is inconsistent with the latest guidelines (3, 62), most likely because only three studies (18, 24, 28) were included in the analysis and they were all published before 2012. Novel clinical trials targeted specifically at cohorts with elevated troponins are urgently needed.

### 4.3. Subgroups by MI definition

MI and its classification were refined by the Myocardial Infarction Consensus Document in 2007 and applied worldwide (42). Therefore, we conducted a subgroup analysis stratified by the enrollment year of 2007. For most outcomes, there were no significant differences between the two strategies in subgroup analysis of years greater than or before 2007 (Supplementary Figures S32, S33). However, significantly lower MACE within 6 months was observed in the year greater than 2007 (Supplementary Figure S32), which differed from the overall results.

### 4.4. Sub-analysis by sample size

The sample sizes of the included RCTs ranged from 52 to 2,220, which may impact the conclusions. We performed a sub-analysis excluding the small studies (5, 10, 13, 21–23) (with less than 200 patients). It is worth noting that the results were consistent with our conclusions (Supplementary Figure S34). The invasive strategy did not provide appreciable benefits in terms of MACE, death, and CV deaths but did reduce the rehospitalization rate and increased the risk of bleeding compared with the conservative strategy.

### 4.5. Study strengths

This work provides the most comprehensive evaluation of time-varied outcomes for invasive and conservative strategies in patients with NSTE-ACS. We bring strong evidence that the invasive strategy did not improve the prognosis compared with the conservative strategy, probably due to procedure-related MI and bleeding complications (20). Another aspect of our findings reflects the value of conservative treatment. In recent years, significant progress has been made in the interventional and medical management of coronary heart disease. Critical improvements such as radial access and modern drug-eluting stents have been achieved in surgical methods, while important progress has also been taken in medical treatment. Proprotein convertase subtilisin/kexin 9 (PCSK9) inhibitors [evolocumab (69) and alirocumab (70, 71)] are very effective at reducing low-density lipoprotein cholesterol (LDL-C), even in individuals at the highest risk with high LDL-C levels, and provide maximal clinical benefit (72, 73). Ticagrelor, a novel P2Y_12_ receptor inhibitor, is widely utilized due to its rapid onset and offset of action and strong antiplatelet effect. It is widely used in clinical practice as part of dual antiplatelet therapy with aspirin (74, 75). As drugs develop, we point out that the conservative strategy may be comparable to interventional therapy through optimal medical therapy, meticulous care, and close monitoring for NSTE-ACS, even for those at high risk.

### 4.6. Limitations

First, individual patient data were not available, leading to insufficiency in the subgroup analysis stratified by high-risk features, especially in long-term follow-up times. Second, the enrollment time of the 17 RCTs span almost 30 years from 1989 (29, 30) to 2017 (21). Over this timeframe, major achievements in interventional and medical management have been made (e.g., radial access, drug-eluting stents, and discontinuation of the routine use of glycoprotein IIb/IIIa inhibitors). Therapeutic outcomes may differ based on modern treatments. Third, the sample sizes ranged from 52 to 2,220 and may have contributed to high heterogeneity. Fourth, our available evidence is based on relatively old RCTs. Over the years, critical improvements have been made in surgical and medical treatment of coronary heart disease. Further studies are warranted to confirm our findings.

## 5. Conclusions

An invasive strategy is superior to a conservative strategy in reducing early events for MI and rehospitalizations, but the invasive strategy did not improve the prognosis in long-term outcomes for patients with NSTE-ACS. Further studies based on modern diagnostic and therapeutic techniques are warranted for the efficacy of the two strategies on all patients with NSTE-ACS, especially on those presenting with high-risk features.

## Data Availability

The original contributions presented in the study are included in the article/Supplementary Material, further inquiries can be directed to the corresponding authors.
