# Supplementary material for: Comprehensive evaluation of time-varied outcomes for invasive and conservative strategies in patients with NSTE-ACS: a meta-analysis of randomized controlled trials
Source: Front Cardiovasc Med. 2023 Sep 8;10:1197451. doi: 10.3389/fcvm.2023.1197451 (PMC10516546; doi:10.3389/fcvm.2023.1197451)

Supplementary appendix

Supplement to:

Comprehensive evaluation of time-varied outcomes for invasive and conservative strategies in patients with NSTE-ACS: a meta-analysis of randomized controlled trials

Yi-Jing Zhao, Yangyang Sun, Fan Wang, Yuan-Yuan Cai, Raphael N. Alolga, Lian-Wen Qi, Pingxi Xiao

Contents

Table S1. PRISMA Checklist 4

Figure S1. Trial quality and risk of bias 8

Table S2. Characteristics of randomized clinical trials included in the analysis 9

Table S3. Outcome definitions of included studies 12

Figure S2. Forest plot of MACE 14

Figure S3. Forest plot of death 15

Figure S4. Forest plot of in-hospital death 16

Figure S5. Forest plot of MI 17

Figure S6. Forest plot of CV death 18

Figure S7. Forest plot of rehospitalization 19

Figure S8. Forest plot of bleeding 20

Figure S9. Forest plot of in-hospital bleeding 21

Figure S10. Forest plots of outcomes at 30 days, 6 months, 1 year and 2 years. (A) MACE, (B) Death, (C) MI, (D) Rehospitalization, (E) Bleeding. 22

Figure S11. Forest plots of MACE stratified by age. (A) Age ≥65 years, and (B) Age <65 years. 23

Figure S12. Forest plots of MACE stratified by gender. (A) Men, and (B) Women. 24

Figure S13. Forest plots of MACE stratified by diabetes. (A) Diabetes, and (B) Non-diabetes. 25

Figure S14. Forest plots of MACE stratified by ST-segment status. (A) ST-segment deviation, and (B) No ST-segment deviation. 26

Figure S15. Forest plots of MACE stratified by troponin levels. (A) Elevated troponin levels, and (B) Normal troponin levels. 27

Figure S16. Sensitivity analysis of MACE. (A) Follow-up ≤ 6 months, (B) Follow-up at 1 year, (C) Follow-up at 2 years, (D) Follow-up at 3 years, and (E) Follow-up at 5 years. 28

Figure S17. Sensitivity analysis of death. (A) Follow-up ≤ 6 months, (B) Follow-up at 1 year, (C) Follow-up at 2 years, (D) Follow-up at 3 years, (E) Follow-up at 5 years, and (F) Follow-up ≥ 10 years. 29

Figure S18. Sensitivity analysis of MI. (A) Follow-up ≤ 6 months, (B) Follow-up at 1 year, (C) Follow-up at 2 years, (D) Follow-up at 3 years, and (E) Follow-up at 5 years. 30

Figure S19. Sensitivity analysis of CV death. (A) Follow-up at 5 years, and (B) Follow-up ≥ 10 years. 31

Figure S20. Sensitivity analysis of rehospitalization. (A) Follow-up ≤ 6 months, (B) Follow-up at 1 year, and (C) Follow-up at 2 years. 32

Figure S21. Sensitivity analysis of bleeding. (A) Follow-up ≤ 6 months, and (B) Follow-up at 2 years. . 33

Figure S22. Sensitivity analysis of in-hospital death 34

Figure S23. Sensitivity analysis of in-hospital bleeding 35

Table S4. Meta-regression results 36

Figure S24. Funnel plots of MACE. (A) Follow-up ≤ 6 months, (B) Follow-up at 1 year, (C) Follow-up at 2 years, (D) Follow-up at 3 years, and (E) Follow-up at 5 years. 43

Figure S25. Funnel plots of death. (A) Follow-up ≤ 6 months, (B) Follow-up at 1 year, (C) Follow-up at 2 years, (D) Follow-up at 3 years, (E) Follow-up at 5 years, and (E) Follow-up ≥ 10 years. 44

Figure S26. Funnel plots of MI. (A) Follow-up ≤ 6 months, (B) Follow-up at 1 year, (C) Follow-up at 2 years, (D) Follow-up at 3 years, and (E) Follow-up at 5 years. 45

Figure S27. Funnel plots of CV death. (A) Follow-up at 5 years, and (B) Follow-up ≥ 10 years. 46

Figure S28. Funnel plots of rehospitalization. (A) Follow-up ≤ 6 months, (B) Follow-up at 1 year, and (C) Follow-up at 2 years. 47

Figure S29. Funnel plots of bleeding. (A) Follow-up ≤ 6 months, and (B) Follow-up at 2 years 48

Figure S30. Funnel plots of in-hospital death. 49

Figure S31. Funnel plots of in-hospital bleeding. 50

Table S5. Overview on previous meta-analyses investigating an invasive versus conservative strategy in patients with NSTE-ACS 51

Figure S32. Forest plots of outcomes in the subgroup of the enrollment year greater than 2007. (A) MACE, and (B) Death. 55

Figure S33. Forest plots of outcomes in the subgroup of the enrollment year less than 2007. (A) MACE, (B) Death, (C) MI, (D) CV death, (E) Rehospitalization, and (F) Bleeding. 56

Figure S34. Forest plots of outcomes in the sub analysis excluding the small studies (less than 200 patients). (A) MACE, (B) Death, (C) MI, (D) CV death, (E) Rehospitalization, and (F) Bleeding. 57

Table S1. PRISMA Checklist

Section and Topic	Item #	Checklist item	Location where item is reported
**TITLE**			
Title	1	Identify the report as a systematic review.	Page 1
**ABSTRACT**			
Abstract	2	See the PRISMA 2020 for Abstracts checklist.	Page 2-3
**INTRODUCTION**			
Rationale	3	Describe the rationale for the review in the context of existing knowledge.	Page 5
Objectives	4	Provide an explicit statement of the objective(s) or question(s) the review addresses.	Page 5
**METHODS**			
Eligibility criteria	5	Specify the inclusion and exclusion criteria for the review and how studies were grouped for the syntheses.	Page 7
Information sources	6	Specify all databases, registers, websites, organisations, reference lists and other sources searched or consulted to identify studies. Specify the date when each source was last searched or consulted.	Page 6
Search strategy	7	Present the full search strategies for all databases, registers and websites, including any filters and limits used.	Page 6
Selection process	8	Specify the methods used to decide whether a study met the inclusion criteria of the review, including how many reviewers screened each record and each report retrieved, whether they worked independently, and if applicable, details of automation tools used in the process.	Page 7
Data collection process	9	Specify the methods used to collect data from reports, including how many reviewers collected data from each report, whether they worked independently, any processes for obtaining or confirming data from study investigators, and if applicable, details of automation tools used in the process.	Page 7
Data items	10a	List and define all outcomes for which data were sought. Specify whether all results that were compatible with each outcome domain in each study were sought (e.g. for all measures, time points, analyses), and if not, the methods used to decide which results to collect.	Page 7; Table S3
	10b	List and define all other variables for which data were sought (e.g. participant and intervention characteristics, funding sources). Describe any assumptions made about any missing or unclear information.	Page 7
Study risk of bias assessment	11	Specify the methods used to assess risk of bias in the included studies, including details of the tool(s) used, how many reviewers assessed each study and whether they worked independently, and if applicable, details of automation tools used in the process.	Page 8
Effect measures	12	Specify for each outcome the effect measure(s) (e.g. risk ratio, mean difference) used in the synthesis or presentation of results.	Page 8
Synthesis methods	13a	Describe the processes used to decide which studies were eligible for each synthesis (e.g. tabulating the study intervention characteristics and comparing	Page 7;

Section and Topic	Item #	Checklist item	Location where item is reported
		against the planned groups for each synthesis (item #5)).	Table S2
	13b	Describe any methods required to prepare the data for presentation or synthesis, such as handling of missing summary statistics, or data conversions.	Page7
	13c	Describe any methods used to tabulate or visually display results of individual studies and syntheses.	Page 7
	13d	Describe any methods used to synthesize results and provide a rationale for the choice(s). If meta-analysis was performed, describe the model(s), method(s) to identify the presence and extent of statistical heterogeneity, and software package(s) used.	Page 8
	13e	Describe any methods used to explore possible causes of heterogeneity among study results (e.g. subgroup analysis, meta-regression).	Page 8
	13f	Describe any sensitivity analyses conducted to assess robustness of the synthesized results.	Page 8
Reporting bias assessment	14	Describe any methods used to assess risk of bias due to missing results in a synthesis (arising from reporting biases).	Page 8
Certainty assessment	15	Describe any methods used to assess certainty (or confidence) in the body of evidence for an outcome.	Page 8
**RESULTS**			
Study selection	16a	Describe the results of the search and selection process, from the number of records identified in the search to the number of studies included in the review, ideally using a flow diagram.	Page 9; Figure 1
	16b	Cite studies that might appear to meet the inclusion criteria, but which were excluded, and explain why they were excluded.	Page 9
Study characteristics	17	Cite each included study and present its characteristics.	Page 9; Table 1; Table S2
Risk of bias in studies	18	Present assessments of risk of bias for each included study.	Page 9; Figure S2
Results of individual studies	19	For all outcomes, present, for each study: (a) summary statistics for each group (where appropriate) and (b) an effect estimate and its precision (e.g. confidence/credible interval), ideally using structured tables or plots.	Page 10-13 Figure S2-S14
Results of syntheses	20a	For each synthesis, briefly summarise the characteristics and risk of bias among contributing studies.	Page 12
	20b	Present results of all statistical syntheses conducted. If meta-analysis was done, present for each the summary estimate and its precision (e.g.	Page 10-13;

Section and Topic	Item #	Checklist item	Location where item is reported
		confidence/credible interval) and measures of statistical heterogeneity. If comparing groups, describe the direction of the effect.	Figure 3; Figure 4
	20c	Present results of all investigations of possible causes of heterogeneity among study results.	Page 13-14; Table S4
	20d	Present results of all sensitivity analyses conducted to assess the robustness of the synthesized results.	Page 13; Figure S15-S22
Reporting biases	21	Present assessments of risk of bias due to missing results (arising from reporting biases) for each synthesis assessed.	Page 14; Figure S23-S30
Certainty of evidence	22	Present assessments of certainty (or confidence) in the body of evidence for each outcome assessed.	Figure S2
**DISCUSSION**			
Discussion	23a	Provide a general interpretation of the results in the context of other evidence.	Page 14
	23b	Discuss any limitations of the evidence included in the review.	Page 18
	23c	Discuss any limitations of the review processes used.	Page 18
	23d	Discuss implications of the results for practice, policy, and future research.	Page 17
**OTHER INFORMATION**			
Registration and protocol	24a	Provide registration information for the review, including register name and registration number, or state that the review was not registered.	Page 6
	24b	Indicate where the review protocol can be accessed, or state that a protocol was not prepared.	Page 6
	24c	Describe and explain any amendments to information provided at registration or in the protocol.	None
Support	25	Describe sources of financial or non-financial support for the review, and the role of the funders or sponsors in the review.	Page 1
Competing interests	26	Declare any competing interests of review authors.	Page 18
Availability of data, code and other	27	Report which of the following are publicly available and where they can be found: template data collection forms; data extracted from included studies; data used for all analyses; analytic code; any other materials used in the review.	Page 18

Section and Topic	Item #	Checklist item	Location where item is reported
materials			

Figure S1. Trial quality and risk of bias

	Intention to treat principle	Random sequence generation	Random sequence generation	Blinding of participants and personnel	Blinding of outcome assessment	Incomplete outcome data	Selective reporting
**Hirlekar *et al***	Yes	Low	Low	High	Unclear	Low	Low
**Lee *et al***	Yes	Low	Low	High	Unclear	Low	Low
**MOSCA**	Yes	Low	Low	High	Low	Low	Low
**After Eighty**	Yes	Low	Low	High	Unclear	Low	Low
**Dimitrov *et al***	Yes	Unclear	Low	High	Unclear	Low	Unclear
**Italian Elderly ACS**	Yes	Low	Low	High	Unclear	Low	Low
**LIPSIA-NSTEMI**	Yes	Unclear	Low	High	Low	Low	Low
**OASIS 5**	Yes	Low	Low	High	Low	Low	Low
**ICTUS**	Yes	Unclear	Low	High	Low	Low	Low
**Eisenberg *et al***	Yes	Low	Unclear	High	Unclear	Low	Low
**RITA 3**	NA	Low	Low	High	Low	Low	Low
**VINO**	Yes	Unclear	Unclear	High	Unclear	Low	Unclear
**TACTICS-TIMI 18**	NA	Low	Low	High	Unclear	Low	Low
**TRUCS**	NA	Low	Low	Low	Low	Low	Low
**FRISC-II**	NA	Unclear	Unclear	High	Unclear	Low	Low
**VANQWISH**	Yes	Low	Low	High	Unclear	Low	Low
**TIMI IIIB**	Yes	Low	Unclear	High	Low	Low	Low

NA, not available

Table S2. Characteristics of randomized clinical trials included in the analysis

Study	Disease type	Country	Trial centers	Enroll-ment	Inclusion criteria	Exclusion criteria	Invasive strategy	Conservative strategy
**Hirlekar *et al***	NSTE-ACS	Sweden	3	2009-2017	(1) Age ≥80 years; (2) NSTE-ACS with ischemic symptoms lasting over 10 min in the previous 72 h; and (3) ST-segment depression 1 mm and/or elevated, troponin T or CK-MB.	(1) PCI within 30 days; (2) ongoing active internal bleeding; (3) ST-segment elevation of 1 mm in two contiguous leads on ECG; (4) enrolled in another study not completed; (5) allergy to aspirin or P2Y12 antagonists; (6) severe dementia; (7) expected limited 1-year survival due to other diseases; or (8) unwillingness to participate or expected problems with compliance.	Invasive strategy included CAG and, if appropriate, revascularization with PCI or CABG, and OMT.	Conservative strategy included OMT without CAG. CAG performed only after refractory chest pain, hemodynamic instability, heart failure, or life-threatening cardiac arrhythmias.
**Lee *et al***	NSTE-ACS	United Kingdom	16	2012-2013	(1) Unstable angina; (2) non-ST segment elevation MI; (3) stabilized symptoms without recurrent chest pain or intravenous therapy for 12 hours; and (4) prior CABG.	(1) Refractory ischemia; (2) cardiogenic shock; (3) lack of informed consent; or (4) unsuitable for invasive management.	Invasive strategy included native coronary and bypass graft angiography and coronary and graft revascularization with PCI and CABG, as clinically appropriate.	Conservative strategy was medical therapy, and could be referred for invasive management if prespecified criteria occurred post-randomization.
**MOSCA**	NSTEMI	Spain	4	2012-2014	(1) Age ≥70 years; (2) NSTEMI with chest pain, non-ST ECG and troponin elevation; and (3) at least two of the following: renal failure, neurological disease with a residual deficit, peripheral artery disease, dementia, chronic pulmonary disease or anemia.	(1) Dynamic ST-segment changes suggestive of ischemia; (2) non-revascularizable coronary artery disease; (3) concomitant heart disease not ischemic; or (4) life expectancy <1 year.	Invasive strategy group received routine cardiac catheterization within 72 h of admission.	Conservative patients underwent only medical treatment, and cardiac catheterization was allowed in the case of recurrent ischemia or heart failure after admission, or in the case of a positive predischarge non-invasive stress test.
**After Eighty**	NSTE-ACS	Norway	58	2010-2014	(1) Age ≥80 years; (2) NSTE-ACS with chest pain >10 minutes; (3) with or without ST depression; and (4) normal or elevated levels of troponin T or I.	(1) Age <80 years; (2) ST-segment elevation in ECG; (3) clinical unstable with ongoing chest pain or other ischaemic symptoms/signs; (4) cardiogenic shock; (5) short life expectancy due to extra cardiac reason; or (6) anamnestic indications for significant mental disorder.	Invasive strategy included early CAG with immediate assessment for adhoc PCI, CABG, or OMT.	Conservative strategy was OMT alone.
**Dimitrov *et al***	NSTE-ACS	Bulgaria	6	2007-2011	(1) Typical chest pain for UA with the last pain episode occurring within 24 hours prior to admission; (2) ECG ST-segment abnormalities; and (3) cardiac troponin I value above the upper limit of the norm.	Unwillingness or inability to sign informed consent for coronary arteriography or PCI.	Invasive strategy was scheduled to undergo CAG and PCI within 24 hours after admission.	Patients assigned to the conservative strategy were medically stabilized at first, with CAG required only in case of angina recurrence and/or evidence of inducible myocardial ischemia.
**Italian Elderly ACS**	NSTE-ACS	Italian	23	2008-2010	(1) Age ≥75 years; (2) NSTE-ACS with cardiac ischemic symptoms at rest within 48h; (3) ischemic ECG changes; and (4) elevated levels of either troponin or CK-MB.	(1) Secondary causes of myocardial ischemia; (2) ongoing myocardial ischemia or heart failure; (3) PCI or CABG within 30 days; (4) serum creatinine 2.5 mg/dl; (5) a cerebrovascular accident within the previous month; (6) recent transfusions, gastrointestinal or genitourinary bleeding within 6 weeks; or (7) severe obstructive lung disease, alignancy, or neurological deficit.	Invasive strategy included CAG within 72 h and, when indicated, coronary revascularization by either PCI or CABG according to coronary anatomy, patient preference, and local skills.	Conservative therapy was medical therapy, and CAG during index hospital was allowed in the case of refractory ischemia, myocardial (re)infarction, heart failure of ischemic origin, or malignant ventricular arrhythmias.
**LIPSIA-NSTEMI**	NSTEMI	Germany	169	2006-2010	(1) NSTE-MI with ischemia symptoms increasing or occurred at rest within 24 hours; and (2) elevated troponin T level ≥0.1 ng/Ml.	(1) Age <18 and >90 years; (2) refractory ischaemia, haemodynamic instability, congestive heart failure; (3) major arrhythmias requiring immediate catheterization; (4) oral anticoagulation, contraindications to heparin, aspirin, clopidogrel, and glycoprotein IIb/IIIa inhibitors; (5) comorbidity with life expectancy <6 months; or (6) econdary to congestive heart failure or hypertensive crisis.	Patients assigned to the invasive strategy were scheduled to undergo CAG by a time window of 10–48 h after randomization.	Patients assigned to the conservative strategy were initially treated medically, with immediate intervention required only if the patients met prespecified criteria.
**OASIS 5**	NSTE-ACS	Multi-country (41)	42	2003-2005	(1) Age ≥60 years; (2) an elevated level of troponin or CK-MB above upper limit of normal; and (3) ECG changes indicative of ischaemia.	(1) Age <21; (2) contraindications to low-molecular-weight heparin; (3) recent haemorrhagic stroke; (4) indications for anticoagulation; (5) pregnancy; (6) co-morbid condition with life expectancy <6 months; or (7) severe renal insufficiency.	Invasive strategy was a routine CAG within 4 days of admission and, if appropriate, revascularization within 7 days of admission.	Patients randomized to the conservative strategy underwent CAG only if they experienced symptoms or signs of severe ischaemia.
**ICTUS**	NSTE-ACS	Netherlands	45	2001-2003	(1) NSTE-ACS with ischemia symptoms increasing or occurred at rest within 24 hours; (2) elevated cardiac troponin T level (≥0.03 μg per liter); (3) ischemic changes as assessed by	(1) Age <18 or >80 years; (2) ST elevation within 48 hours; (3) indication for PCI or fibrinolytic therapy; (4) hemodynamic instability or overt congestive heart failure; (5) oral anticoagulant drugs within 7 days, fibrinolytic treatment within 96 hours, PCI within 14 days; (6) contraindication to PCI or	Patients assigned to the invasive strategy were scheduled to undergo CAG within 24 to 48 hours after randomization	Patients assigned to the conservative strategy were treated medically. These patients were scheduled to undergo CAG and subsequent revascularization only if

					ECG; and (4) history of coronary artery disease.	glycoprotein IIb/IIIa inhibitors; (7) recent trauma or risk of bleeding; (8) hypertension despite treatment; or (9) weight greater than 120 kg.		they had refractory angina
**Eisenberg *et al***	NQWMI	United States /Pretoria/India/Canada	8	1999-2003	(1) Age <80 years; (2) ST elevation or depression or T wave inversion, or creatine kinase elevation 2 times the upper limit of normal and/or elevation in CK-MB, troponin T, or troponin I; and (3) treated with aspirin plus heparin and/or a glycoprotein IIb/IIIa inhibitors for a minimum of 48 hours.	(1) New pathological Q wave on ECG; (2) one or more of the following: shock, congestive heart failure, ventricular arrhythmia, recurrent or persistent chest pain or myocardial ischemia; (3) contraindications to repeat cardiac procedures; (4) primary PCI during the current admission or angiography, PCI, or CABG within 6 months; (5) contraindications or inability to undergo treadmill testing; (6) life expectancy <1 year; or (7) being unlikely to be available for follow-up stress testing.	Patients randomized to the invasive arm underwent angiography at 2 to 5 days following their NQWMI	Patients randomized to the noninvasive arm underwent stress testing (stress echocardiography or stress nuclear perfusion imaging) at 2 to 5 days following their NQWMI.
**RITA 3**	NSTE-ACS	United Kingdom	11	1997-2001	(1) Cardiac pain associated with ECG or previous arteriographic evidence of coronary artery disease; and (2) with elevated serum cardiac marker.	Obviously at high risk of death or myocardial infarction.	Patients undertook CAG within 72 h in the interventional strategy with subsequent management guided by the angiographic findings.	Patients assigned to the conservative strategy were managed with antianginal and antithrombotic medication
**VINO**	NSTEMI	Czech Republic	2	1998-2000	(1) Rest ischaemic chest pain, lasting more than 20 min within 24 h; (2) ECG evidence of acute myocardial ischaemia without ST-segment elevations; and (3) CK-MB higher than 1·5× upper limit of normal and/or positive troponin I assay.	(1) Cardiogenic shock; (2) acute LBBB or RBBB or ST segment elevations ≤2 mm in two leads; (3) Q-wave MI or intravenous thrombolysis less than 1 month; (4) CABG less than 6 months; or (5) Any concomitant disease which may have possible influence on 1 year prognosis.	Invasive strategy characterized by CAG as soon as possible followed by immediate coronary angioplasty of the culprit coronary lesion+stent implantation whenever suitable.	Conservative strategy characterized by initial medical treatment with CAG and subsequent revascularization only in the presence of recurrent myocardial ischaemia.
**TACTICS-TIMI 18**	NSTE-ACS	Multi-country	25	1997-1999	(1) Age ≥18 years; (2) angina within 24 hours; and (3) ST-segment depression of at least 0.05 mV, transient (<20 minutes) ST-segment elevation of at least 0.1 mV, T-wave inversion of at least 0.3 mV in at least two leads, elevated levels of cardiac markers, or coronary disease.	(1) Persistent ST-segment elevation; (2) secondary angina; (3) a history of PCI or CABG within 6 months; (4) factors associated with an increased risk of bleeding; (5) left bundle-branch block or paced rhythm; (6) severe congestive heart failure or cardiogenic shock; (7) serious systemic disease; (8) serum creatinine level of more than 2.5 mg per deciliter; (9) participation in another study; (10) taking warfarin or ticlopidine or clopidogrel for more than three days.	Invasive strategy was to undergo CAG between 4 and 48 hours after randomization and revascularization when appropriate on the basis of coronary anatomical findings.	Patients assigned to conservative strategy were treated medically, and were to undergo CAG and revascularization as appropriate only if they had symptoms like recurrent angina and so on.
**TRUCS**	UA	Greece	576	1997-1998	(1) Primary unstable angina and post-infarction angina; and (2) did not suffer a new myocardial infarction or death within the first 48 h post admission and subsequently developed refractory unstable angina.	Patients refusing to give informed consent.	Patients assigned to the invasive strategy underwent CAG locally, the day the diagnosis of refractory unstable angina was made.	Patients assigned to the conservative strategy were treated medically on the initial 'optimal' therapy regime with gradually increasing doses of nitrates and calcium antagonists unless contraindicated.
**FRISC-II**	NSTE-ACS	Sweden/Denmark/Norway	2	1996-1998	(1) Ischaemia symptoms increasing or occurring at rest; (2) with the last episode within 48 h before the start of dalteparin or standard heparin treatment; (3) ST depression or T-wave inversion ≥0·1 mV; or (4) raised biochemical markers.	(1) High risk of bleeding or anaemia; (2) indication for or treatment within the previous 24 h with thrombolysis; (3) angioplasty within 6 months; (4) waiting for coronary revascularization procedure; (5) other acute or severe cardiac disease; (6) renal insufficiency, hepatic insufficiency; (7) known clinically relevant osteoporosis; (8) other severe illness; (9) hypersensitivity to randomly assigned drugs; (10) suspected difficulty with cooperation; or (11) participation in another clinical trial.	The invasive strategy included CAG and, if appropriate, revascularization within seven days of hospital admission.	The conservative strategy recommended CAG in patients with refractory or recurrent symptoms, despite maximal medical treatment or severe ischemia on a predischarge symptom-limited exercise test.
**VANQWISH**	NQWMI	United States	6	1993-1995	(1) Evolving acute myocardial infarction; (2) CK-MB 1.5 times the upper limit of normal; (3) no new abnormal Q waves (or R waves) on ECGs.	Serious coexisting conditions, ischemic complications at very high risk while in the coronary care unit.	Patients assigned to the invasive strategy underwent CAG as the initial diagnostic test soon after randomization.	The conservative strategy was medical therapy with subsequent invasive management if indicated by the development of spontaneous or inducible ischemia.
**TIMI IIIB**	UA/NQWMI	United States /Canada	15	1989-1992	(1) New or presumably new ECG evidence of ischemia in at least two contiguous leads; (2) documented coronary artery disease.	(1) A treatable cause of unstable angina; (2) previous MI within the preceding 21 days; (3) CABG within 30 days, PTCA within 6 months, CABG at any time; (4) pulmonary edema; (5) systolic arterial pressure >180 mm Hg or diastolic pressure >100; (6) contraindication to thrombolytic therapy or heparin; (7) left bundle branch block; (8) coexistent severe illness; (9) a	Patients assigned to the invasive strategy underwent cardiac catheterization, left ventricular angiography, and coronary arteriography 18 to 48 hours after	Patients randomized to the conservative strategy underwent cardiac catheterization and angiography only after failure of initial therapy.

						woman of child-bearing potential; or (10) receiving oral anticoagulants.	randomization.	

NSTE-ACS, non–ST-segment elevation acute coronary syndrome; NSTE-MI, non–ST-segment elevation myocardial infarction; MI, myocardial infarction; NQWMI, non–Q-wave myocardial infarction; UA, unstable angina; CAG, coronary angiography; PCI, percutaneous coronary intervention; CABG, coronary artery bypass graft; PTCA, percutaneous transluminal coronary angioplasty; OMT, optimum medical treatment; CK-MB, creatine kinase-myocardial band isoenzyme; ECG, electrocardiographic.

Table S3. Outcome definitions of included studies

Study	Major adverse cardiovascular events	Death	Myocardial infarction	Rehospitalization	Bleeding
**Hirlekar *et al***	Combination of MI, urgent revascularization, all-cause mortality, stroke, and recurrent hospitalization for cardiac reasons.	Death from any cause.	NA	Rehospitalization for cardiac reasons as due to new onset atrial fibrillation or heart failure.	Major bleeding, defined as either intracranial bleeding, a decrease in haemoglobin level of more than 50 g/L, or bleeding requiring surgery.
**Lee *et al***	Combination of all-cause mortality, rehospitalization for refractory ischemia/angina, MI, or hospitalization for HF.	Death from any cause.	Nonfatal MI.	Rehospitalization for any reason.	Bleeding Academic Research Consortium types 2–4.
**MOSCA**	Combination of all-cause mortality, reinfarction and readmission for cardiac cause (postdischarge revascularization or heart failure).	Death from any cause.	New episode of chest pain with troponin elevation after admission, during either the index hospital stay or a new readmission and readmission for cardiac cause.	Readmission for cardiac cause.	Bleeding ≥ TIMI 2 according to the Thrombolysis in Myocardial Infarction bleeding criteria.
**After Eighty**	Combination of MI, need for urgent revascularization, stroke, and death-the first occurring event.	Death from any cause.	NA	NA	Major bleeding according to the Thrombolysis in Myocardial Infarction bleeding criteria.
**Dimitrov *et al***	Combination of frequency of occurrence of recurrent angina, re-hospitalization, coronary arteriography and intervention, development of MI, symptoms of heart failure, total mortality rate.	Death from any cause.	NA	NA	NA
**Italian Elderly ACS**	Combination of all-cause mortality, nonfatal MI, disabling stroke, and repeat hospital stay for cardiovascular causes or severe bleeding.	Death from any cause.	Nonfatal MI.	Repeat hospital stay for cardiovascular causes or severe bleeding.	Bleeding Academic Research Consortium grades 2, 3a, and 3b.
**LIPSIA-NSTEMI**	Combination of death, non-fatal infarction, refractory ischaemia, and rehospitalization for unstable angina.	NA	Nonfatal MI.	Rehospitalization for unstable angina.	Moderate bleeding occurring either spontaneously, PCI-related, or CABG-related as assessed by the GUSTO definition and the occurrence of any ischaemic stroke.
**OASIS 5**	Combination of death, MI, or stroke.	NA	NA	NA	Major bleeding, defined as clinically overt bleeding with at least one of the following criteria: fatal, symptomatic intracranial haemorrhage, intraocular haemorrhage leading to significant vision loss or decrease in haemoglobin of 30 g/L, or requiring transfusion of 2 U of blood.
**ICTUS**	Combination of death, recurrent MI, or rehospitalization for angina within one year after randomization.	Death from any cause.	Documented myocardial necrosis, occurring either spontaneously or in the setting of percutaneous intervention.	Rehospitalization for anginal symptoms.	NA
**Eisenberg *et al***	Combination of death, recurrent myocardial infarction, and unstable angina.	NA	NA	NA	NA
**RITA 3**	Combination of death, non-fatal MI, or refractory angina at 4 months; and combination of death or nonfatal MI at 1 and 5 years.	NA	Nonfatal MI.	NA	NA
**VINO**	Combination of death or non-fatal recurrent MI.	NA	Nonfatal MI.	NA	Major bleeding according to the standard TIMI definition.
**TACTICS-TIMI 18**	Combination of death, nonfatal MI, and rehospitalization for an acute coronary syndrome.	Death from any cause.	Nonfatal MI.	Rehospitalization for an acute coronary syndrome	NA
**TRUCS**	Combination of death and non-fatal MI.	NA	The occurrence of two of the three conventional criteria-typical chest pain, diagnostic electrocardiography recording (mainly new Q-wave), or a raised biochemical marker of myocardial damage.	Readmission for unstable angina.	NA
**FRISC-II**	Combination of all-cause death and MI.	Death from any cause.	The occurrence of two of the three conventional criteria-typical chest pain, diagnostic electrocardiography recording (mainly new Q-wave), or a raised biochemical marker of myocardial damage according to the following definitions.	NA	Major bleeding, defined as at least one of: leading to death; intracranial bleed; need for blood transfusion; decrease in haemoglobin of 40 g/L or more, irrespective of symptoms; and decrease in haemoglobin of more than 20 g/L associated with symptoms of bleeding.
**VANQWISH**	Combination of death or nonfatal MI.	Death from any cause.	Nonfatal MI.	NA	NA

**TIMI IIIB**	Combination of death, postrandomization nonfatal MI (or failure of initial therapy).	NA	Nonfatal MI.	Hospital admission for unstable angina.	NA

MI, myocardial infarction; PCI, percutaneous coronary intervention; CABG, coronary artery bypass graft; NA, not available.

Figure S2. Forest plot of MACE

Study and Year	PMID	Invasive	Conservative	RR (95% CI)
**Follow-up ≤ 6 months**				
Hirlekar *et al*, 2020	32586153	11/93	15/93	0·73 (0·36-1·51)
MOSCA, 2016	27423981	16/52	20/54	0·83 (0·49-1·42)
After Eighty, 2016	26794722	44/229	79/228	0·55 (0·40-0·76)
Dimitrov *et al*, 2013	24191395	1/22	19/30	0·07 (0·01-0·50)
LIPSIA-NSTEMI, 2011	22108830	53/200	49/200	1·08 (0·77-1·51)
OASIS 5, 2009	19202154	14/92	8/92	1·75 (0·77-3·97)
ICTUS, 2005	16162880	124/604	111/596	1·10 (0·88-1·39)
RITA 3, 2002	12241831	86/895	133/915	0·66 (0·51-0·85)
VINO, 2002	11792138	4/64	15/67	0·28 (0·10-0·80)
TACTICS-TIMI 18, 2001	11419424	177/1114	215/1106	0·82 (0·68-0·98)
TRUCS, 2000	11071801	4/76	11/72	0·34 (0·11-1·03)
FRISC-II, 1999	10475181	113/1222	148/1235	0·77 (0·61-0·97)
VANQWISH, 1998	9632444	45/462	23/458	1·94 (1·19-3·15)
TIMI IIIB, 1994	8149520	53/740	57/733	0·92 (0·64-1·32)
**Random effects model (I^2=72%)**		**745/5865**	**903/5879**	**0·83 (0·68-1·01)**
**Follow-up at 1 year**				
Hirlekar *et al*, 2020	32586153	31/93	34/93	0·91 (0·62-1·35)
Lee *et al*, 2019	31362541	7/31	5/29	1·31 (0·47-3·67)
MOSCA, 2016	27423981	23/52	24/54	1·00 (0·65-1·52)
After Eighty, 2016	26794722	59/229	110/228	0·53 (0·41-0·69)
Savonitto *et al*, 2012	22995877	43/154	55/159	0·81 (0·58-1·12)
OASIS 5, 2009	19202154	15/92	11/92	1·36 (0·66-2·81)
ICTUS, 2005	16162880	137/604	126/596	1·07 (0·87-1·33)
Eisenberg *et al*, 2005	15894961	6/42	7/46	0·94 (0·34-2·57)
RITA 3, 2002	12241831	68/895	76/915	0·91 (0·67-1·25)
TRUCS, 2000	11071801	6/76	12/72	0·47 (0·19-1·20)
FRISC-II, 2000	10892758	127/1222	174/1235	0·74 (0·60-0·91)
VANQWISH, 1998	9632444	102/462	65/458	1·56 (1·17-2·06)
TIMI IIIB, 1995	7594098	80/740	89/733	0·89 (0·67-1·18)
**Random effects model (I^2=70%)**		**704/4692**	**788/4710**	**0·91 (0·75-1·09)**
**Follow-up at 2 years**				
Lee *et al*, 2019	31362541	13/31	13/29	0·94 (0·52-1·67)
MOSCA, 2016	27423981	29/52	29/54	1·04 (0·73-1·47)
After Eighty, 2016	26794722	79/229	133/228	0·59 (0·48-0·73)
Dimitrov *et al*, 2013^	24191395	9/22	24/30	0·51 (0·30-0·87)
Dimitrov *et al*, 2013§	24191395	20/54	25/72	1·07 (0·67-1·71)
OASIS 5, 2009	19202154	19/92	14/92	1·36 (0·73-2·54)
ICTUS, 2007	17350451	160/604	145/596	1·09 (0·90-1·32)
RITA 3, 2005	16154018	85/895	106/915	0·82 (0·63-1·07)
FRISC-II, 2002	12475448	146/1222	200/1235	0·74 (0·61-0·90)
**Random effects model (I^2=71%)**		**560/3201**	**689/3251**	**0·85 (0·70-1·03)**
**Follow-up at 3 years**				
MOSCA, 2016	27423981	38/52	38/54	1·04 (0·82-1·32)
After Eighty, 2016	26794722	93/229	140/228	0·66 (0·55-0·80)
ICTUS, 2007	17350451	175/604	150/596	1·15 (0·96-1·39)
FRISC-II, 2006	16980115	172/1222	236/1235	0·74 (0·62-0·88)
RITA 3, 2005	16154018	109/895	131/915	0·85 (0·67-1·08)
**Random effects model (I^2=82%)**		**587/3002**	**695/3028**	**0·87 (0·70-1·07)**
**Follow-up at 5 years**				
ICTUS, 2010	20045278	134/604	107/596	1·24 (0·98-1·55)
FRISC-II, 2006	16980115	217/1222	270/1235	0·81 (0·69-0·95)
RITA 3, 2005	16154018	142/895	178/915	0·82 (0·67-1·00)
**Random effects model (I^2=80%)**		**493/2721**	**555/2746**	**0·93 (0·72-1·19)**

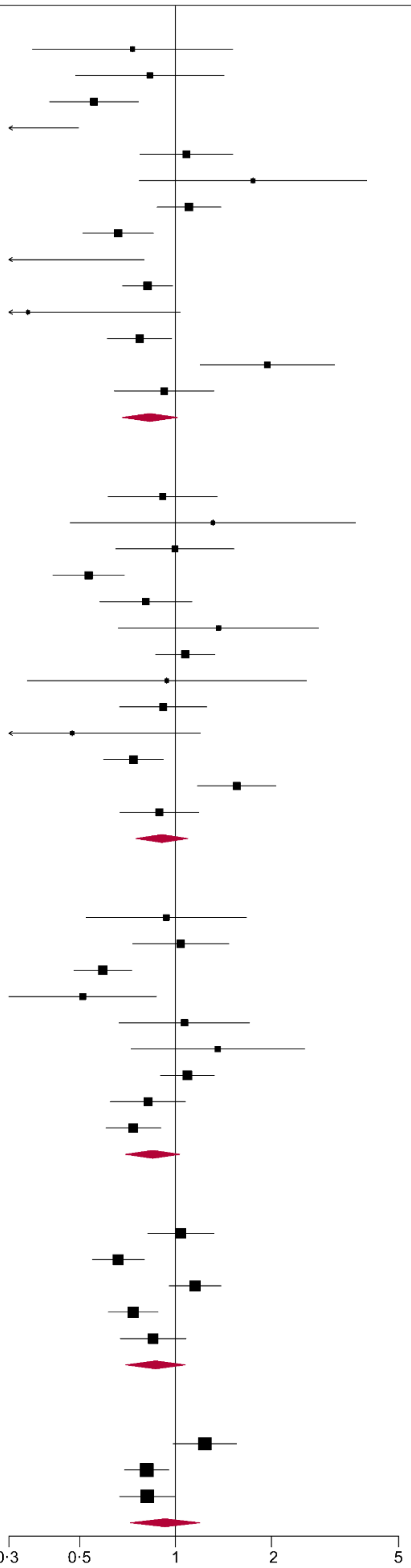

^ Diabetic group, § non-diabetic group.

Figure S3. Forest plot of death

Study and Year	PMID	Invasive	Conservative	RR (95% CI)
**Follow-up ≤ 6 months**				
MOSCA, 2016	27423981	10/52	13/54	0·80 (0·38-1·66)
LIPSIA−NSTEMI, 2011	22108830	12/200	13/200	0·92 (0·43-1·97)
OASIS 5, 2009	19202154	7/92	1/92	7·00(0·88-55·77)
RITA 3, 2002	12241831	26/895	23/915	1·16 (0·66-2·01)
VINO, 2002	11792138	2/64	9/67	0·23 (0·05-1·04)
TACTICS−TIMI 18, 2001	11419424	37/1114	39/1106	0·94 (0·61-1·47)
TRUCS, 2000	11071801	2/76	8/72	0·24 (0·05-1·08)
FRISC-II, 1999	10475181	23/1222	36/1235	0·65 (0·38-1·08)
VANQWISH, 1998	9632444	28/462	10/458	2·78 (1·36-5·65)
TIMI IIIB, 1994	8149520	18/740	18/733	0·99 (0·52-1·89)
**Random effects model (I^2=59%)**		**165/4917**	**170/4932**	**0·95 (0·66-1·38)**
**Follow-up at 1 year**				
Hirlekar *et al*, 2020	32586153	10/93	14/93	0·71 (0·33-1·53)
Lee *et al*, 2019	31362541	4/31	1/29	3·74(0·44-31·55)
MOSCA, 2016	27423981	15/52	17/54	0·92 (0·51-1·64)
Italian Elderly ACS, 2012	22995877	19/154	22/159	0·89 (0·50-1·58)
OASIS 5, 2009	19202154	8/92	1/92	8·00(1·02-62·68)
ICTUS, 2005	16162880	15/604	15/596	0·99 (0·49-2·00)
Eisenberg *et al*, 2005	15894961	4/42	4/46	1·10 (0·29-4·11)
RITA 3, 2002	12241831	41/895	36/915	1·16 (0·75-1·80)
TRUCS, 2000	11071801	3/76	9/72	0·32 (0·09-1·12)
FRISC-II, 2000	10892758	27/1222	48/1235	0·57 (0·36-0·90)
VANQWISH, 1998	9632444	61/462	37/458	1·63 (1·11-2·41)
TIMI IIIB, 1995	7594098	30/740	32/733	0·93 (0·57-1·51)
**Random effects model (I^2=50%)**		**237/4463**	**236/4482**	**0·97 (0·74-1·29)**
**Follow-up at 2 years**				
Lee *et al*, 2019	31362541	5/31	3/29	1·56 (0·41-5·95)
MOSCA, 2016	27423981	19/52	22/54	0·90 (0·55-1·45)
Dimitrov *et al*, 2013^	24191395	1/22	2/30	0·68 (0·07-7·05)
Dimitrov *et al*, 2013§	24191395	2/54	1/72	2·67(0·25-28·65)
OASIS 5, 2009	19202154	8/92	2/92	4·00(0·87-18·33)
ICTUS, 2007	17350451	27/604	26/596	1·02 (0·61-1·73)
RITA 3, 2005	16154018	52/895	61/915	0·87 (0·61-1·25)
FRISC-II, 2002	12475448	45/1222	67/1235	0·68 (0·47-0·98)
**Random effects model (I^2=8%)**		**159/2972**	**184/3023**	**0·88 (0·71-1·10)**
**Follow-up at 3 years**				
MOSCA, 2016	27423981	22/52	26/54	0·88 (0·58-1·34)
After Eighty, 2016	26794722	57/229	62/228	0·92 (0·67-1·25)
ICTUS, 2007	17350451	42/604	35/596	1·18 (0·77-1·83)
FRISC-II, 2006	16980115	63/1222	83/1235	0·77 (0·56-1·05)
RITA 3, 2005	16154018	70/895	84/915	0·85 (0·63-1·15)
**Random effects model (I^2=0%)**		**254/3002**	**290/3028**	**0·89 (0·76-1·03)**
**Follow-up at 5 years**				
ICTUS, 2010	20045278	67/604	59/596	1·12 (0·80-1·56)
FRISC-II, 2006	16980115	117/1222	124/1235	0·95 (0·75-1·21)
RITA 3, 2005	16154018	102/895	132/915	0·79 (0·62-1·01)
**Random effects model (I^2=33%)**		**286/2721**	**315/2746**	**0·92 (0·77-1·11)**
**Follow-up ≥ 10 years**				
ICTUS, 2017	28408018	156/604	138/596	1·12 (0·91-1·36)
FRISC-II, 2016	27585757	473/1222	483/1235	0·99 (0·90-1·09)
RITA 3, 2015	26227188	225/895	232/915	0·99 (0·85-1·16)
**Random effects model (I^2=0%)**		**854/2721**	**853/2746**	**1·01 (0·93-1·09)**

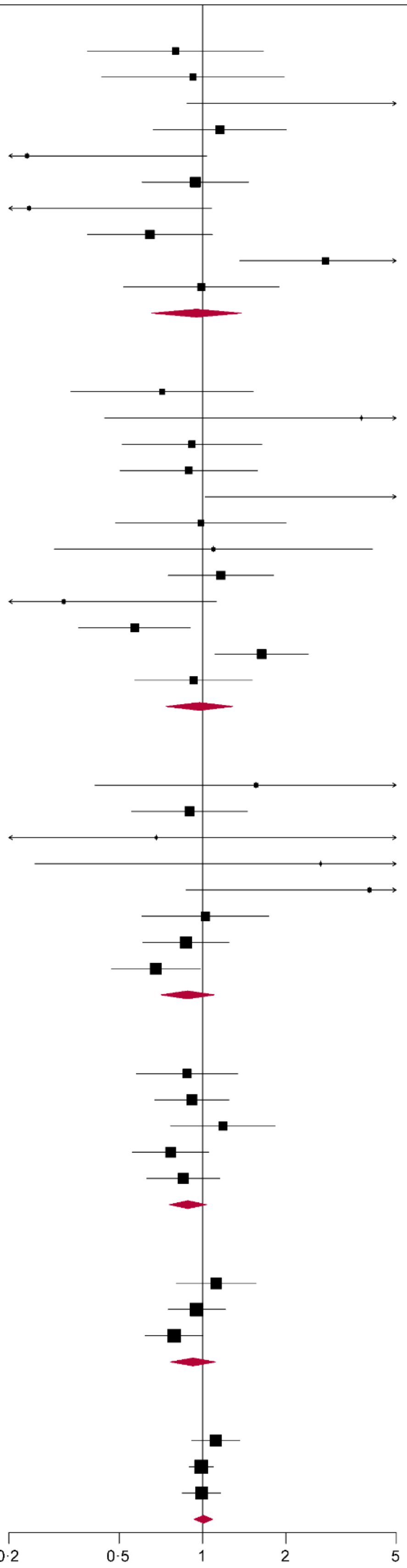

^ Diabetic group, § non-diabetic group.

Figure S4. Forest plot of in-hospital death

Study and Year	PMID	Invasive	Conservative	RR (95% CI)
Hirlekar *et al*, 2020	32586153	2/93	1/93	2·00 (0·18-21·68)
MOSCA, 2016	27423981	4/52	2/54	2·08 (0·40-10·86)
Italian Elderly ACS, 2012	22995877	8/154	5/159	1·65 (0·55- 4·94)
Eisenberg *et al*, 2005	15894961	2/42	0/46	5·47 (0·27-110·73)
TRUCS, 2000	11071801	1/76	6/72	0·16 (0·02- 1·28)
**Random effects model (I^2=27%)**		**17/417**	**14/424**	**1·35 (0·52- 3·54)**

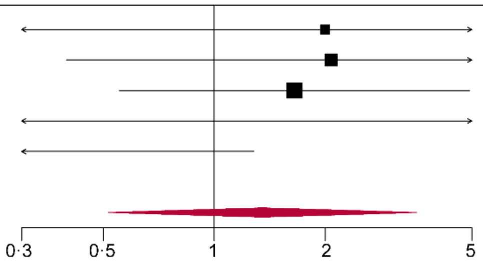

Figure S5. Forest plot of MI

Study and Year	PMID	Invasive	Conservative	RR (95% CI)
**Follow-up ≤ 6 months**				
Dimitrov *et al*, 2013	24191395	0/22	2/30	0·27 (0·01-5·38)
LIPSIA-NSTEMI, 2011	22108830	20/200	16/200	1·25 (0·67-2·34)
OASIS 5, 2009	19202154	7/92	6/92	1·17 (0·41-3·34)
RITA 3, 2002	12241831	30/895	34/915	0·90 (0·56-1·46)
VINO, 2002	11792138	2/64	10/67	0·21 (0·05-0·92)
TACTICS-TIMI 18, 2001	11419424	53/1114	76/1106	0·69 (0·49-0·97)
TRUCS, 2000	11071801	2/76	3/72	0·63 (0·11-3·67)
FRISC-II, 1999	10475181	94/1222	124/1235	0·77 (0·59-0·99)
TIMI IIIB, 1994	8149520	38/740	42/733	0·90 (0·58-1·37)
**Random effects model (I^2=0%)**		**246/4425**	**313/4450**	**0·80 (0·68-0·94)**
**Follow-up at 1 year**				
Hirlekar *et al*, 2020	32586153	11/93	19/93	0·58 (0·29-1·15)
Italian Elderly ACS, 2012	22995877	11/154	17/159	0·67 (0·32-1·38)
OASIS 5, 2009	19202154	7/92	9/92	0·78 (0·30-2·00)
ICTUS, 2005	16162880	90/604	59/596	1·51 (1·11-2·05)
Eisenberg *et al*, 2005	15894961	1/42	3/46	0·37 (0·04-3·38)
RITA 3, 2002	12241831	34/895	44/915	0·79 (0·51-1·22)
TRUCS, 2000	11071801	3/76	3/72	0·95 (0·20-4·54)
FRISC-II, 2000	10892758	105/1222	143/1235	0·74 (0·58-0·94)
TIMI IIIB, 1995	7594098	61/740	68/733	0·89 (0·64-1·24)
**Random effects model (I^2=51%)**		**323/3918**	**365/3941**	**0·86 (0·67-1·10)**
**Follow-up at 2 years**				
Lee *et al*, 2019	31362541	9/31	13/29	0·65 (0·33-1·28)
Dimitrov *et al*, 2013^	24191395	1/22	6/30	0·23 (0·03-1·76)
Dimitrov *et al*, 2013§	24191395	1/54	6/72	0·22 (0·03-1·79)
OASIS 5, 2009	19202154	11/92	12/92	0·92 (0·43-1·97)
ICTUS, 2007	17350451	98/604	67/596	1·44 (1·08-1·93)
FRISC-II, 2002	12475448	111/1222	156/1235	0·72 (0·57-0·91)
**Random effects model (I^2=73%)**		**231/2025**	**260/2054**	**0·81 (0·52-1·27)**
**Follow-up at 3 years**				
MOSCA, 2016	27423981	16/52	11/54	1·51 (0·78-2·94)
After Eighty, 2016	26794722	39/229	69/228	0·56 (0·40-0·80)
ICTUS, 2007	17350451	106/604	69/596	1·52 (1·14-2·01)
FRISC-II, 2006	16980115	122/1222	180/1235	0·68 (0·55-0·85)
**Random effects model (I^2=89%)**		**283/2107**	**329/2113**	**0·94 (0·57-1·56)**
**Follow-up at 5 years**				
ICTUS, 2010	20045278	82/604	68/596	1·19 (0·88-1·61)
FRISC-II, 2006	16980115	141/1222	195/1235	0·73 (0·60-0·89)
RITA 3, 2005	16154018	46/895	57/915	0·83 (0·57-1·20)
**Random effects model (I^2=71%)**		**269/2721**	**320/2746**	**0·89 (0·65-1·21)**

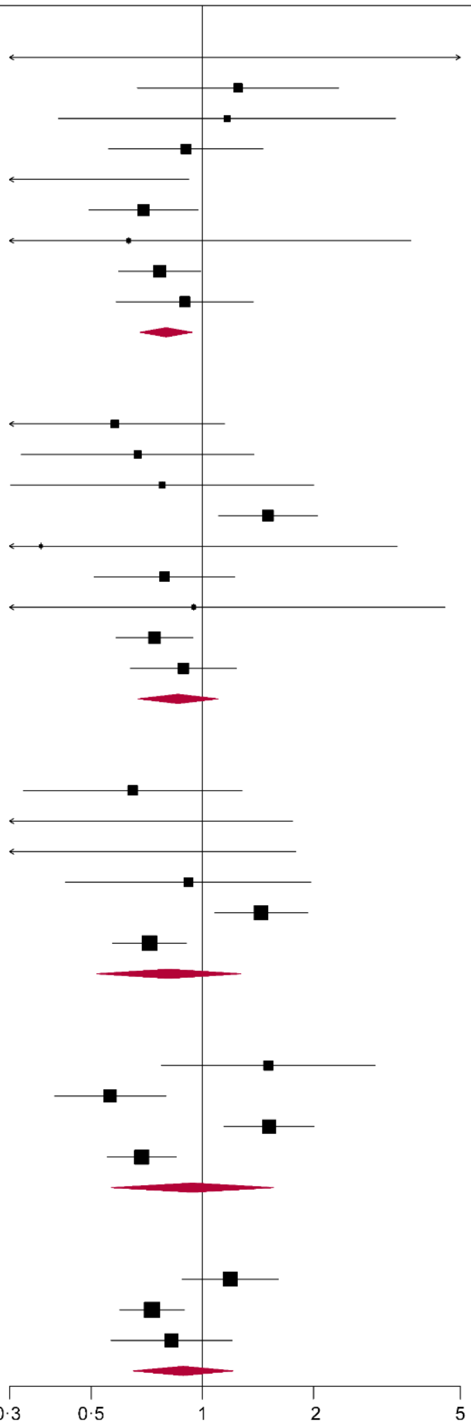

^ Diabetic group, § non-diabetic group.

Figure S6. Forest plot of CV death

Study and Year	PMID	Invasive	Conservative	RR (95% CI)
**Follow-up at 5 years**				
ICTUS, 2010	20045278	38/604	40/596	0·94 (0·61-1·44)
FRISC-II, 2006	16980115	68/1222	72/1235	0·95 (0·69-1·32)
RITA 3, 2005	16154018	64/895	92/915	0·71 (0·52-0·97)
**Random effects model (I^2=0%)**		**170/2721**	**204/2746**	**0·84 (0·69-1·02)**
**Follow-up ≥ 10 years**				
ICTUS, 2017	28408018	97/604	85/596	1·13 (0·86-1·47)
FRISC-II, 2016	27585757	175/1222	198/1235	0·89 (0·74-1·08)
RITA 3, 2015	26227188	135/895	147/915	0·94 (0·76-1·16)
**Random effects model (I^2=0%)**		**407/2721**	**430/2746**	**0·96 (0·84-1·08)**

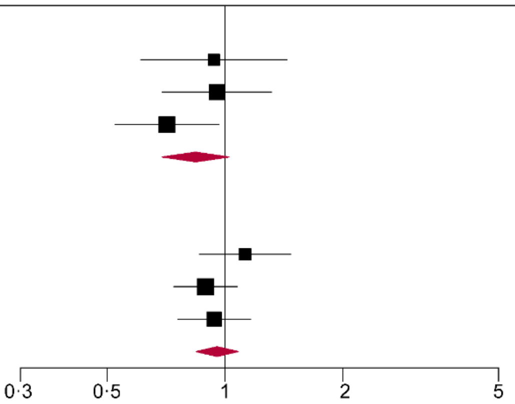

Figure S7. Forest plot of rehospitalization

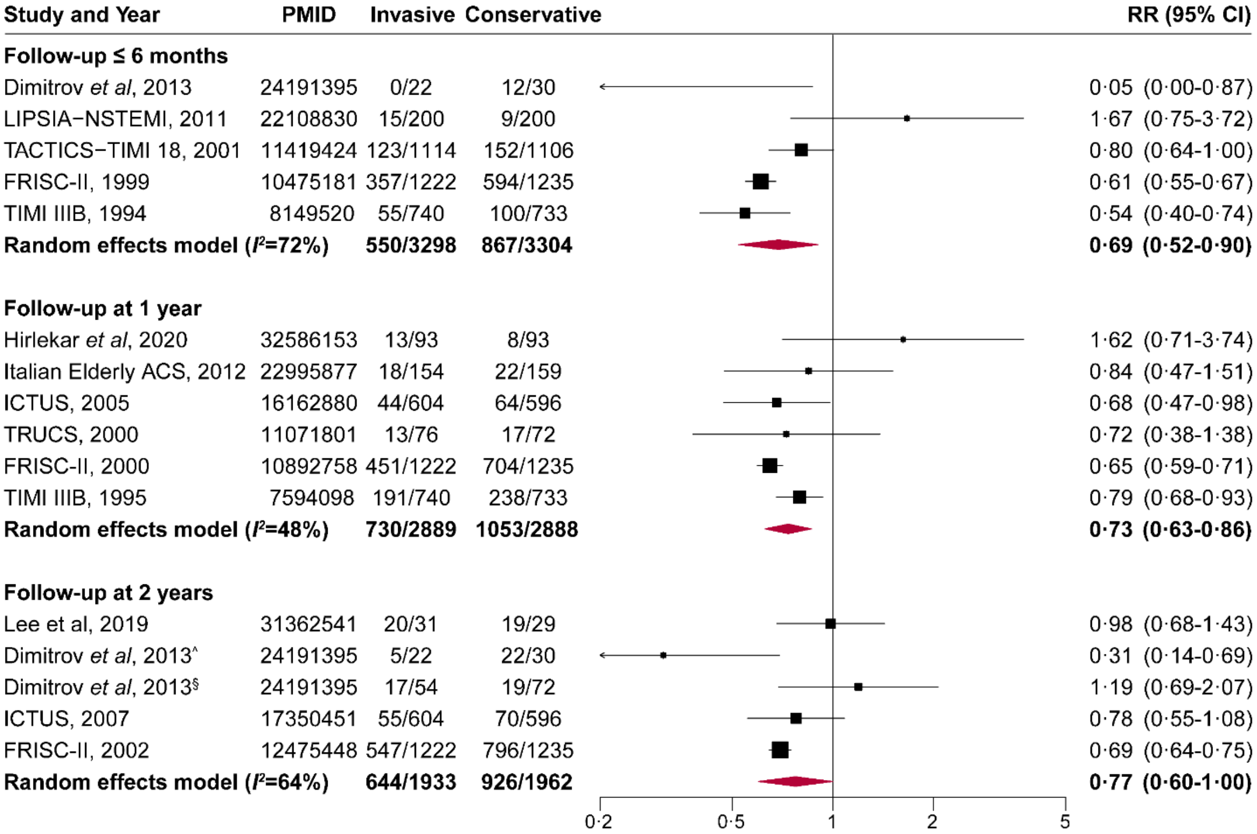

^ Diabetic group, § non-diabetic group.

Figure S8. Forest plot of bleeding

Study	PMID	Invasive	Conservative	RR (95% CI)
**Follow-up ≤ 6 months**				
Hirlekar et al, 2020	32586153	4/93	2/93	2.00 (0.38-10.65)
LIPSIA-NSTEMI, 2011	22108830	1/200	2/200	0.50 (0.05-5.47)
OASIS 5, 2009	19202154	8/92	1/92	8.00 (1.02-62.68)
TACTICS-TIMI 18, 2001	11419424	61/1114	36/1106	1.68 (1.12-2.52)
FRISC-II, 1999	10475181	19/1222	9/1235	2.13 (0.97-4.70)
**Random effects model (*I*^{2}=0%)**		**93/2721**	**50/2726**	**1.80 (1.28-2.54)**
**Follow-up 2 years**				
Lee et al, 2019	31362541	4/31	7/29	0.53 (0.17-1.64)
After Eighty, 2016	26794722	4/229	4/228	1.00 (0.25-3.93)
OASIS 5, 2009	19202154	9/92	2/92	4.50 (1.00-20.26)
**Random effects model (*I*^{2}=61%)**		**17/352**	**13/349**	**1.24 (0.36-4.22)**

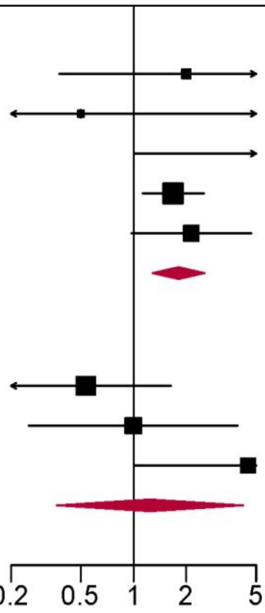

Figure S9. Forest plot of in-hospital bleeding

Study and Year	PMID	Invasive	Conservative	RR (95% CI)
Italian Elderly ACS, 2012	22995877	0/154	1/159	0·34 (0·01-8·38)
ICTUS, 2007	17350451	19/604	10/596	1·87 (0·88-4·00)
RITA 3, 2002	12241831	73/895	32/915	2·33 (1·56-3·50)
**Random effects model (I^2=0%)**		**92/1653**	**43/1670**	**2·17 (1·52-3·10)**

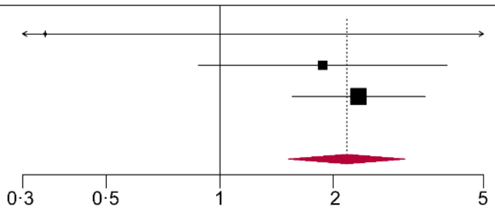

Figure S10. Forest plots of outcomes at 30 days, 6 months, 1 year and 2 years. (A) MACE, (B) Death, (C) MI, (D) Rehospitalization, (E) Bleeding.

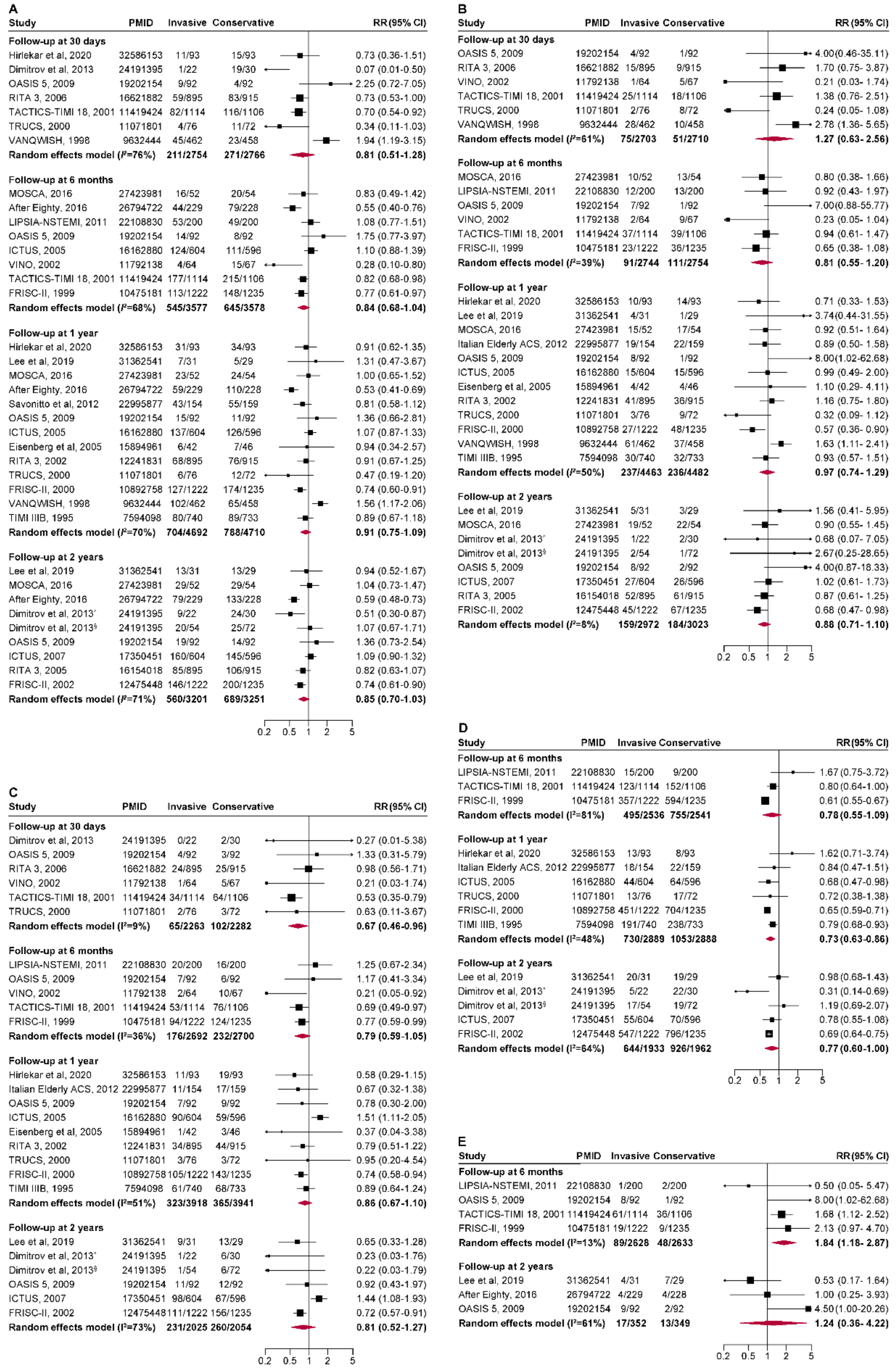

^ Diabetic group, § non-diabetic group.

Figure S11. Forest plots of MACE stratified by age. (A) Age ≥65 years, and (B) Age <65 years.

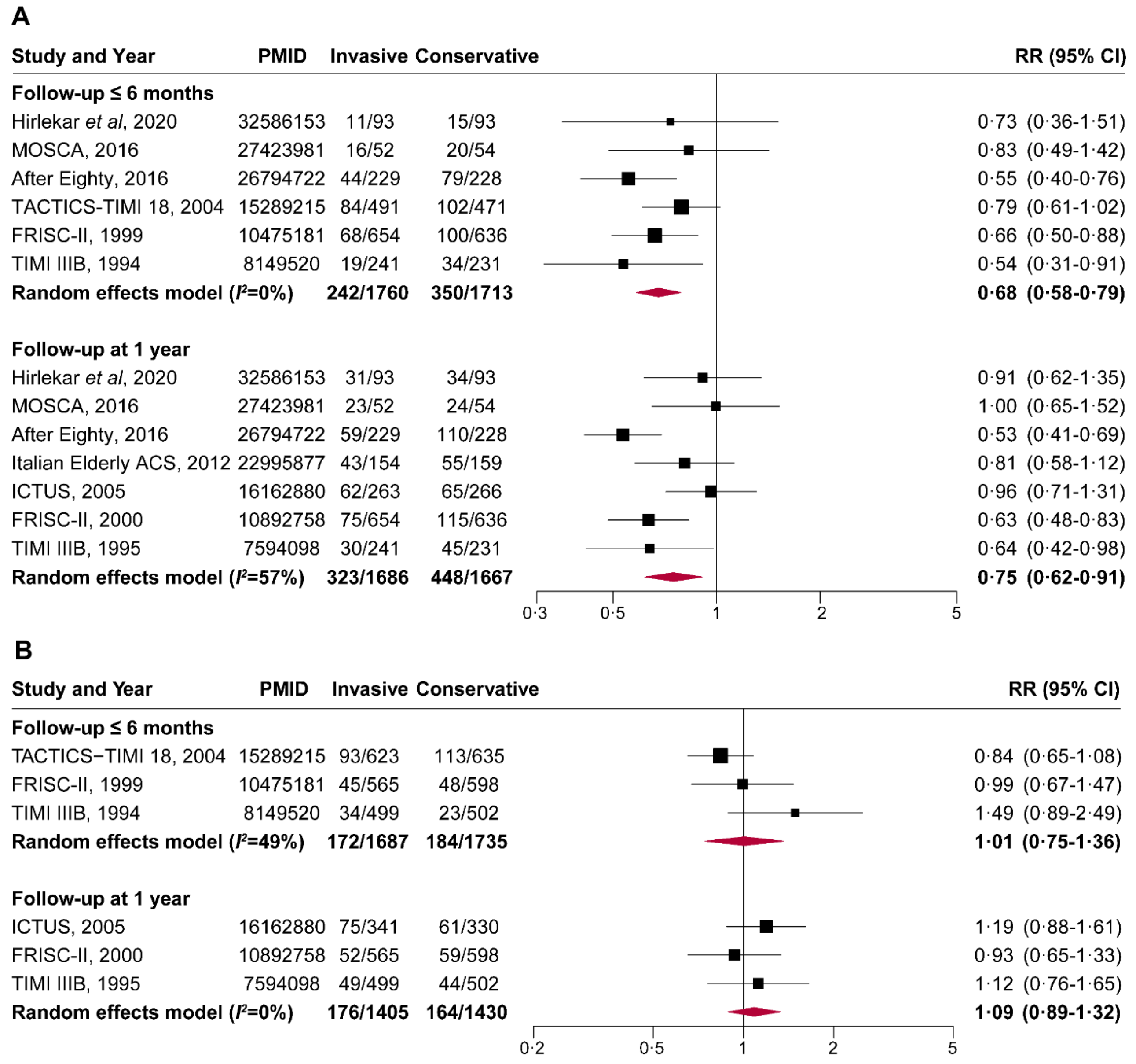

Figure S12. Forest plots of MACE stratified by gender. (A) Men, and (B) Women.

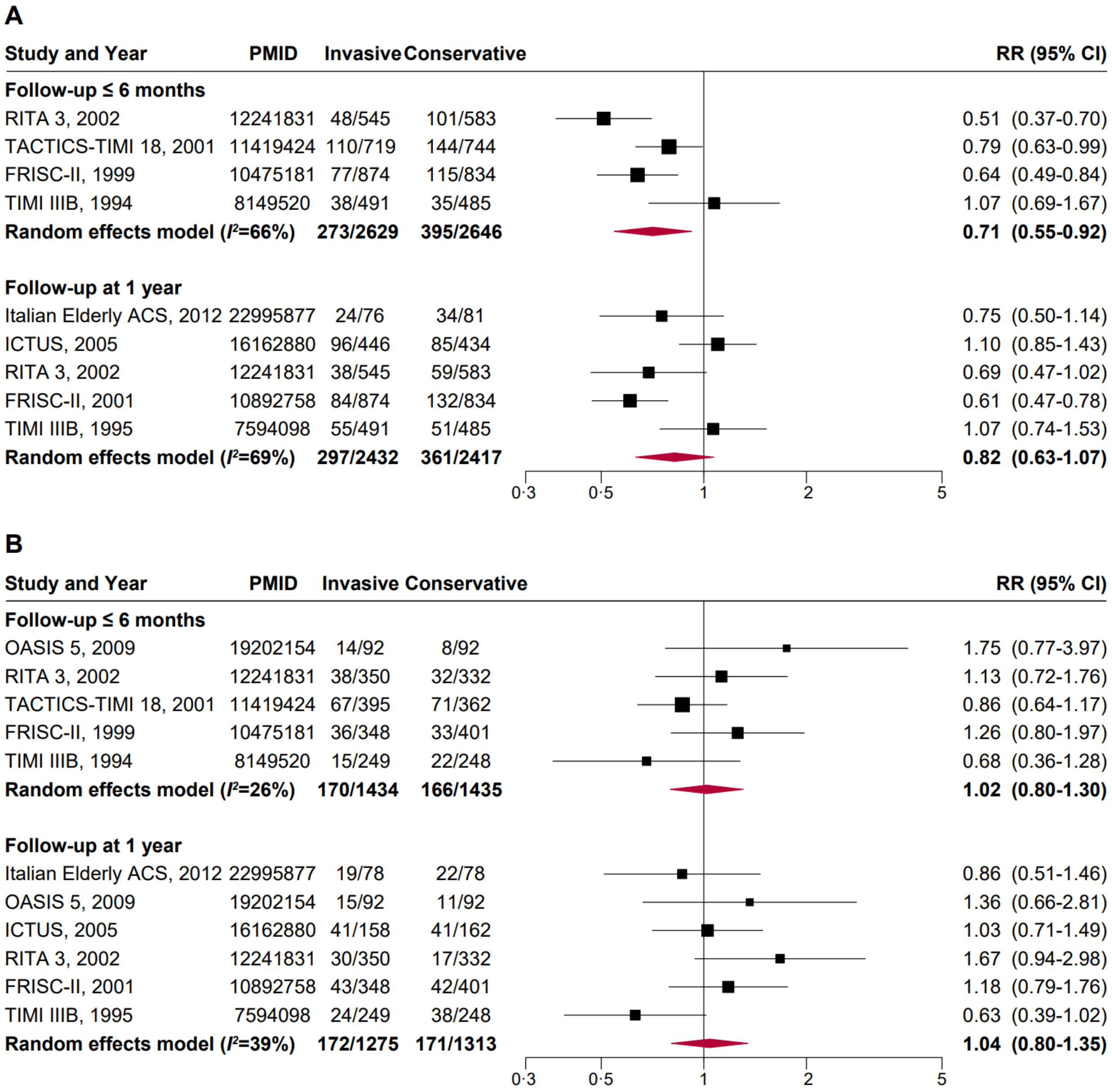

Figure S13. Forest plots of MACE stratified by diabetes. (A) Diabetes, and (B) Non-diabetes.

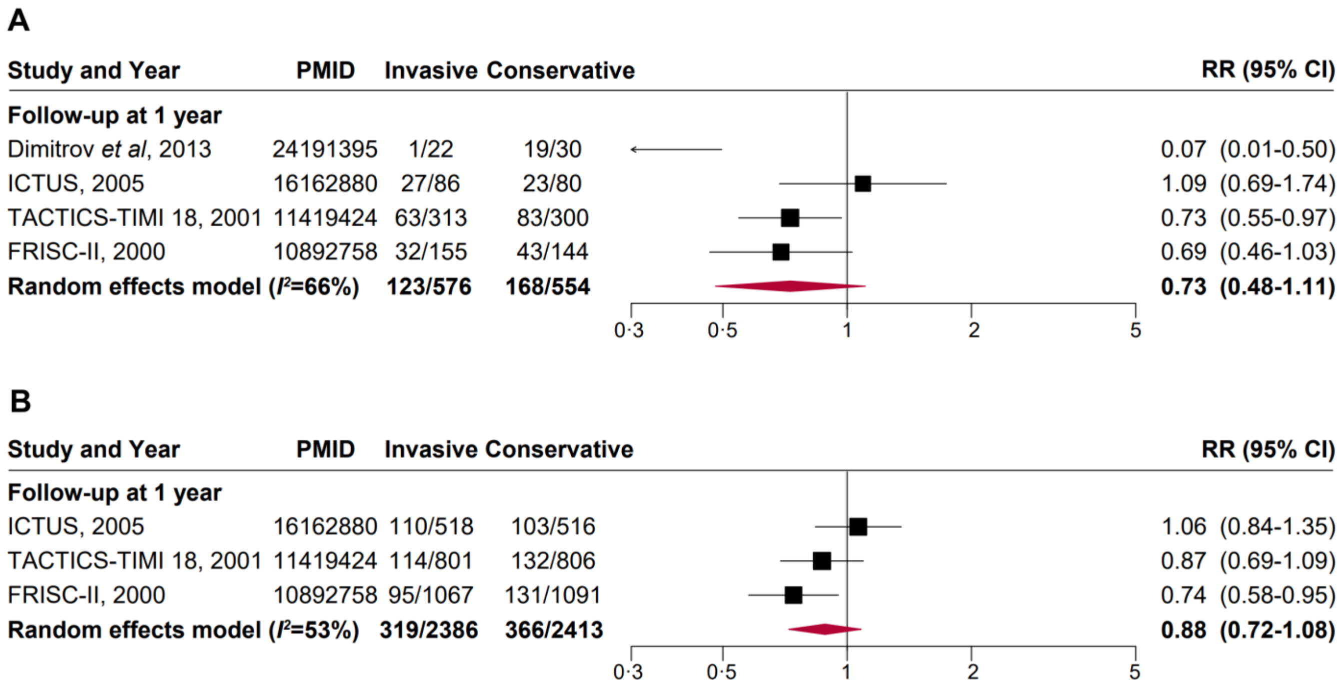

Figure S14. Forest plots of MACE stratified by ST-segment status. (A) ST-segment deviation, and (B) No ST-segment deviation.

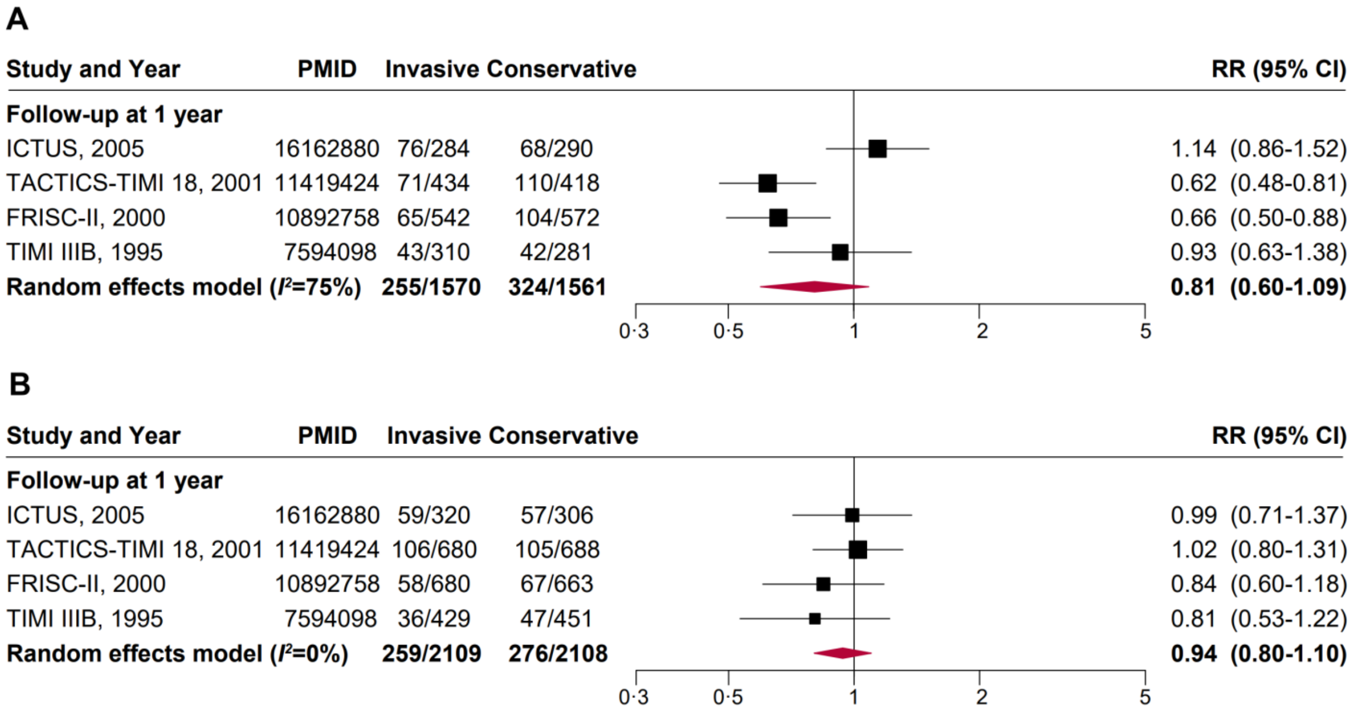

Figure S15. Forest plots of MACE stratified by troponin levels. (A) Elevated troponin levels, and (B) Normal troponin levels.

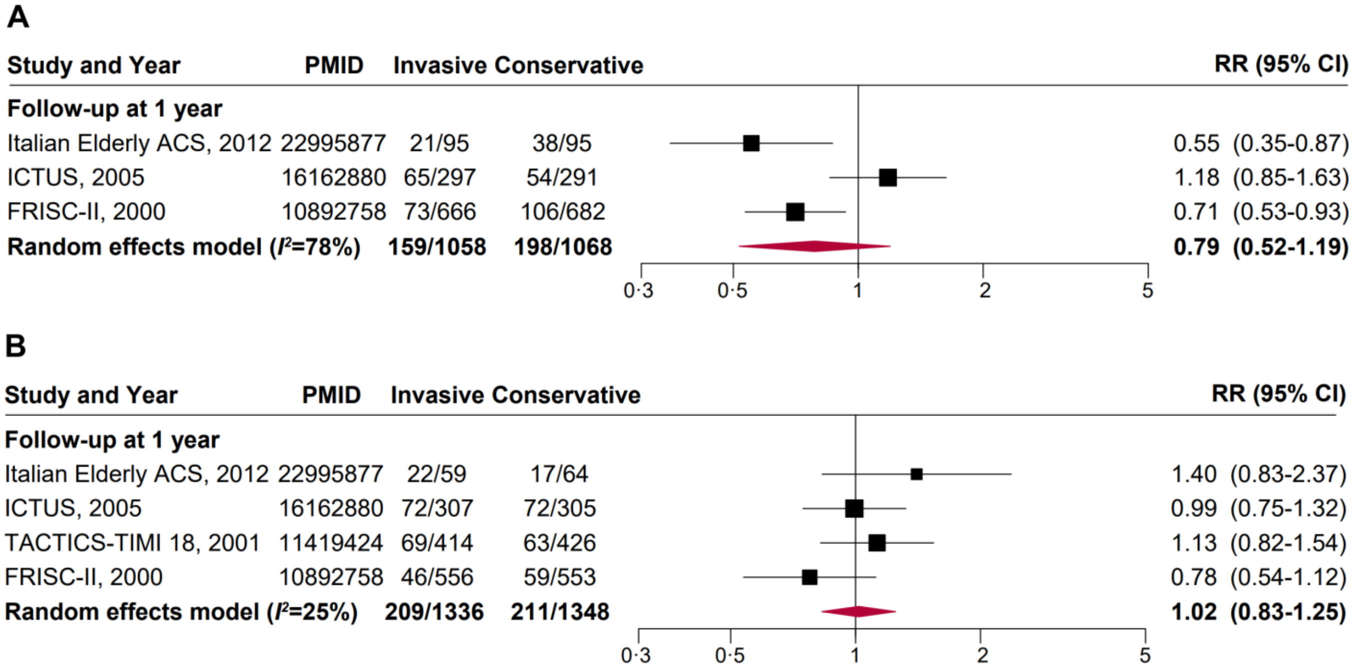

Figure S16. Sensitivity analysis of MACE. (A) Follow-up ≤ 6 months, (B) Follow-up at 1 year, (C) Follow-up at 2 years, (D) Follow-up at 3 years, and (E) Follow-up at 5 years.

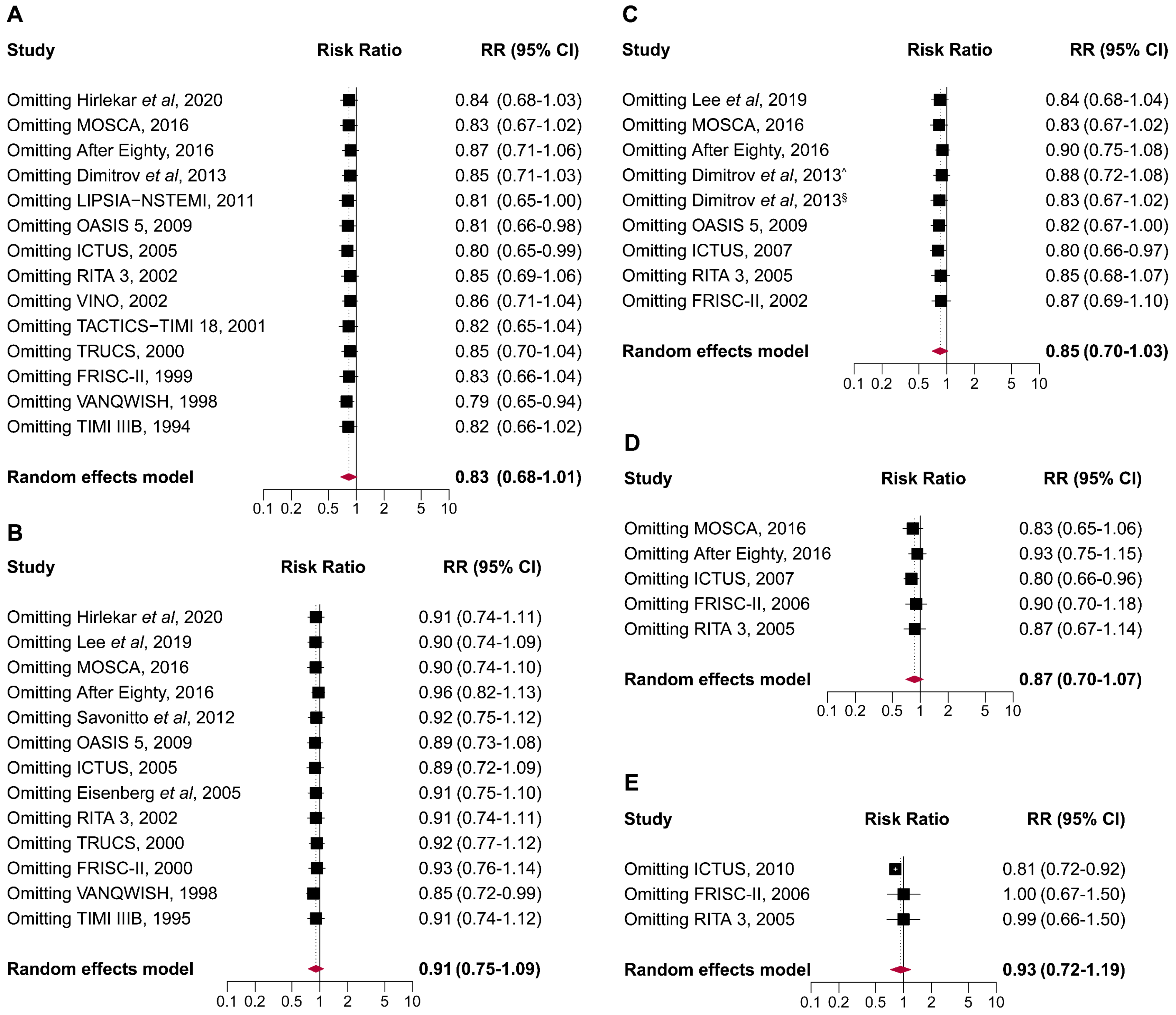

^ Diabetic group, § non-diabetic group.

Figure S17. Sensitivity analysis of death. (A) Follow-up ≤ 6 months, (B) Follow-up at 1 year, (C) Follow-up at 2 years, (D) Follow-up at 3 years, (E) Follow-up at 5 years, and (F) Follow-up ≥ 10 years.

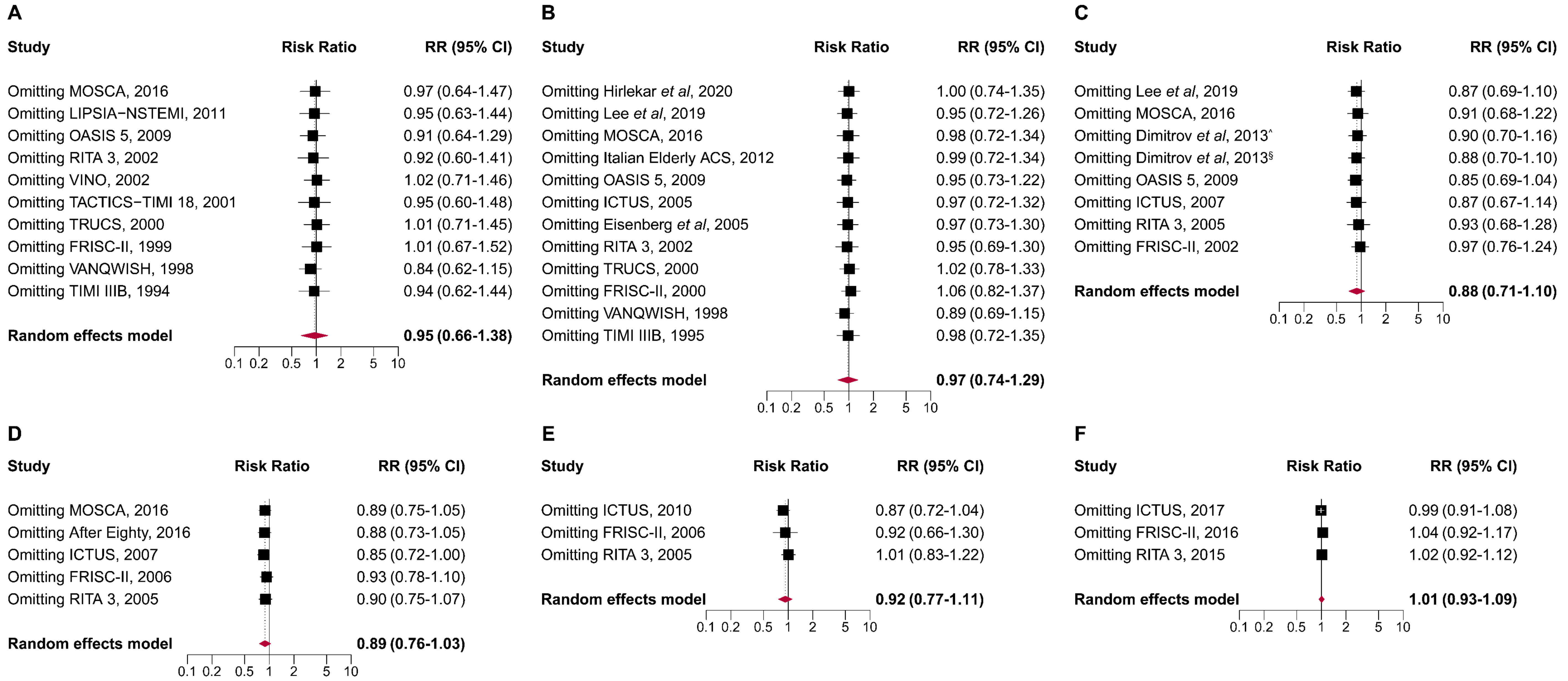

^ Diabetic group, § non-diabetic group.

Figure S18. Sensitivity analysis of MI. (A) Follow-up ≤ 6 months, (B) Follow-up at 1 year, (C) Follow-up at 2 years, (D) Follow-up at 3 years, and (E) Follow-up at 5 years.

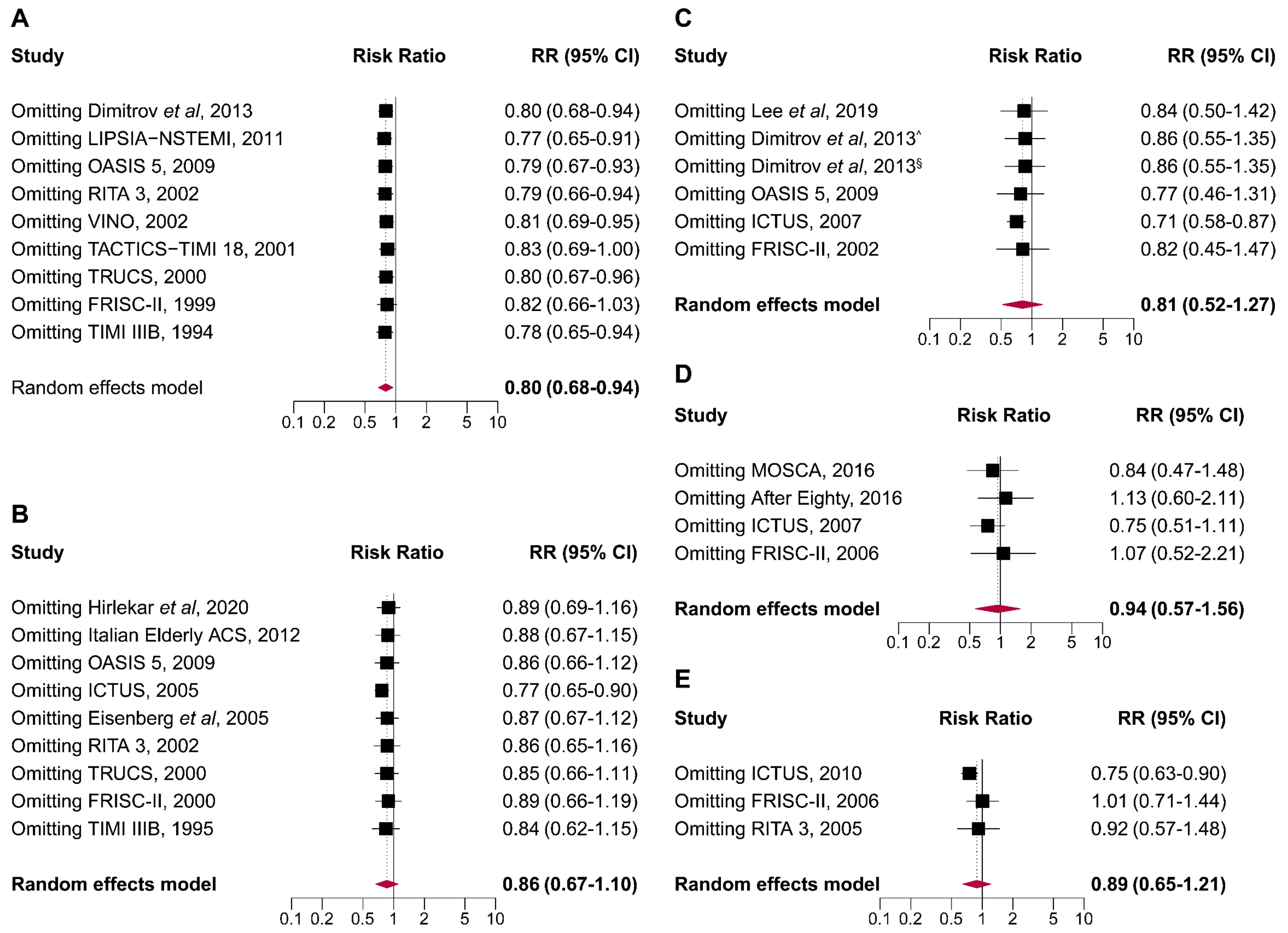

^ Diabetic group, § non-diabetic group.

Figure S19. Sensitivity analysis of CV death. (A) Follow-up at 5 years, and (B) Follow-up ≥ 10 years.

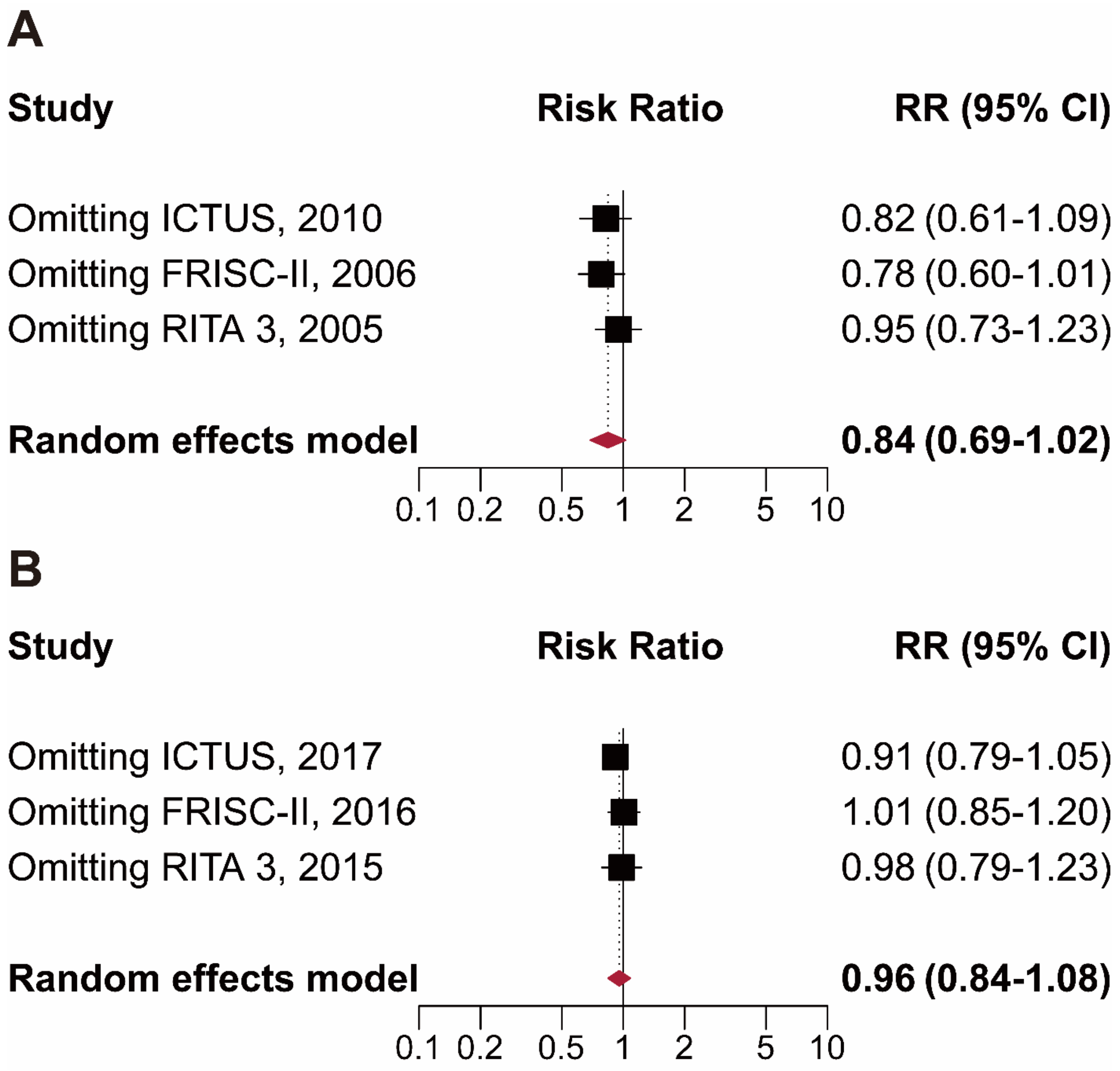

Figure S20. Sensitivity analysis of rehospitalization. (A) Follow-up ≤ 6 months, (B) Follow-up at 1 year, and (C) Follow-up at 2 years.

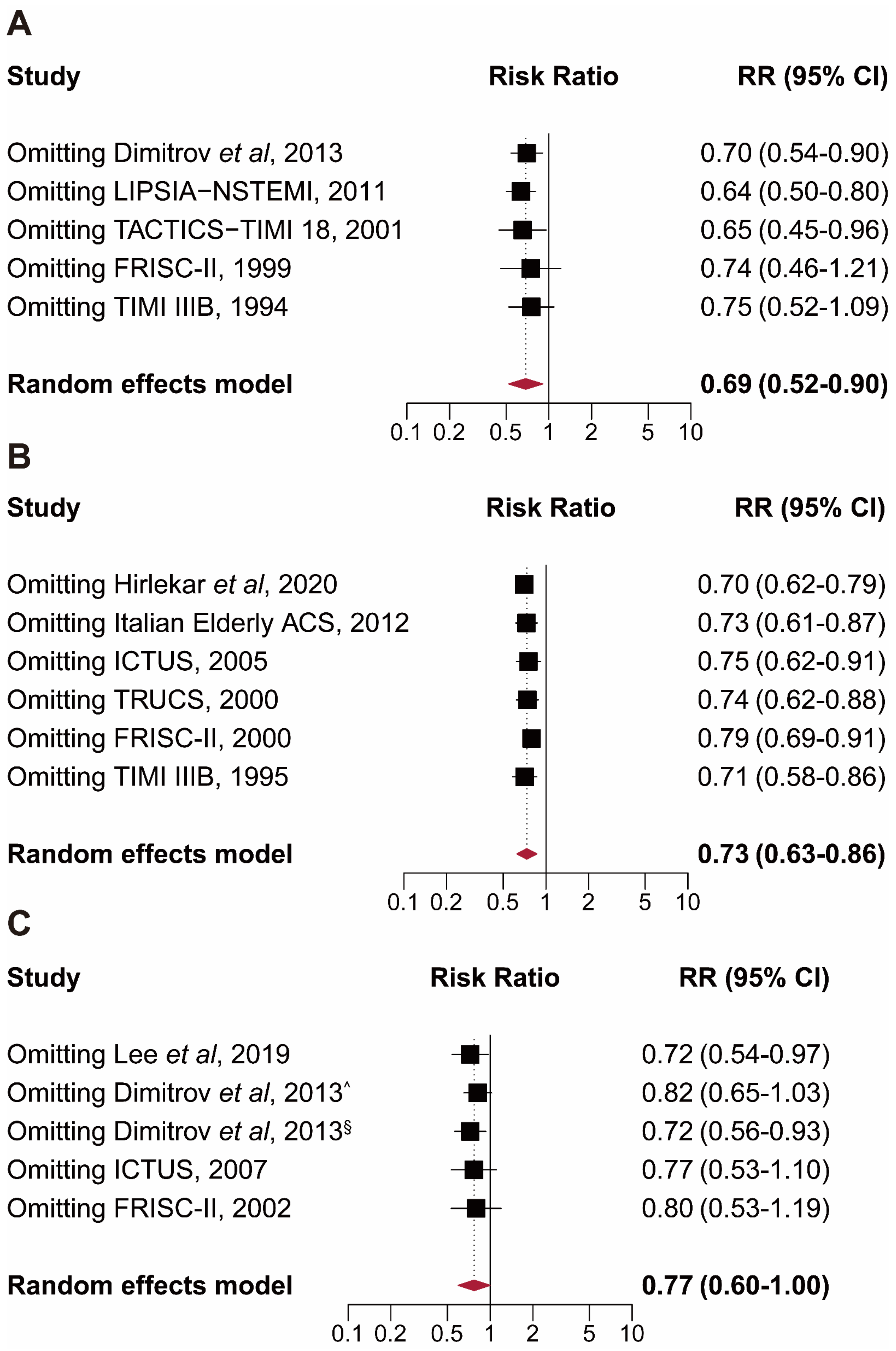

^ Diabetic group, § non-diabetic group.

Figure S21. Sensitivity analysis of bleeding. (A) Follow-up ≤ 6 months, and (B) Follow-up at 2 years.

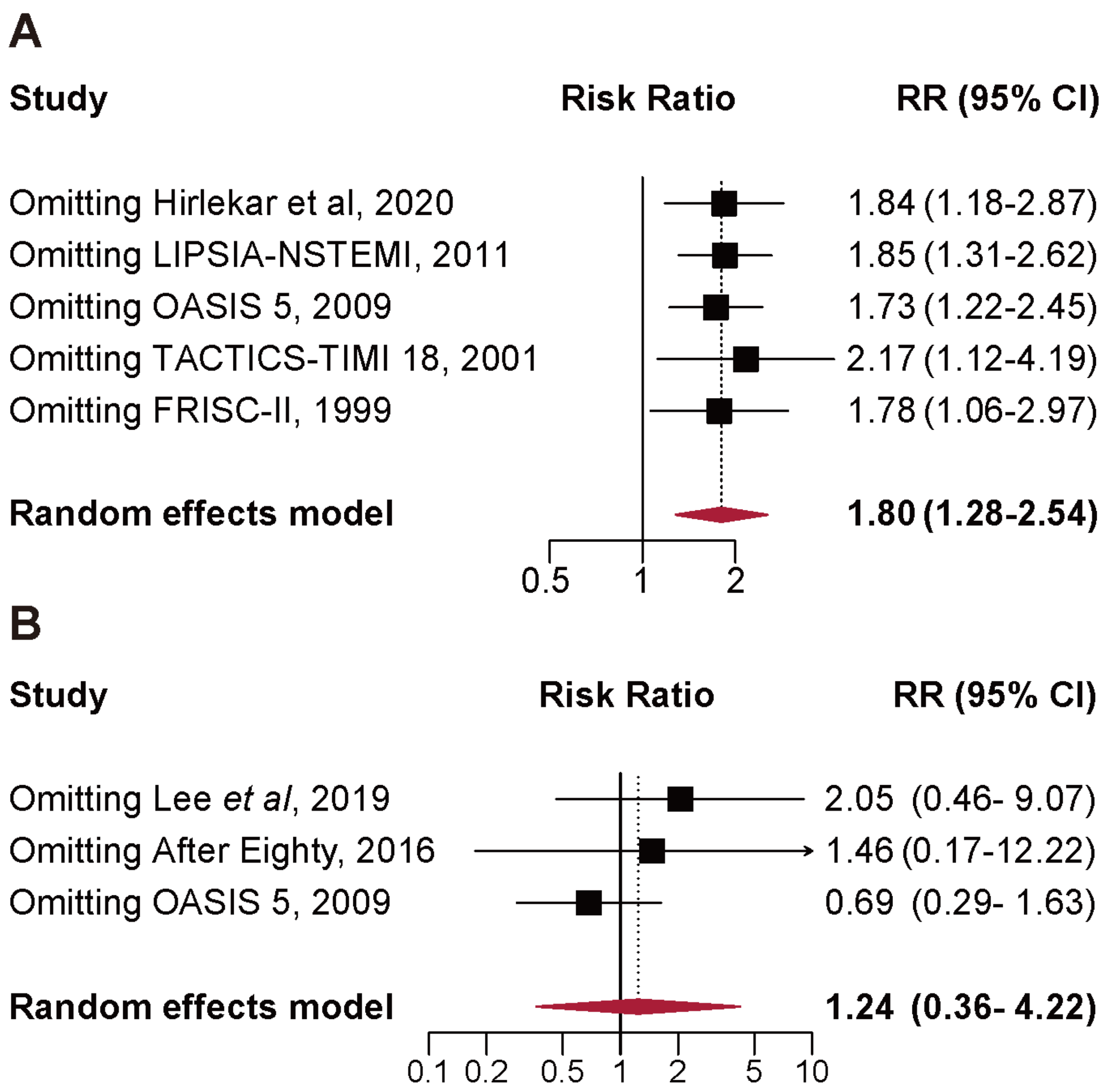

Figure S22. Sensitivity analysis of in-hospital death

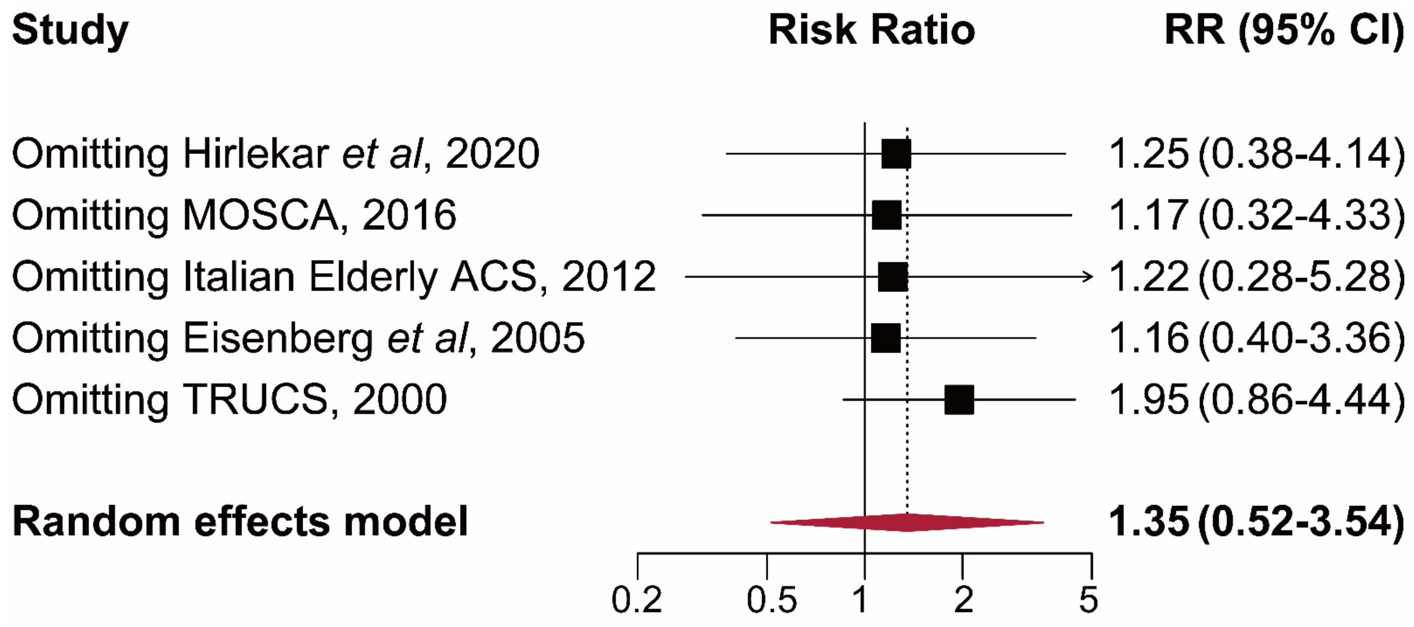

Figure S23. Sensitivity analysis of in-hospital bleeding

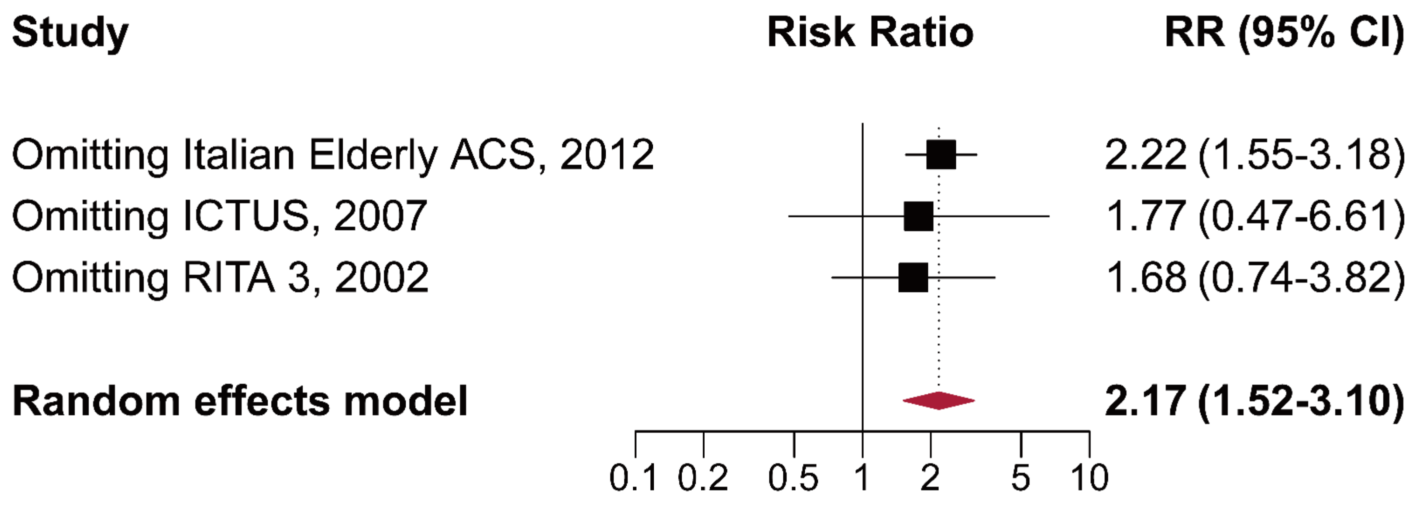

Table S4. Meta-regression results

Outcome	Follow-up	Variable	Coefficient	*p* value
MACE	≤ 6 months	Sample size	0.000	0.565
MACE	≤ 6 months	Percentage of male	-0.001	0.895
MACE	≤ 6 months	Percentage of diabetes	-0.017	0.143
MACE	≤ 6 months	Percentage of hypertension	0.004	0.662
MACE	≤ 6 months	Percentage of dyslipidemia	-0.021	0.068
MACE	≤ 6 months	Percentage of smoker	-0.011	0.465
MACE	≤ 6 months	Percentage of previous MI	-0.006	0.776
MACE	≤ 6 months	Percentage of previous PCI	-0.094	0.014
MACE	≤ 6 months	Percentage of previous CABG	-0.035	0.437
MACE	≤ 6 months	Percentage of in-hospital CAG	0.005	0.629
MACE	≤ 6 months	Percentage of in-hospital PCI	0.011	0.195
MACE	≤ 6 months	Percentage of in-hospital CABG	0.007	0.796
MACE	≤ 6 months	Percentage of ST depression	0.013	0.170
MACE	1 year	Sample size	0.000	0.879
MACE	1 year	Percentage of male	0.005	0.339
MACE	1 year	Percentage of diabetes	0.006	0.508
MACE	1 year	Percentage of hypertension	0.001	0.870
MACE	1 year	Percentage of dyslipidemia	-0.010	0.089
MACE	1 year	Percentage of smoker	-0.003	0.724
MACE	1 year	Percentage of previous MI	0.002	0.800
MACE	1 year	Percentage of previous PCI	-0.065	0.003
MACE	1 year	Percentage of previous CABG	0.003	0.670
MACE	1 year	Percentage of in-hospital CAG	0.002	0.828
MACE	1 year	Percentage of in-hospital PCI	0.003	0.602
MACE	1 year	Percentage of in-hospital CABG	0.012	0.343
MACE	1 year	Percentage of ST depression	0.017	0.055
MACE	2 years	Sample size	0.000	0.750
MACE	2 years	Percentage of male	-0.004	0.512
MACE	2 years	Percentage of diabetes	-0.004	0.338
MACE	2 years	Percentage of hypertension	0.003	0.594
MACE	2 years	Percentage of dyslipidemia	-0.007	0.364
MACE	2 years	Percentage of smoker	-0.005	0.465
MACE	2 years	Percentage of previous MI	-0.003	0.749
MACE	2 years	Percentage of previous PCI	-0.046	0.088
MACE	2 years	Percentage of previous CABG	-0.001	0.863
MACE	2 years	Percentage of in-hospital CAG	0.015	0.031
MACE	2 years	Percentage of in-hospital PCI	0.010	0.100
MACE	2 years	Percentage of in-hospital CABG	0.011	0.700
MACE	2 years	Percentage of ST depression	0.015	0.090
MACE	3 years	Sample size	0.000	0.712

MACE	3 years	Percentage of male	0.009	0.488
MACE	3 years	Percentage of diabetes	0.006	0.531
MACE	3 years	Percentage of hypertension	0.002	0.746
MACE	3 years	Percentage of dyslipidemia	-0.005	0.766
MACE	3 years	Percentage of smoker	-0.006	0.491
MACE	3 years	Percentage of previous MI	-0.004	0.771
MACE	3 years	Percentage of previous PCI	-0.043	0.409
MACE	3 years	Percentage of previous CABG	-0.068	0.252
MACE	3 years	Percentage of in-hospital CAG	0.017	0.101
MACE	3 years	Percentage of in-hospital PCI	0.012	0.153
MACE	3 years	Percentage of in-hospital CABG	0.012	0.714
MACE	3 years	Percentage of ST depression	0.010	0.359
MACE	5 years	Sample size	0.000	0.347
MACE	5 years	Percentage of male	0.032	0.467
MACE	5 years	Percentage of diabetes	0.180	0.534
MACE	5 years	Percentage of hypertension	0.046	0.368
MACE	5 years	Percentage of smoker	0.041	0.126
MACE	5 years	Percentage of previous MI	-0.027	0.785
MACE	5 years	Percentage of in-hospital CAG	0.019	0.065
Death	≤ 6 months	Sample size	0.000	0.843
Death	≤ 6 months	Percentage of male	-0.007	0.662
Death	≤ 6 months	Percentage of diabetes	-0.013	0.659
Death	≤ 6 months	Percentage of hypertension	0.004	0.816
Death	≤ 6 months	Percentage of dyslipidemia	-0.028	0.135
Death	≤ 6 months	Percentage of smoker	-0.003	0.899
Death	≤ 6 months	Percentage of previous MI	0.016	0.580
Death	≤ 6 months	Percentage of previous PCI	-0.159	0.213
Death	≤ 6 months	Percentage of previous CABG	-0.047	0.760
Death	≤ 6 months	Percentage of in-hospital CAG	-0.008	0.696
Death	≤ 6 months	Percentage of in-hospital PCI	-0.009	0.746
Death	≤ 6 months	Percentage of in-hospital CABG	-0.036	0.645
Death	≤ 6 months	Percentage of ST depression	-0.006	0.860
Death	1 year	Sample size	0.000	0.843
Death	1 year	Percentage of male	-0.003	0.796
Death	1 year	Percentage of diabetes	0.006	0.740
Death	1 year	Percentage of hypertension	0.004	0.670
Death	1 year	Percentage of dyslipidemia	-0.014	0.105
Death	1 year	Percentage of smoker	0.003	0.825
Death	1 year	Percentage of previous MI	0.018	0.233
Death	1 year	Percentage of previous PCI	-0.075	0.159
Death	1 year	Percentage of previous CABG	0.013	0.373
Death	1 year	Percentage of in-hospital CAG	-0.003	0.871

Death	1 year	Percentage of in-hospital PCI	-0.020	0.346
Death	1 year	Percentage of in-hospital CABG	-0.007	0.878
Death	1 year	Percentage of ST depression	0.022	0.708
Death	2 years	Sample size	0.000	0.118
Death	2 years	Percentage of male	-0.015	0.124
Death	2 years	Percentage of diabetes	0.001	0.872
Death	2 years	Percentage of hypertension	0.005	0.510
Death	2 years	Percentage of dyslipidemia	-0.003	0.756
Death	2 years	Percentage of smoker	0.004	0.708
Death	2 years	Percentage of previous MI	0.008	0.505
Death	2 years	Percentage of previous PCI	-0.038	0.493
Death	2 years	Percentage of previous CABG	0.002	0.885
Death	2 years	Percentage of in-hospital CAG	0.017	0.430
Death	2 years	Percentage of in-hospital PCI	0.005	0.833
Death	2 years	Percentage of in-hospital CABG	0.023	0.796
Death	2 years	Percentage of ST depression	0.036	0.629
Death	3 years	Sample size	0.000	0.406
Death	3 years	Percentage of male	0.001	0.930
Death	3 years	Percentage of diabetes	0.000	0.954
Death	3 years	Percentage of hypertension	0.001	0.774
Death	3 years	Percentage of dyslipidemia	-0.010	0.488
Death	3 years	Percentage of smoker	0.003	0.588
Death	3 years	Percentage of previous MI	0.001	0.864
Death	3 years	Percentage of previous PCI	-0.026	0.234
Death	3 years	Percentage of previous CABG	-0.030	0.382
Death	3 years	Percentage of in-hospital CAG	0.018	0.016
Death	3 years	Percentage of in-hospital PCI	0.013	0.230
Death	3 years	Percentage of in-hospital CABG	0.029	0.405
Death	3 years	Percentage of ST depression	-0.005	0.211
Death	5 years	Sample size	0.000	0.757
Death	5 years	Percentage of male	0.030	0.087
Death	5 years	Percentage of diabetes	0.017	0.945
Death	5 years	Percentage of hypertension	0.014	0.778
Death	5 years	Percentage of smoker	0.021	0.536
Death	5 years	Percentage of previous MI	-0.043	0.384
Death	5 years	Percentage of in-hospital CAG	0.011	0.484
Death	≥ 10 years	Sample size	0.000	0.376
Death	≥ 10 years	Percentage of diabetes	0.040	0.539
Death	≥ 10 years	Percentage of hypertension	0.011	0.396
Death	≥ 10 years	Percentage of smoker	0.011	0.136
Death	≥ 10 years	Percentage of previous MI	-0.002	0.924
Death	≥ 10 years	Percentage of in-hospital CAG	0.005	0.070

MI	≤ 6 months	Sample size	0.000	0.392
MI	≤ 6 months	Percentage of male	-0.006	0.505
MI	≤ 6 months	Percentage of diabetes	-0.004	0.745
MI	≤ 6 months	Percentage of hypertension	0.008	0.226
MI	≤ 6 months	Percentage of dyslipidemia	-0.027	0.014
MI	≤ 6 months	Percentage of smoker	-0.017	0.339
MI	≤ 6 months	Percentage of previous MI	-0.008	0.584
MI	≤ 6 months	Percentage of previous PCI	-0.043	0.571
MI	≤ 6 months	Percentage of previous CABG	-0.055	0.587
MI	≤ 6 months	Percentage of in-hospital CAG	0.005	0.390
MI	≤ 6 months	Percentage of in-hospital PCI	0.009	0.365
MI	≤ 6 months	Percentage of in-hospital CABG	-0.039	0.251
MI	≤ 6 months	Percentage of ST depression	0.008	0.662
MI	1 year	Sample size	0.000	0.904
MI	1 year	Percentage of male	0.007	0.369
MI	1 year	Percentage of diabetes	-0.015	0.414
MI	1 year	Percentage of hypertension	-0.006	0.451
MI	1 year	Percentage of dyslipidemia	0.000	0.981
MI	1 year	Percentage of smoker	0.026	0.216
MI	1 year	Percentage of previous MI	-0.011	0.598
MI	1 year	Percentage of previous PCI	-0.111	0.209
MI	1 year	Percentage of previous CABG	-0.043	0.414
MI	1 year	Percentage of in-hospital CAG	0.019	0.023
MI	1 year	Percentage of in-hospital PCI	0.029	0.052
MI	1 year	Percentage of in-hospital CABG	0.078	0.004
MI	1 year	Percentage of ST depression	-0.012	0.234
MI	2 years	Sample size	0.000	0.661
MI	2 years	Percentage of male	-0.002	0.850
MI	2 years	Percentage of diabetes	-0.013	0.340
MI	2 years	Percentage of hypertension	-0.005	0.747
MI	2 years	Percentage of dyslipidemia	-0.034	0.005
MI	2 years	Percentage of smoker	-0.005	0.711
MI	2 years	Percentage of previous MI	-0.012	0.390
MI	2 years	Percentage of previous PCI	-0.119	0.192
MI	2 years	Percentage of previous CABG	-0.007	0.365
MI	2 years	Percentage of in-hospital CAG	0.030	0.011
MI	2 years	Percentage of in-hospital PCI	0.030	0.122
MI	2 years	Percentage of ST depression	0.040	0.820
MI	3 years	Sample size	0.000	0.710
MI	3 years	Percentage of male	0.014	0.664
MI	3 years	Percentage of diabetes	0.017	0.516
MI	3 years	Percentage of hypertension	0.006	0.703

MI	3 years	Percentage of dyslipidemia	-0.006	0.871
MI	3 years	Percentage of smoker	-0.015	0.504
MI	3 years	Percentage of previous MI	-0.005	0.892
MI	3 years	Percentage of previous PCI	-0.073	0.491
MI	3 years	Percentage of previous CABG	-0.120	0.329
MI	3 years	Percentage of in-hospital CAG	0.031	0.401
MI	3 years	Percentage of ST depression	0.019	0.589
MI	5 years	Sample size	0.000	0.147
MI	5 years	Percentage of male	0.027	0.616
MI	5 years	Percentage of diabetes	0.242	0.315
MI	5 years	Percentage of hypertension	0.054	0.163
MI	5 years	Percentage of smoker	0.045	0.031
MI	5 years	Percentage of previous MI	-0.007	0.954
MI	5 years	Percentage of in-hospital CAG	0.021	0.078
CV death	5 years	Sample size	0.000	0.890
CV death	5 years	Percentage of male	0.030	0.234
CV death	5 years	Percentage of diabetes	-0.097	0.674
CV death	5 years	Percentage of hypertension	-0.009	0.866
CV death	5 years	Percentage of smoker	0.007	0.859
CV death	5 years	Percentage of previous MI	-0.056	0.057
CV death	5 years	Percentage of in-hospital CAG	0.005	0.799
CV death	≥ 10 years	Sample size	0.199	0.524
CV death	≥ 10 years	Percentage of male	0.011	0.650
CV death	≥ 10 years	Percentage of diabetes	0.097	0.418
CV death	≥ 10 years	Percentage of hypertension	0.024	0.255
CV death	≥ 10 years	Percentage of smoker	0.022	0.018
CV death	≥ 10 years	Percentage of previous MI	-0.003	0.951
CV death	≥ 10 years	Percentage of previous CABG	0.022	0.018
CV death	≥ 10 years	Percentage of in-hospital CAG	0.010	0.082
Rehospitalization	≤ 6 months	Sample size	0.000	0.854
Rehospitalization	≤ 6 months	Percentage of male	-0.203	0.368
Rehospitalization	≤ 6 months	Percentage of diabetes	-0.024	0.366
Rehospitalization	≤ 6 months	Percentage of hypertension	0.019	0.309
Rehospitalization	≤ 6 months	Percentage of dyslipidemia	-0.058	0.068
Rehospitalization	≤ 6 months	Percentage of smoker	-0.109	0.218
Rehospitalization	≤ 6 months	Percentage of previous MI	-0.037	0.478
Rehospitalization	≤ 6 months	Percentage of in-hospital CAG	0.017	0.379
Rehospitalization	≤ 6 months	Percentage of ST depression	0.033	0.319
Rehospitalization	1 year	Sample size	0.000	0.055
Rehospitalization	1 year	Percentage of male	-0.025	0.115
Rehospitalization	1 year	Percentage of diabetes	0.010	0.541
Rehospitalization	1 year	Percentage of hypertension	0.010	0.076

Rehospitalization	1 year	Percentage of dyslipidemia	-0.012	0.186
Rehospitalization	1 year	Percentage of smoker	0.017	0.511
Rehospitalization	1 year	Percentage of previous MI	0.015	0.256
Rehospitalization	1 year	Percentage of previous PCI	0.106	0.225
Rehospitalization	1 year	Percentage of previous CABG	0.082	0.127
Rehospitalization	1 year	Percentage of in-hospital CAG	0.000	0.999
Rehospitalization	1 year	Percentage of in-hospital PCI	-0.050	0.138
Rehospitalization	1 year	Percentage of in-hospital CABG	0.082	0.127
Rehospitalization	1 year	Percentage of ST depression	-0.012	0.882
Rehospitalization	2 years	Sample size	0.000	0.889
Rehospitalization	2 years	Percentage of male	-0.017	0.925
Rehospitalization	2 years	Percentage of diabetes	-0.009	0.167
Rehospitalization	2 years	Percentage of hypertension	0.009	0.066
Rehospitalization	2 years	Percentage of dyslipidemia	-0.008	0.642
Rehospitalization	2 years	Percentage of smoker	0.010	0.489
Rehospitalization	2 years	Percentage of previous MI	0.008	0.563
Rehospitalization	2 years	Percentage of previous PCI	-0.034	0.762
Rehospitalization	2 years	Percentage of in-hospital CAG	-0.001	0.943
Bleeding	≤ 6 months	Sample size	0.000	0.756
Bleeding	≤ 6 months	Percentage of male	-0.022	0.170
Bleeding	≤ 6 months	Percentage of diabetes	-0.028	0.424
Bleeding	≤ 6 months	Percentage of hypertension	-0.009	0.789
Bleeding	≤ 6 months	Percentage of dyslipidemia	0.008	0.844
Bleeding	≤ 6 months	Percentage of smoker	-0.033	0.673
Bleeding	≤ 6 months	Percentage of previous MI	-0.022	0.533
Bleeding	≤ 6 months	Percentage of previous PCI	-0.262	0.433
Bleeding	≤ 6 months	Percentage of previous CABG	-0.049	0.831
Bleeding	≤ 6 months	Percentage of in-hospital CAG	-0.020	0.367
Bleeding	≤ 6 months	Percentage of in-hospital PCI	-0.040	0.456
Bleeding	≤ 6 months	Percentage of in-hospital CABG	-0.040	0.705
Bleeding	≤ 6 months	Percentage of ST depression	-0.061	0.443
Bleeding	2 years	Sample size	0.001	0.934
Bleeding	2 years	Percentage of male	-0.030	0.015
Bleeding	2 years	Percentage of diabetes	-0.043	0.762
Bleeding	2 years	Percentage of hypertension	-0.048	0.844
Bleeding	2 years	Percentage of smoker	-0.036	0.127
Bleeding	2 years	Percentage of previous MI	-0.043	0.179
Bleeding	2 years	Percentage of previous CABG	-0.016	0.396
Bleeding	2 years	Percentage of ST depression	0.014	0.869
In-hospital death		Sample size	0.003	0.772
In-hospital death		Percentage of male	-0.106	0.050
In-hospital death		Percentage of diabetes	0.035	0.673

In-hospital death	Percentage of hypertension	0.054	0.218
In-hospital death	Percentage of dyslipidemia	-0.010	0.837
In-hospital death	Percentage of smoker	-0.041	0.559
In-hospital death	Percentage of previous MI	0.017	0.273
In-hospital death	Percentage of previous PCI	0.235	0.515
In-hospital death	Percentage of previous CABG	0.047	0.863
In-hospital death	Percentage of in-hospital CAG	-0.109	0.067
In-hospital death	Percentage of in-hospital PCI	-0.199	0.119
In-hospital death	Percentage of in-hospital CABG	-0.189	0.007
In-hospital bleeding	Sample size	0.001	0.395
In-hospital bleeding	Percentage of male	0.024	0.762
In-hospital bleeding	Percentage of diabetes	-0.083	0.226
In-hospital bleeding	Percentage of hypertension	-0.041	0.070
In-hospital bleeding	Percentage of previous MI	-0.055	0.805
In-hospital bleeding	Percentage of in-hospital CAG	-0.001	0.984
In-hospital bleeding	Percentage of in-hospital PCI	-0.013	0.778
In-hospital bleeding	Percentage of in-hospital CABG	0.041	0.804

MACE, major adverse cardiovascular event; CV, cardiovascular; MI, myocardial infarction; CAG, coronary angiography; PCI, percutaneous coronary intervention; CABG, coronary artery bypass graft.

Figure S24. Funnel plots of MACE. (A) Follow-up ≤ 6 months, (B) Follow-up at 1 year, (C) Follow-up at 2 years, (D) Follow-up at 3 years, and (E) Follow-up at 5 years.

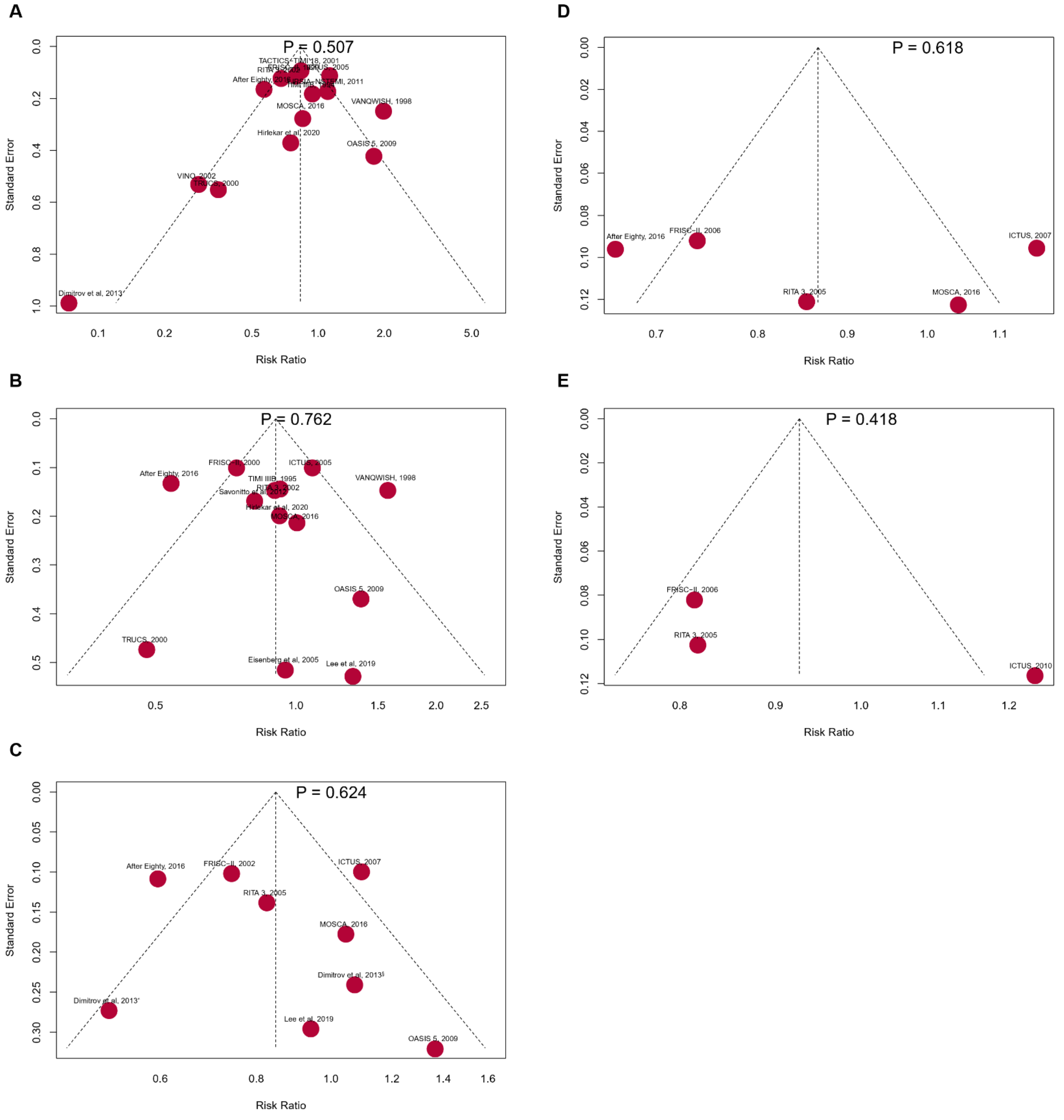

^ Diabetic group, § non-diabetic group.

Figure S25. Funnel plots of death. (A) Follow-up ≤ 6 months, (B) Follow-up at 1 year, (C) Follow-up at 2 years, (D) Follow-up at 3 years, (E) Follow-up at 5 years, and (E) Follow-up ≥ 10 years.

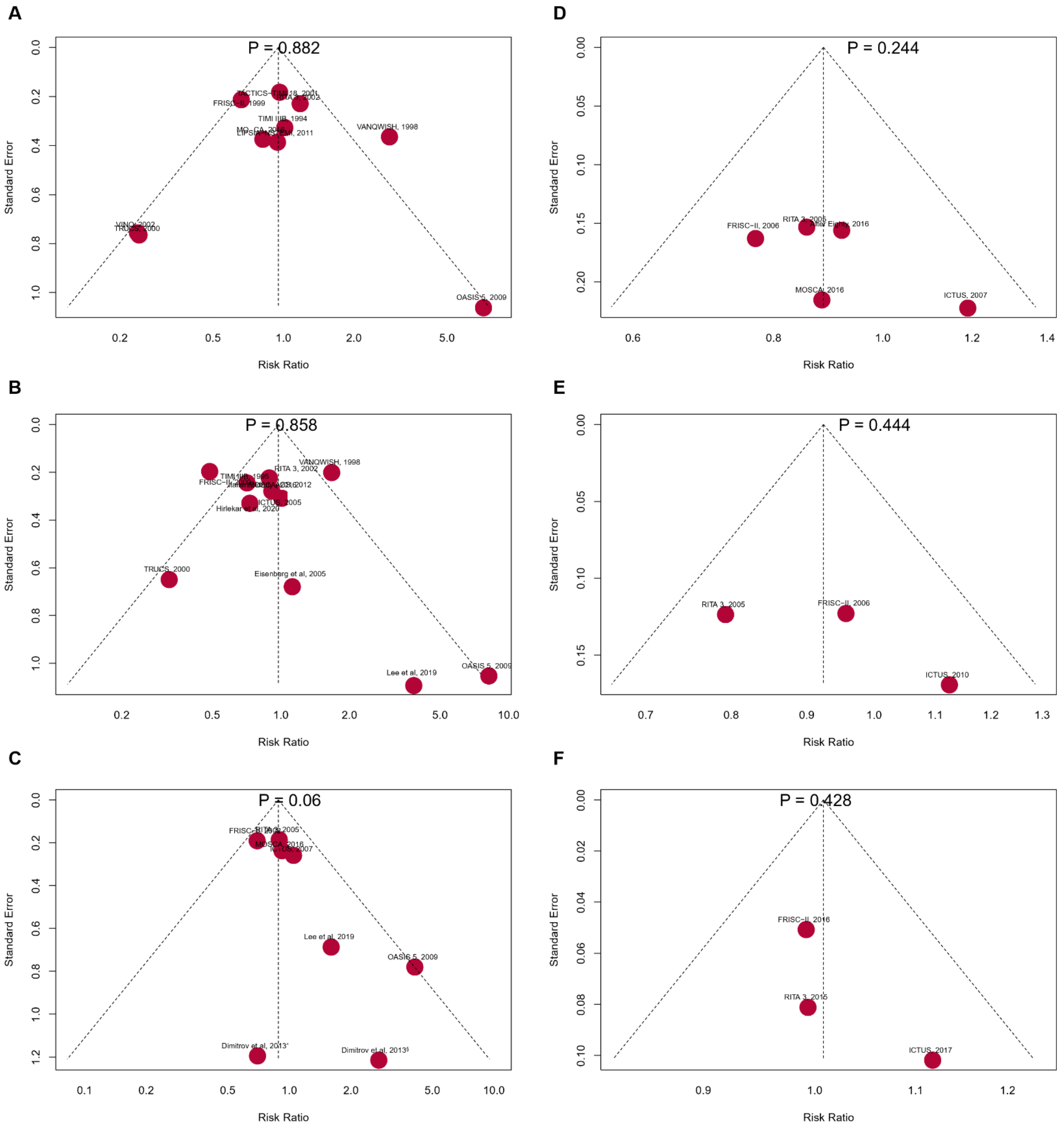

^ Diabetic group, § non-diabetic group.

Figure S26. Funnel plots of MI. (A) Follow-up ≤ 6 months, (B) Follow-up at 1 year, (C) Follow-up at 2 years, (D) Follow-up at 3 years, and (E) Follow-up at 5 years.

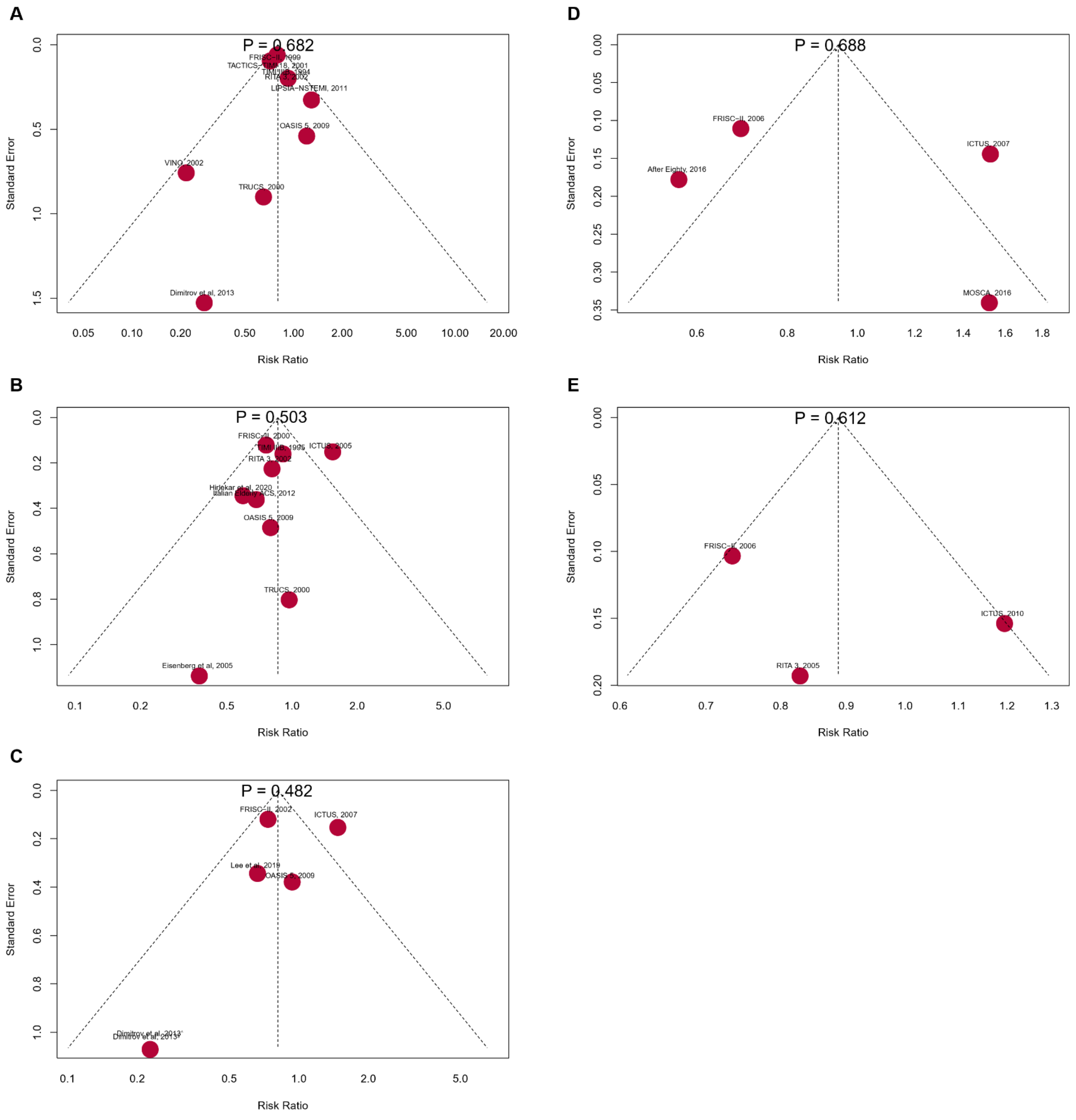

^ Diabetic group, § non-diabetic group.

Figure S27. Funnel plots of CV death. (A) Follow-up at 5 years, and (B) Follow-up ≥ 10 years.

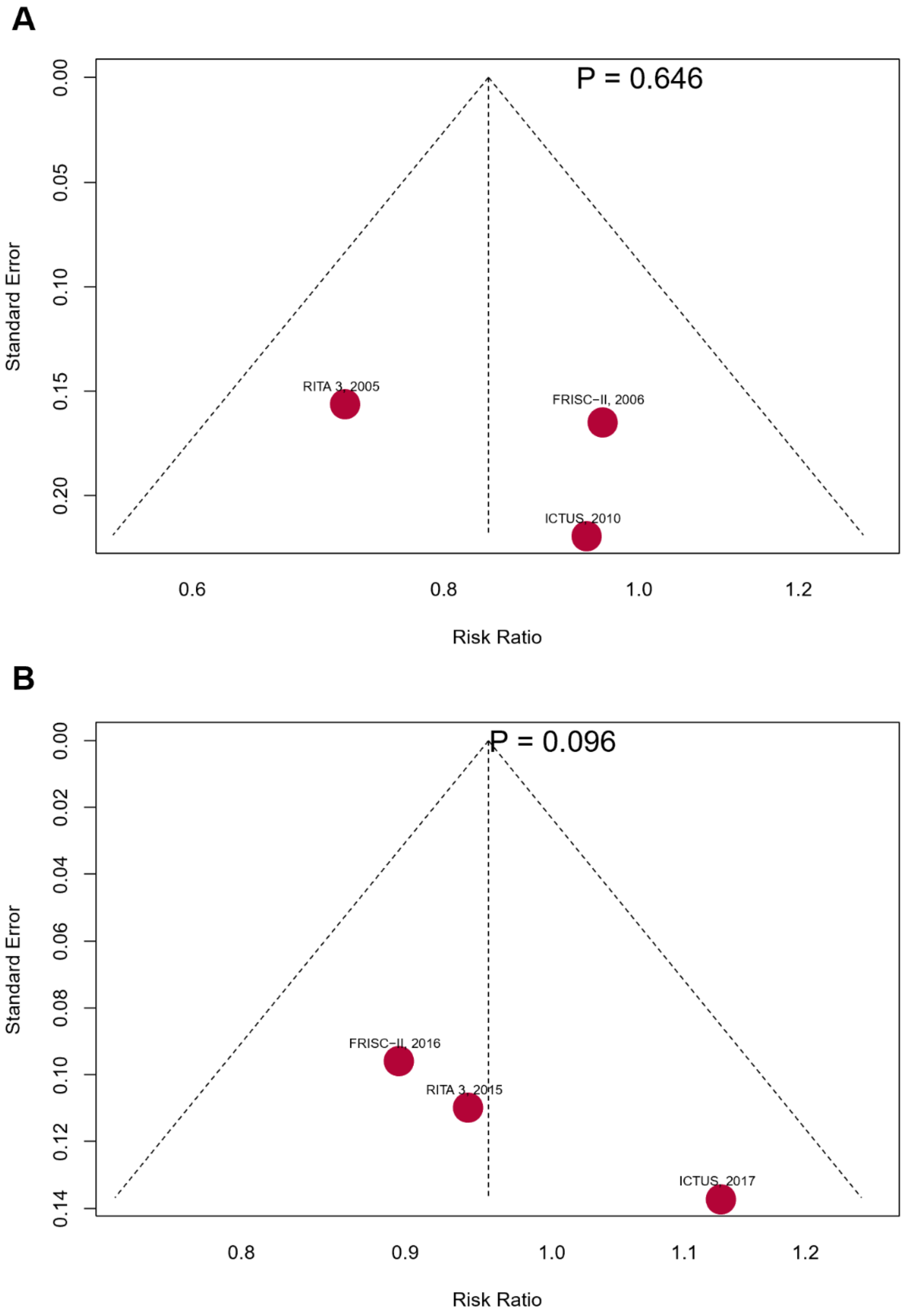

Figure S28. Funnel plots of rehospitalization. (A) Follow-up ≤ 6 months, (B) Follow-up at 1 year, and (C) Follow-up at 2 years.

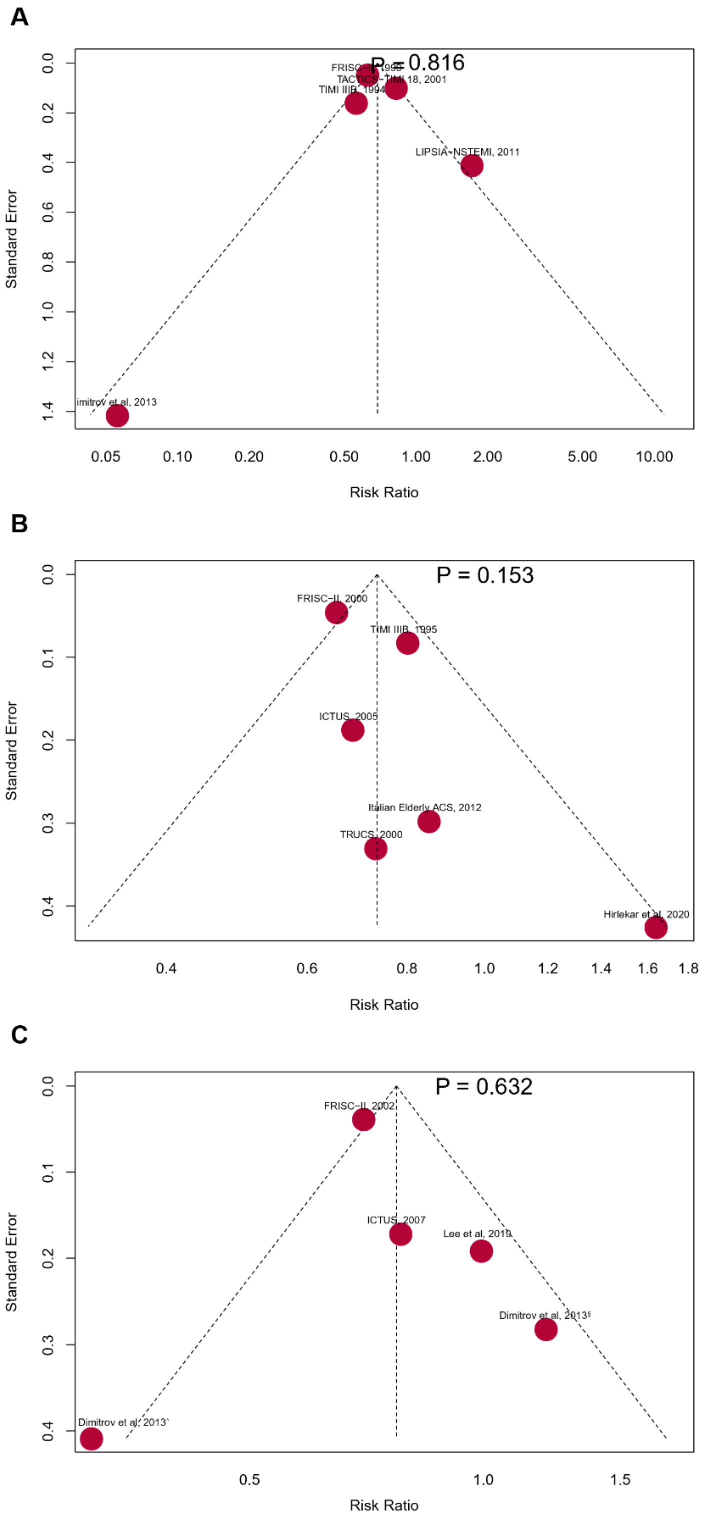

^ Diabetic group, § non-diabetic group.

Figure S29. Funnel plots of bleeding. (A) Follow-up ≤ 6 months, and (B) Follow-up at 2 years.

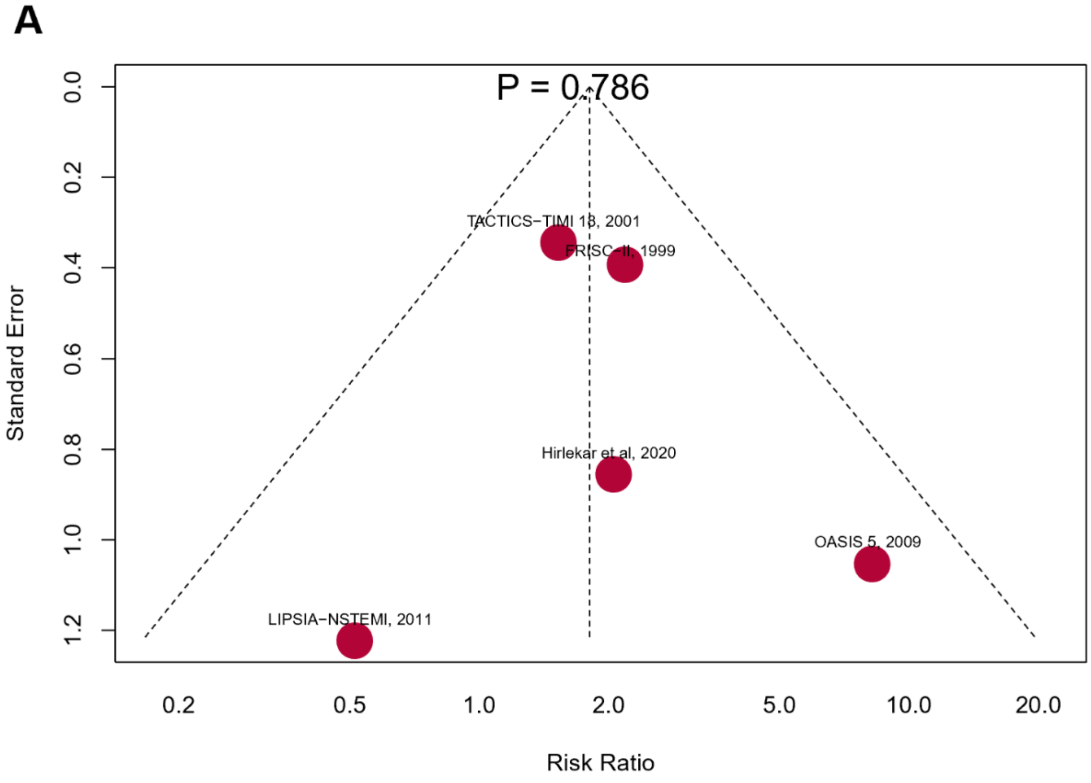

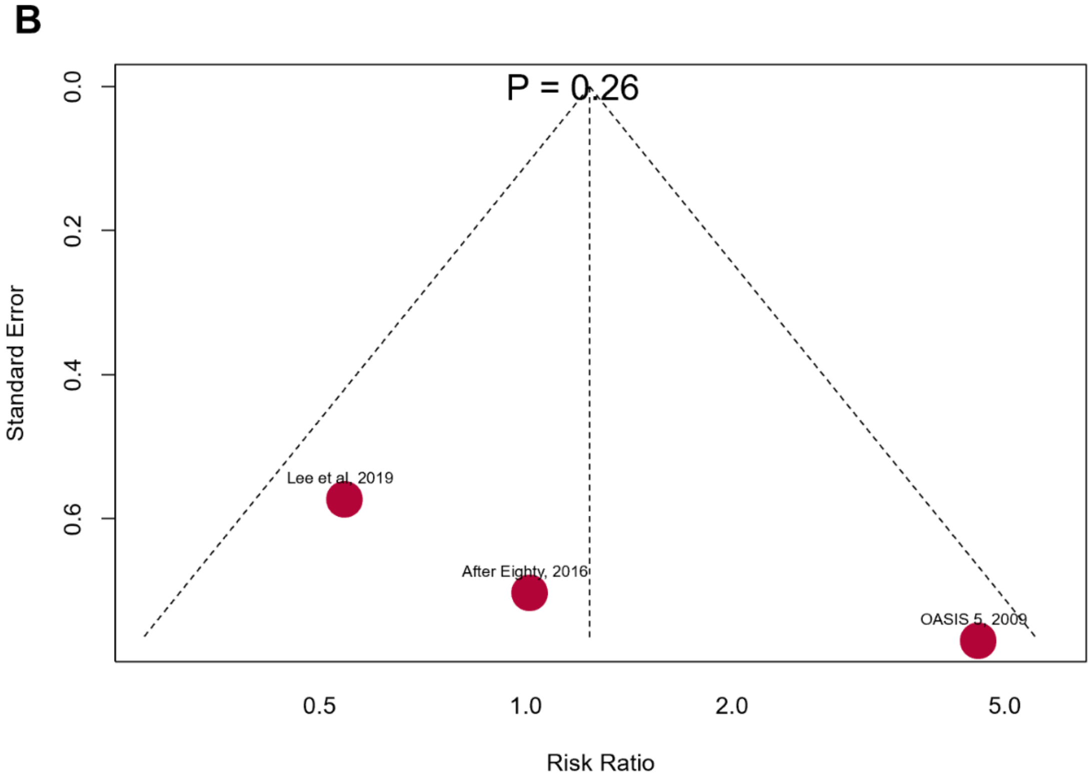

Figure S30. Funnel plots of in-hospital death.

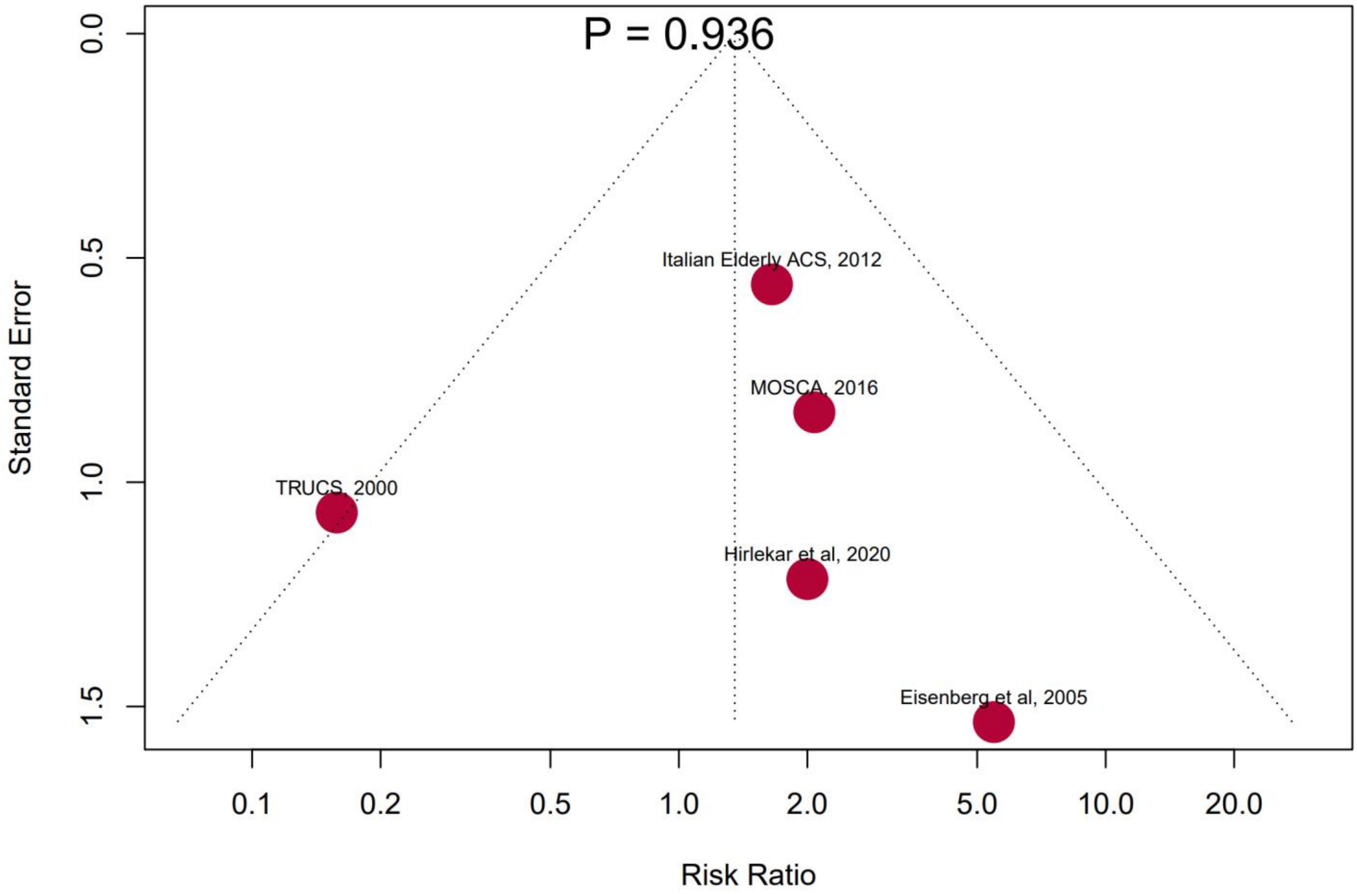

Figure S31. Funnel plots of in-hospital bleeding.

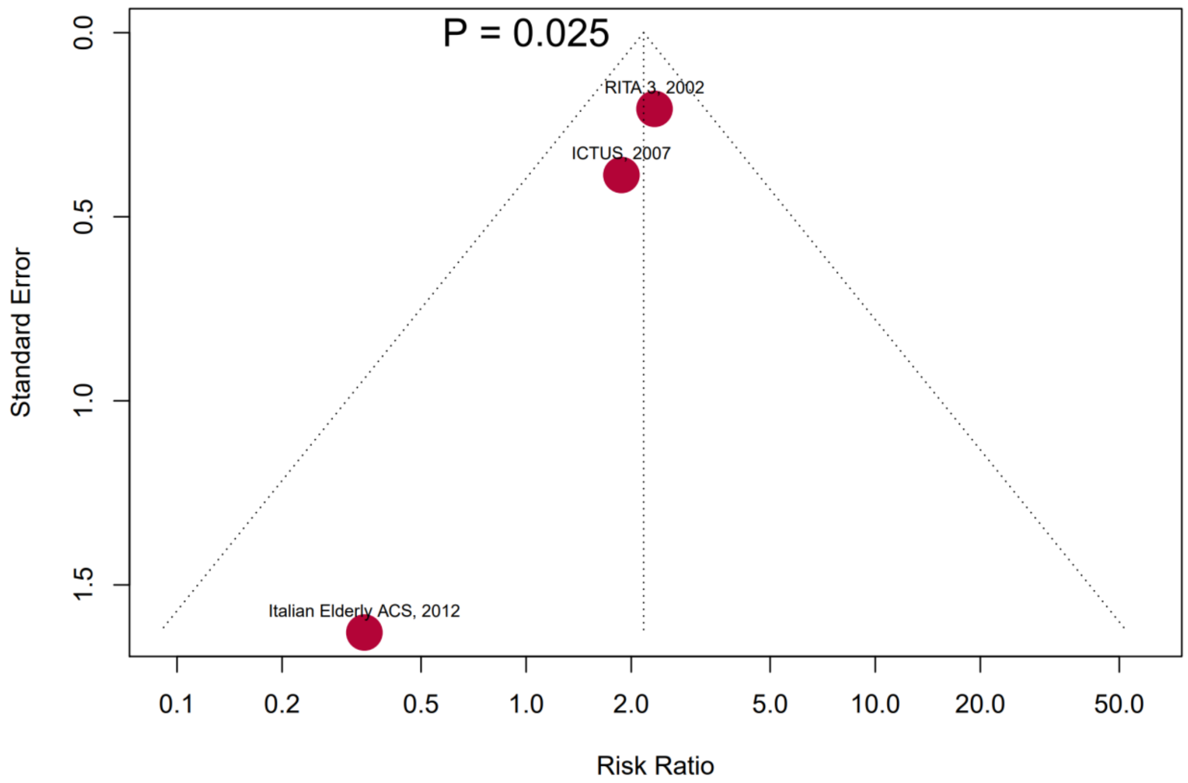

Table S5. Overview on previous meta-analyses investigating an invasive versus conservative strategy in patients with NSTE-ACS

Author, year, PMID	Included RCTs	Participants in RCTs	Follow-up	Inclusion criterion	Effect measure	Outcomes	Conclusion
Reaño JDP et al, 2020,<br>PMID: 32106261	**6 RCTs**:<br>TACTICS-TIMI 18 (6 months);<br>Italian Elderly ACS (1 year);<br>MOSCA (2.5 years);<br>After Eighty (3 years);<br>FAST-MI (3 years);<br>FRISC-II (15 years)	3768	6 months to 15 years	(1) RCT<br>(2) NSTE-ACS<br>(3) Age ≥65	RR, 95% CI	**Death**: 0.69 (0.39-1.23)<br>**CV Death**: 0.86 (0.67-1.10)<br>**MI**: 0.63 (0.39-1.04)<br>**Stroke**: 0.52 (0.26-1.03)<br>**Revascularization**: 0.29 (0.14-0.59)<br>**Recurrent angina**: 0.81 (0.45-1.46)	There was a significantly lower rate of revascularization in the invasive strategy group compared to the conservative treatment group. In the reduction of all-cause mortality, cardiovascular mortality, MI, and stroke there was no significant effect of invasive strategy versus conservative treatment. This finding does not support the bias against early routine invasive intervention in patients ≥ 65 years old with NSTE-ACS.
Garg A et al, 2018,<br>PMID: 29439831	**3 RCTs**:<br>TACTICS TIMI 18 (6 months);<br>Italian ACS elderly (1 year);<br>After Eighty (3 years)<br>**1 Pooled RCT**:<br>FIR (FRISC-II, ICTUS, RITA 3) (5 years)	1887	6 months to 5 years	(1) RCT<br>(2) NSTE-ACS<br>(3) Age ≥75	OR, 95% CI	**Death**: 0.87 (0.63-1.20)<br>**CV Death**: 0.84 (0.61-1.15)<br>**MI**: 0.51 (0.40-0.66)<br>**Major Bleeding**: 1.96 (0.97-3.97)<br>**Death or MI**: 0.65 (0.51-0.83)<br>**Revascularization**: 0.31 (0.11-0.91)	In elderly patients older than 75 years with NSTE-ACS, RIS is superior to SIS for the composite end point (death or MI), primarily driven by reduced risk of MI.
Ma W et al, 2018,<br>PMID: 28802810	**3 RCTs**:<br>TACTICS-TIMI 18 (6 months);<br>Italian Elderly ACS (1 year);<br>After Eighty (1.53 years)<br>**1 pooled RCT**:<br>FIR (FRISC-II, ICTUS, RITA 3) (5 years)	1887	6 months to 5 years	(1) RCT and OS<br>(2) NSTE-ACS<br>(3) Age ≥75	RR, 95% CI	**Death**: 0.82 (0.64-1.05)<br>**In-hospital bleeding**: 2.51 (1.53-4.11)	Elderly patients with NSTE-ACS might benefit from an early invasive strategy but with increasing risk of any bleeding complications.
Elgendy IY et al, 2017,<br>PMID: 27939385	**8 RCTs**:<br>FRISC-II (6 months);<br>Italian Elderly ACS (1 year);<br>ICTUS (1 year);<br>Eisenberg et al (1 year);<br>TRUCS (1 year);<br>OASIS 5 substudy (2 years);<br>After Eighty (3 years);<br>RITA 3 (10 years)	6657	6 months to 10 years	(1) RCT<br>(2) NSTE-ACS<br>(3) Trials undertaken in era of stents	OR, 95% CI	**Death**:<br>overall, 1.00 (0.90-1.12)<br>1-5 years, 0.90 (0.77-1.04)<br>>5 years, 1.02 (0.90-1.14)	In patients with NSTE-ACS, both routine invasive and selective invasive strategies have a similar risk of all-cause mortality at 10 years.
Gnanenthiran SR et al, 2017,<br>PMID: 28626044	**3 RCTs**:<br>TACTICS-TIMI 18 (6 months);<br>Italian Elderly ACS (1 year);<br>After Eighty (3 years)<br>**1 pooled RCT**:<br>FIR (FRISC-II, ICTUS, RITA 3) (5 years)	1887	6 months to 5 years	(1) RCT and OS<br>(2) NSTE-ACS<br>(3) Age ≥75	OR, 95% CI	**Death**: 0.84 (0.66-1.06)<br>**MI**: 0.51 (0.40-0.66)<br>**Revascularization**: 0.27 (0.13-0.56)<br>**Major bleeding**: 2.19 (1.12-4.28)	Routine invasive therapy reduces MI and repeat revascularization and may reduce mortality at the expense of major bleeding in elderly patients with NSTE-ACS.
Fanning JP et al, 2016,<br>PMID: 8568369	**8 RCTs**:<br>FRISC-II (6 months to 5 years);<br>VINO (1 and 6 months);<br>TACTICS-TIMI 18 (1 and 6 months);<br>LIPSIA-NSTEMI (6 months);<br>Italian Elderly ACS (1 year);<br>ICTUS (1 month to 4 years);<br>OASIS 5 (1 month to 2 years);<br>RITA 3 (4 months to 5 years)	8915	Early: ≤4 months<br>Intermediate: 6 to 12 months<br>Late: ≥2 years	(1) RCT<br>(2) NSTE-ACS<br>(3) Trials undertaken in era of stents	RR, 95% CI	**Death:**<br>≤4 months: 1.18 (0.70-2.00)<br>6-12 months: 0.87 (0.64-1.18)<br>≥2 years: 0.90 (0.76-1.08)<br>**MI**:<br>≤4 months: 0.68 (0.43-1.08)<br>6-12 months: 0.79 (0.63, 1.00)<br>≥2 years: 0.78 (0.67-0.92)<br>**Rehospitalization**:<br>6-12 months, 0.77 (0.63-0.94)<br>**Bleeding**: 1.73 (1.3-2.31)	The evidence failed to show appreciable benefit with routine invasive strategies for unstable angina and non-ST elevation MI compared to conservative strategies in all-cause mortality and death or non-fatal MI at 6 to 12 months. There was evidence of risk reduction in MI, refractory angina and re-hospitalization with routine invasive strategies compared to conservative (selective invasive) strategies at six to 12 months follow-up. However, routine invasive strategies were associated with a relatively high risk (almost double the risk) of procedure-related MI, and increased risk of bleeding complications.

						**In-hospital death**: 1.54 (1.02-2.34)<br>**Death or MI**:<br>≤4 months: 0.64 (0.45-0.92)<br>6-12 months: 0.93 (0.71-1.20)<br>≥2 years: 0.89 (0.73-1.08)	
Alfredsson J et al, 2014,<br>PMID: 25262262	**3 RCTs**:<br>FRISC-II (5 years);<br>ICTUS (5 years);<br>RITA 3 (5 years)	5467	5 years	NA	HR, 95% CI	**CV Death**:<br>men: 0.71 (0.56-0.89)<br>women: 0.97 (0.68-1.39)<br>**MI**:<br>men: 0.69 (0.57-0.83)<br>women: 1.13 (0.85-1.50)<br>**CV death or MI**:<br>Men, 0.73 (0.63-0.86)<br>Women, 1.13 (0.89-1.43)	In this meta-analysis comparing a selective and routine invasive strategy, benefit from an routine invasive strategy during long-term follow-up was confirmed in men. Conversely, in women, there was no evidence of benefit.
O'Donoghue M et al, 2012,<br>PMID: 22766336	**9 RCTs**:<br>TACTICS–TIMI 18 (6 months);<br>VINO (6 months);<br>TIMI IIIB (1 year);<br>MATE (1 year);<br>VANQWISH (1 year);<br>FRISC-II (1 year);<br>RITA 3 (1 year);<br>ICTUS (1 year);<br>OASIS-5 (1 year)	1789	1 year	(1) RCT<br>(2) NSTE-ACS<br>(3) Stratified by DM status	RR, 95% CI	**Death**:<br>DM: 1.01 (0.70-1.45)<br>no DM: 1.00 (0.68-1.48)<br>**MI**:<br>DM: 0.71 (0.55–0.92);<br>no DM: 0.98 (0.74–1.29)<br>**Rehospitalization**:<br>DM: 0.75 (0.61–0.92);<br>no DM: 0.75 (0.61–0.93)<br>**Death, MI, or rehospitalization with ACS**:<br>DM, 0.87 (0.73-1.03)<br>no DM, 0.86 (0.70-1.06)	An early invasive strategy yielded similar RR reductions in overall cardiovascular events in diabetic and nondiabetic patients. However, an invasive strategy appeared to reduce recurrent nonfatal MI to a greater extent in diabetic patients. These data support the updated guidelines that recommend an invasive strategy for patients with DM and NSTE-ACS.
Damman P et al, 2012,<br>PMID: 21930723	**3 RCTs**:<br>FRISC-II (5 years);<br>ICTUS (5 years);<br>RITA 3 (5 years)	5467	5 years	NA	HR, 95% CI	**CV Death**:<br>overall, 0.83 (0.68-1.01);<br>65-74 years: 0.78 (0.57-1.07);<br>≥75 years: 0.80 (0.58-1.11);<br><65 years: 0.80 (0.58-1.11)<br>**MI**:<br>overall, 0.83 (0.71-0.97);<br>65-74 years: 0.69 (0.53-0.90);<br>≥75 years: 0.60 (0.43-0.83);<br><65 years: 1.14 (0.90-1.45)<br>**CV death or MI**:<br>overall, 0.86 (0.76-0.98)<br>65-74 years, 0.72 (0.58-0.90)<br>≥75 years, 0.71 (0.55-0.91)<br><65 years, 1.11 (0.90-1.38)	The long-term benefit of the routine invasive strategy over the selective invasive strategy is attenuated in younger patients aged <65 years and in women by the increased risk of early events which seem to have no consequences for long-term cardiovascular mortality. No other clinical risk factors were able to identify patients with differential responses to a routine invasive strategy.

Hoenig MR et al, 2010,<br>PMID: 20238333	**5 RCTs**:<br>TACTICS-TIMI 18 (1, 6 months);<br>VINO (1, 6 months);<br>ICTUS (4 years);<br>FRISC-II (5 years);<br>RITA 3 (5 years)	7818	Early: ≤4 months<br>Intermediate: 6 to 12 months<br>Late: 2 to 5 years	(1) RCT<br>(2) NSTE-ACS<br>(3) Trials undertaken in era of stents	RR, 95% CI	**Death**:<br>≤4 months: 1.11 (0.66-1.88)<br>6 to 12 months: 0.82 (0.57-1.19)<br>2 to 5 years: 0.90 (0.76-1.08)<br>**MI**:<br>≤4 months: 0.64 (0.38-1.06);<br>6 to 12 months: 0.73 (0.62-0.86);<br>2 to 5 years: 0.78 (0.67-0.92)<br>**Rehospitalization**:<br>≤4 months: 0.60 (0.41-0.88);<br>6 to 12 months: 0.67 (0.61-0.74)<br>**Bleeding**: 1.71 (1.27-2.31)<br>**In-hospital death**: 1.59 (0.96-2.64)<br>**Death or MI**:<br>≤4 months 0.64 (0.45-0.92)<br>6 to 12 months 0.76 (0.62-0.94)<br>2 to 5 years 0.89 (0.73-1.08)<br>**In-hospital death**: 1.59 (0.96-2.64)	Compared to a conservative strategy for NSTE-ACS, an invasive strategy is associated with reduced rates of refractory angina and rehospitalization in the shorter term and myocardial infarction in the longer term.
Fox KA et al, 2010,<br>PMID: 20359842	**3 RCTs**:<br>FRISC-II (5 years);<br>ICTUS (5 years);<br>RITA 3 (5 years)	5467	5 years	(1) RCT<br>(2) NSTE-ACS<br>(3)5-year outcomes	HR, 95% CI	**CV death or MI**: 0.81 (0.71-0.93)	A routine invasive strategy reduces long-term rates of cardiovascular death or MI and the largest absolute effect in seen in higher risk patients.
O'Donoghue M et al, 2008,<br>PMID: 18594042	**8 RCTs**:<br>TACTICS-TIMI 18 (6 months);<br>VINO (1 year);<br>TIMI IIIB (1 year);<br>MATE (1 year);<br>VANQWISH (1 year);<br>FRISC-II (1 year);<br>RITA 3 (1 year);<br>ICTUS (1 year)	10412	1 year	(1) RCT<br>(2) NSTE-ACS	OR, 95% CI	**Death**:<br>overall, 0.97 (0.71-1.32)<br>women, 1.11 (0.72-1.70)<br>men, 0.89 (0.58-1.35)<br>**MI**:<br>overall, 0.84 (0.63-1.12);<br>women, 0.93 (0.59-1.45);<br>men, 0.81 (0.59-1.11)<br>**Rehospitalization**:<br>overall, 0.68 (0.55-0.84);<br>women, 0.68 (0.54-0.85);<br>men, 0.66 (0.54-0.82)<br>**Death, MI, or ACS**:<br>overall, 0.78 (0.61-0.98)<br>women, 0.81 (0.65-1.01)<br>biomarker-positive women, 0.67 (0.50-0.88)<br>biomarker-negative women, 0.94 (0.61-1.44)<br>men, 0.73 (0.55-0.98)<br>biomarker-positive men, 0.56 (0.46-0.67)<br>biomarker-negative men, 0.72 (0.51-1.01)	In NSTE-ACS, an invasive strategy has a comparable benefit in men and high-risk women for reducing the composite end point of death, MI, or rehospitalization with ACS.

Bavry AA et al, 2006, PMID: 17010789	**7 RCTs**: ISAR-COOL (1 month); TACTICS-TIMI 18 (6 months); VINO (6 months); TRUCS (1 year); ICTUS (1 year); FRISC-II (2 year); RITA (5 year);	8375	1 month to 5 years	(1) RCT (2) NSTE-ACS (3) Trials undertaken in era of stents and glycoprotein IIb/IIIa inhibitors or thienopyridines	RR, 95% CI	**Death**: 1 month, 0.82 (0.50-1.34); 2 years (mean), 0.75 (0.63-0.90) **MI**: 1 month, 0.93 (0.73-1.19); 2 years (mean), 0.83 (0.72-0.96) **Rehospitalization**: 13 months (mean), 0.69 (0.65-0.74) **Recurrent UA**: 0.69 (0.65-0.74)	Managing non–ST-segment elevation acute coronary syndromes by early invasive therapy improves long-term survival and reduces late myocardial infarction and rehospitalization for unstable angina.
Mehta SR et al, 2005, PMID: 15956636	**7 RCTs**: TIMI IIIB (6 weeks); TACTICS-TIMI 18 (6 months); VINO (6 months); FRISC-II (1 year); VANQWISH (1 year); RITA 3 (2 years); MATE (2 years)	9212	17 months (mean)	(1) RCT (2) NSTE-ACS	OR, 95% CI	**Death**: 0.92 (0.77-1.09) **MI**: 0.75 (0.65-0.88) **Rehospitalization**: 0.66 (0.60-0.72) **In-hospital death**: 1.60 (1.14-2.25) **Death or MI**: 0.82 (0.72-0.93)	A routine invasive strategy exceeded a selective invasive strategy in reducing MI, severe angina, and rehospitalization over a mean follow-up of 17 months. But routine intervention was associated with a higher early mortality hazard and a trend toward a mortality reduction at follow-up.

NSTE-ACS, non–ST-segment elevation acute coronary syndrome; ACS, acute coronary syndrome, MI, myocardial infarction; UA, unstable angina; DM, diabetes mellitus; CV, cardiovascular; RCTs, randomized controlled trials; RR, risk ratio; OR, odds ratio; HR, hazard ratio.

Figure S32. Forest plots of outcomes in the subgroup of the enrollment year greater than 2007. (A) MACE, and (B) Death.

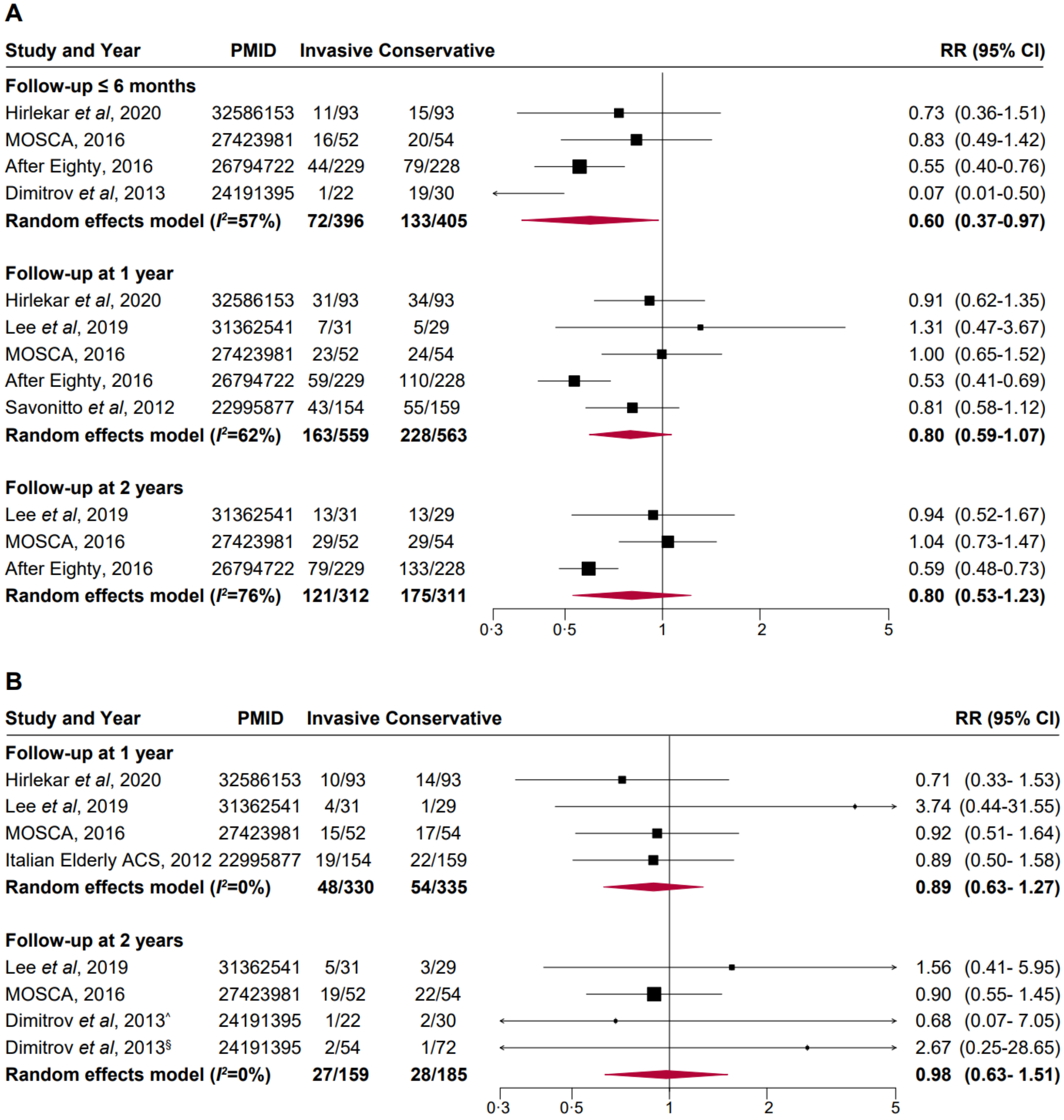

^ Diabetic group, § non-diabetic group.

Figure S33. Forest plots of outcomes in the subgroup of the enrollment year less than 2007. (A) MACE, (B) Death, (C) MI, (D) CV death, (E) Rehospitalization, and (F) Bleeding.

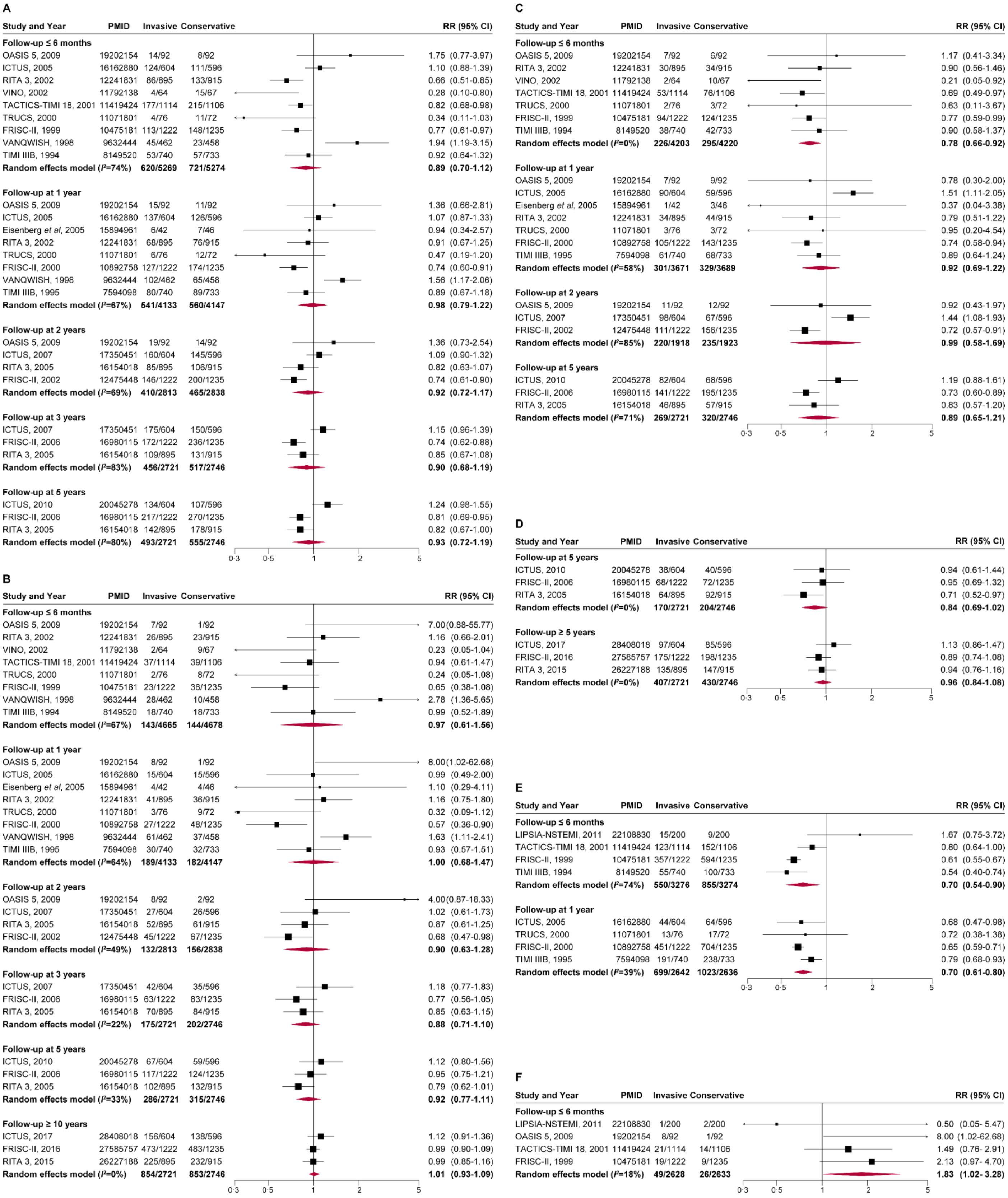

Figure S34. Forest plots of outcomes in the sub analysis excluding the small studies (less than 200 patients). (A) MACE, (B) Death, (C) MI, (D) CV death, (E) Rehospitalization, and (F) Bleeding.

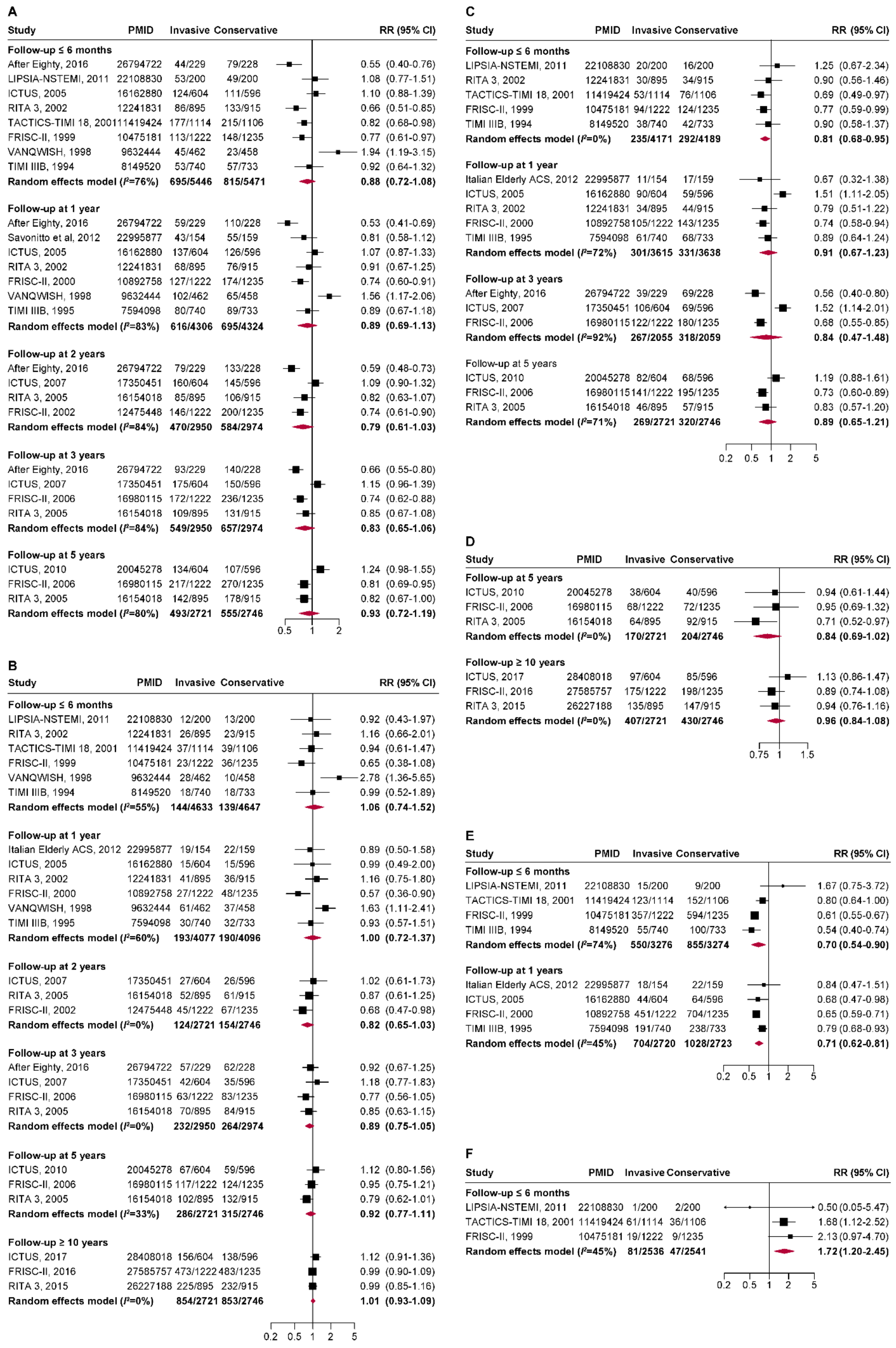

Supplement: Supplementary file 1 [file Presentation1.pdf]
